# Global hospital-based disease management of acute diverticulitis: a prospective, international cohort study

**DOI:** 10.1016/j.eclinm.2025.103548

**Published:** 2025-09-30

**Authors:** DAMASCUS Collaborative, A Lawgaly Sami, A. Monib Fatma, Aakif Muhammad, Ababneh Hazim, Ababneh Malak, Abakar Mourtada, Abd elhady Hamdy, Abdel-Aziz Mohamed, Abdelbaset Mai, Abdel-Maboud Mohamed, Abdel-Maboud Mohamed, Abdelmalak Mina Ragaa Fekry, Abd-elsalam Sherief, Abdelzaher Abdurrahman, Abdul Aal Yasser, Abdulwahed Eman, Abete Roberta, Abo Ali Amira, Abotaleb Khadega, Abu Elnaga Nagm Eldin, Abu Hmaid Amer, Dima Y. Abu Ismail, Abu mahfouz Dema, Abu nawas Duaa, Abu Selmiyh Hala, Abu-Ismail Luai, Abuleil Amro, Mahmoud S. Abwini, Acharya Shivanie, Acosta Lina, Adam Alexis, Adams Katie, Adams Clare, Adegbola Samuel, Adel Jabr Bin Jabr Ala'a, Adel Mohammed Yasmine, Adeyeye Ademola, Adeyeye Rebecca, Adiamah Alfred, Adwi Mohamed, Afify Emma, Afzal Mohamed, Ahmad Shahrukh, Ahmad Bisan, Ahmed Rawan, Ahmed Rizwan, Ahmed Nauman, Ahmed Jamil, Ahonkhai Irele-Ifijeh, Aigner Felix, Ainsworth Paul, Akgun Erhan, Akin Emrah, Akingboye Akinfemi, Akinmade Akinola, Akmercan Ahmet, Aktimur Yunus, Aktokmakyan Talar, Al Daif Alla Abdallah, Al Hashemi Mohamad, Aladini Mohammed, Alajandro Artega Sanchez, Alaklook Safa, Alaklouk Marwa, Alam Mushfique, Al-Amiedy Zaid, Alan Koylu Zehra, Alarood Salameh, Alawashreh Mohammad, Alazzaq Youssef, Albakry Rudaina, Albarki Akram, Albarracín Marín-Blázquez Antonio, Albendary Mohamed, Albirnawi Hatim, Al-dhaheri Manal Jamal, Aldressi Wafa, Aldressi Sarah, Alemad Shada, Al-Eryani Fatima, Alfatih Hamza Mohamed, Alghazawi Laith, Algul Begum, Alhabil Belal, Alhasy Naser, Al-Hayek Tahani, Ali Roshneen, Ali Alshareea Entisar Ahmed, Ali karar Ali Adil, Aljaiuossi Anas, Alkaseek Akram, Alkhaldi Muzan, Al-Kholey Ahmed Emad, Alkikle Reem, Al-Kubati Waheeb, Allam Mohamed, Al-Lamee Noor, Allmer Caterina, Allocco Roberto, Allon Oliver, Alma'aitah Fares, Almaghrebi Asem, Almallah Ahmed, Almi'ani SARI, Al-Nagga Hamza, Al-nahwi Ghofran, AlNajem Hafssah, Alnuweiri Manal, Alqadi Zaid, Alqady Eithar, Al-Qasrawi Shahd, Alqudah Majdi, Al-Sadawi Mohammed, Alsadek Mohamed, Alser Mohammed, Alshaikh Bushra, Al-Shehari Mohammed Mohammed, Alsulaim Hatim, Altinel Yuksel, Altintoprak Fatih, Altiti Raed, Altomare Donato F, Altubi Ikhtiyar, Alvarez-Bautista Francisco, Al-Wahedi Abdulwahid, Al-wajeeh Ghadeer, Al-Wandi Amna, Alwhouhayb Maitham, Alzahran Ayham, Amer Mostafa, Amir Farhat, Ammar Ahmed, Amprayil Mathew, Amro Sarah, Anan Asmaa, Anestiadou Elissavet, Annese Sergio, Annicchiarico Alfredo, Apestegui Carlos Alejandro, Aremu Muyiwa, Arkadopoulos Nikolaos, Arslan Kemal, Ashcroft James, Assaf Nazrin, Assaker Jordan, Avellaneda Nicolas, Ahmed K. Awad, Awadallah lsam, Awbakh Mirna, Aybar Engin, Ayeni Adewale, Ayorinde Tobi, Ayoub Kusay, Babikir Mohammed A, Badenoch Thomas, Bader Franz, Badiani Sarit, Badr Helmy, Badran Yousef Sameh, Badrinath Krishnamurthy, Baeza Murcia Melody, Baggaley Alice, Baig Mirza, Bailey James, Baili Efstratia, Bakewell Zoe, Bakheit Imad M, Balasubramanya Supriya, Balık Emre, Baloyiannis Ioannis, Banks Jessica, Baran Elif, Barbaro Antonio, Barker Jonathan, Barlow Emma, Barnes Thomas, Bartsch Claudia, Bashir Manahil, Bassiony Mahmoud, Batra Paras, Bauza-Collado Mireia, Bayhan Zulfu, Bayraktar Yahya Alperen, Bayram Onur, Beddy David, Belhasan Anas, Bell Zara, Beltrán de Heredia Juan, Belvedere Angela, Benavides Buleje Jorge Alejandro, Benitez-Riesco Ana, Bennett Henry, Ben-Sassi Abozed, Bergmann Nicole, Bermejo Marcos Elena, Berney Christophe, Betoret Lidia, Bevier-Rawls Elyse, Bhasin Swati, Bhasin Deepika, Bhatta Gakul, Bhattacharya Pratik, Biala MarwaIsa, Bianco Francesco, Bierton Christopher, Bilton Henry, Binder Alf-Dorian, Birrer Dominique, Blake Iain, Blázquez-Martín Alma, Bleakley Anna, Bogdan Monica, Bonasso Carlotta, Bonati Elena, Bond Richard, Bond-smith Giles, Bonner Clare, Bonomi Alessandro Michele, Borakati Aditya, Borghi Felice, Borowski David W, Boura Maria, Boushnaq Mohammed, Boutros Marylise, Boyes Joshua, Bozbiyik Osman, Bozkurt Mehmet Abdussamet, Bozkurt Emre, Bracale Umberto, Brachini Gioia, Bravo-Avila Hector, Brett Aishling, Broadbent Jack, Broadhurst Damian, Brown Ben, Bruzzese Giuseppe, Bughio Mumtaz, Buijs Louis F, Buwaitel Mohammad, Bylapudi Seshu, Byrne Jim, Cabezudo Guillermo, Cabrera Paulo, Calabrò Marcello, Calikoglu Fikret, Calikoglu Tugba, Caliskan Cemil, Campbell Abigail, Canas-Martinez Angela, Candan Mert, Cannavera Alessandro, Cannavo Maurizio, Cantero Ramon, Capoglu Recayi, Capolupo Gabriella Teresa, Carannante Filippo, Cardia Roberto, Carlini Massimo, Carrasco Prats María Milagros, Carrie Augusto, Carvello Michele, Casali Lorenzo, Casas Felipe, Caserini Ottavia, Castelhano Rute, Castro-Suárez Marta, Catena Fausto, Cayetano Paniagua Ladislao, Cerdán Santacruz Carlos, Cervellera Maurizio, Chadwick Michael, Chang Jessica, Chan-Thu Aye, Chapman Polly, Chappaley Dimitri, Charalabopoulos Alexandros, Chase Thomas, Chatzmichail Theodora, Chautems Roland, Chavarria Nuria, Cheong Julia, Cholewa Hanna, Christodoulou Spyridon, Chui Hom Lap Jeffrey, Chung Alex, Ciabatti Giulia, Cicerchia Pierfranco Maria, Ciftci Ahmet Burak, Cifuentes-Rodenas José Andrés, Cigagna Luca, Cillara Nicola, Ciolli Maria Giovanna, Cipressi Chiara, Cirillo Bruno, Citgez Bulent, Claramonte-Bellmunt Olga, Clark Mhairi, Clifford Rachael, Cohen Hugo, Coladonato Massimiliano, Colak Elif, Colás-Ruiz Enrique, Collera Ormazábal Pablo, Collins Patrick, Colombari Renan Carlo, Connelly Tara, Cooke Fiachra, Corcione Francesco, Corcione Gregorio, Córdova-García Diego, Correa Bonito Alba, Corso Julian, Coşkun Mümin, Costi Renato, Cotronea Carmelo, Crespi Michele, Cribb Benjamin, Crisafi Daniel, Crisafi Daniel, Cross Katie, Crozier Joseph, Cruikshank Naomi, Curl-Roper Thomas, Currò Giuseppe, Curto López Javier, Cuypers Emma, Dale James, D'Aloisio Giordana, Danias Nikolaos, Danwaththa Liyanage Aloka Suwanna, Daoud Mohammed, Darwich Ayman, Dasilva Louise, Däster Silvio, Davakis Spyridon, Matthew G. Davey, Martin S. Davey, David Bryony, Davies Ioan, D'avino Raffaele, Davis Kurt, Davis George Neelankavil, Dawoud Mostafa, Beatriz De Andrés-Asenjo, Charles de Gheldere, Cristina De Padua, De Palma Giovanni Domenico, De Paola Gilda, De Toma Giorgio, Deeknah Abdulqudus, Del Rio Paolo, Delgado Búrdalo Livia, Delimpalta Christina, Demirli Atici Semra, Dhavala Pooja, Di Nuzzo Maria Michela, Di Saverio Salomone, Diab Jason, Diaz Nicolas Romario, Díaz Gómez Daniel, Díaz Pérez Beatriz, Díaz San Andres Beatriz, Dibra Rigers, Dickerson Luke, Díez-Alonso Manuel, Dikicier Enis, Dimitroulis Dimitrios, Farhat V.N. Din, Doganay Emre, Doheim Mohamed Fahmy, Dölzer Lisa, Donigiewicz Urszula, Douba Zain, Doudin Emad, Douglass Ben, Drozdov Evgeniy, Dubois Marc, Dudek Joanna, Dudi-Venkata Nagendra, Duff Sarah, Durán Muñoz-Cruzado Virginia María, Duval Jean-Luc, Dwidar Oliver, Earley Helen, East Simon, Ebrahim Saarah, Ebrahim Mohamed, Edwards Murphy Amy, Ejtehadi Farshid, Ektiren Mehmet, Elhadi Muhammed, El Salawi Omar, El Tohamy Ayman, El Zaafarany Ahmed, El-ashqar Dina, Merihan A. Elbadawy, Elbahnasawy Mohamed, Elbahnasawy Mohamed, El-Dhuwaib Yesar, Elhadi Muhammed, Elfeki Hossam, Elhajdawe Fras, Elkomy Osama, Elniel Mohammed, Elsabagh Abdallah, Elsaid Mirna, Elsayed Ahmed, Elshami Mohaemedraed, Elshennawy Eslam, Elwan Ayman, Emile Sameh, Emmanuel Klaus, En Oh Ke, English Caroline Louise, Enoch Elizabeth, Entwistle-Thompson Alexandra, Epifani Angelo Gabriele, Eraslan Huseyin, Erşen Ogün, Espada Fuentes Francisco Javier, Espi-Macias Alejandro, Essa Tohamy Tarek, Essam Esmail, Estaire-Gómez Mercedes, Fabbri Nicolò, Fakhrul-Aldeen Mohamed, Fannon Noor, Fannon Aseel, Fardanesh Armin, Farquharson Barnaby, Faulkner Gemma, Faux Will, Fellows David, Carlo V. Feo, Feria-González Ana María, Fernández López Lazaro Javier, Fernandez Martínez María, Fetiha Mohammed, Figueroa Rafael, Firat Necatin, Flatman Michael, Flores Clotet Roser, Foley Katarina, Foppa Caterina, Forero-Torres Alexander, Fournier Ian, Fowler Hayley, Francone Elisa, Franklyn Joshua, Franzini Christan, Frasson Matteo, Freed Ebru, Frontali Alice, Frountzas Maximos, G. Sayed Esraa, Gadea-Mateo Ricardo, Galiffa Giampaolo, Gallo Gaetano, Gamal Mohamed, Ganesan Nityanandan, Ganguly Timothy, Garbarino Sabrina, Garcés Palacios Diana Sofía, Garcia Marin Jose Andrés, García Muñoz Patricia, García Septiem Javier, Garcia-Chavez Hector, García-Niebla Jennifer, Gardiner Padraig, Garg Artu, Garofalidou Tatiana, Garoufalia Zoe, Gasser Elisabeth, Gates Zoe, Gattolin Andrea, Gennari Silvia, Gentilli Sergio, Georgiou Konstantinos, Gerdes Stephan, Ghanbari Amir, Ghanem Ahmed, Ghignone Federico, Gialamas Eleftherios, Gijón Moya Fernando, Gill Sonia, Gill Gurjot, Giménez Francés Clara, Gimeno Calvo Francisco Alberto, Giovenzana Marco, Giuffrida Mario, Giuffrida Maria Carmela, Giuliani Domenico, Giuliani Beatrice, Giuliani Antonio, Gómez Díaz Carlos Javier, Gómez-Sanz Tania, Gonullu Emre, González Hernández Sergio, Grandjean Steven, Grassia Sebastiano, Grechenig Michael, Green Suzie, Green Dylan, Grimaldi Sergio, Grosek Jan, Grossi Ugo, Groundwater Ellen, Gruber Ricarda, Grünbart Martin, Guariglia Claudio Antonio, Guboug Ali, Guendil Boumediene, Guerra Bayron, Gulcek Emre, Guler Sertaç Ata, Guneyli Cem, Gupta Sapna, Gupta Vivek, Gürtler Thomas, Gut Anna Eleonora, Guven Onur, Guy Richard, Habash Elham, Hackett James, Häivälä Reetta, Hajirawala Luv, Halle-Smith James, Hamadi Haider, Hamdan Alaa, Hamed Mazin, Hytham K.S. Hamid, Hammad Farah, Hamza Hamza, Hamza Amr, Handa Siddhartha, Harivallavan Nagendiram, Harmantepe Tarik, Harris Dean, Hart Alex, Hasan Dina, Hasırcı İsmail, Hassam Mohamed, Hassan Mohamed Mare'y, Hassan Mohammed, Hayward Abigail, Hearle Joseph, Helley Michael, Hemadasa Niroshini, Henniger Georg, Herbert Geraint, Hernández-Juara Pilar, Herrero Muñoz Irene, Gabriel F. Hess, Hewett Peter, Heywood Nick, Hickey Lorraine, Hijazin Nadeen, Hijazin Marleen, Hill James, Hill Arnold, Hine Rachael, Hmeidan Majedah, Hogan Aisling M, Hollington Paul, Horisberger Karoline, Horwood James, Hosfield Thomas, Hosking Rachel, Howe Louise, Howie Emma, Hoyos-Torres Alejandro, Hsabo Elmuiz, Hudson Victoria, Hughes James, Humayun Quasim, Humes David, Husain Najam, Husain Zain, Hussain Aimatnuddin Husairi, Huth Marcus, Iacomino Alessandro, Iannone Immacolata, Ibrahimli Arturan, Incollingo Paola, Ioannidis Orestis, Iosifidis Pavlos, Iqbal Atif, Isa Alaa, Isleem Wejdan, Ismail Iyad, Issa Mohamed, Izquierdo-Moreno Ana, Jacqmin Geoffrey, Jamal Ghmagh Reem, Javid Zahra, Jayarajah Umesh, Jezieniecki Carlos, Jia Kevin, Jichi Tarik, Jimenez Cristina, Jiménez Carneros Virginia, Jiménez Miramón Francisco Javier, Jimenez-Gomez Luis Miguel, Jiménez-Higuera Elisa, Jobran Rania, Meredith P. Johnson, Johnston Sean, Robert P. Jones, Jones Sian, Jones Andrew, Jorgensen Lars Nannestad, Joshi Heman, Jover Navalón Jose Maria, Jovine Elio, Kacimi Salah Eddine, Kadamani Akram, Kadir Bryar, Kafka-Ritsch Reinhold, Kalogiannis Evangelos, Kampourakis Christos Antonios, Kang Gurpawan, Kang Mandeep, Kanna Sanad, Kanou Loay, Kara Yasin, Karabulut Kerim, Karaca Berkay Enes, Karamitsau Evangeline, Karamitsou Aikaterini, Karategos Athanasios, Kardassis Dimitrios, Karderirinis Irene, Karim Seiver, Karout Lina, Kattakayam Arjun, Kauppila Joonas, Kaur Mandeep, Kaya Tayfun, Kaya Cemal, Kayode-Nissi Victor, Kayyal Mohammed Yasser, Keeler Barrie, Keller Deborah S, Kennett Jessica, Kerin Michael J, Khafagy Wael, Khalifa Alaa, Khalifa Haneen, Khalil Mohammed, Khalil Aoff, Khalil Omar, Khalil Ahmed Aly, Khamees Almu'atasim, Khan Jan, Khan Fatma, Khan Azam, Khan Jim, Khoza Charlene, Kilinc Tuncer Gizem, Kılınç Ahmet, Kleeff Jorg, Klose Johannes, Klyachman Leslie, Klyachman Leslie, Knowles Charles, Knowles Hannah, Ko Emily, Koh Hoey, Kokoropoulos Panagiotis, Kollias Victoria, Komolafe Segun, Kontaxi Ourania, Korkut Mustafa, Koshel Andrey, Košir Jurij Aleš, Košir Božič Tajda, Kotsifa Eugenia, Kouskos Efstratios, Kozman Mathew, Kronberger Irmgard E, Kroon Hidde, Kudchadkar Shantata, Kumar Dileep, Kumar Sidharth, Kyriacou Harry, Kyriakidis Dimitrios, Kyriakopoulos Georgios, Labró Ciurans Meritxell, Lahlooh Raghad Abed-Allateef, Lait Emily, Lam Kit, Landsweerdt Simon, Lapolla Pierfrancesco, Larentzakis Andreas, Larri Samuel, Lasheen Omar, lauricella sara, Lawday Samuel, Ledda Virginia, Lee Katherine, Lee Crystal, Lee Matthew, Lefroy Rebecca, Lena Adriana, Lenzi Elisa, Leonard Eliot, Leventoglu Sezai, Lewis-Lloyd Christopher, Li Nicolas, Liao Christopher, Lieberman Anna, Lim Sean Tee, Lim Jeffrey, Lim Michael, Lima Pinto Francisca, Lippolis Giuseppe, Lisi Giorgio, Liu Jianliang, Livingston Charles, Lloyd Angus, Loganathan Santhosh, Lombardi Raffaele, Longo Alberto, Lopesino González Jose María, López Morales Pedro, Lourido Ana María, Loutzidou Lydia, Luglio Gaetano, Lunevicius Raimundas, Lyons Thomas, Maccabe Thomas, Macleod Anne, MacRury Marion, Maffione Francesco, Magee Sean, Magill Laura, Mahapatra Sunanda, Maher Ciara, Mahmoud Omar, Maiuri Vincenzo, Mäkäräinen-Uhlbäck Elisa, Mäkelä-Kaikkonen Johanna, Malcolm Francesca, Malvaux Philippe, Mansour Bassam, Mantoglu Baris, Manu Nichola, Marano Alessandra, Marco Caricato, Marcu Vasilica, Mariani Nicolò Maria, Marino Fabio, Marinos Spyros, Marks Bertie, Maroli Annalisa, Marra Ester, Marsanic Patrizia, Martin-Arevalo Jose, Martinez Alegre Javier, Martinez-Iglesias Marta, Martinez-Moreno Jose Luis, Mascianà Gianluca, Masetti Michele, Massey Lisa, Massie Eleanor, Math Suraj, Mathur Pawan, Matías-García Belén, Mattar Ahmed, Maung Min, Maurus Christine, Mawhinney Jamie, Mazzotta Erica, Mazzotti Federico, McAvoy Andrew, McAvoy Dean, McClintick Jessica, McClune Anna, McColl Gillian, McCullough Peter, McDermott Frank D, McGuigan Mari-Claire, McMahon Ross, McNair Angus, Md Hashim Mohd Nizam, Mehmood Iftikhar, Mendoza-Moreno Fernando, Menegat Nevio, Meneghini Simona, Mengual-Ballester Monica, Meric Serhat, Merlini Ilenia, Meza Cabrera Maria Del Mar, Mezead Mohmmad, Michail Marina R, Michalopoulos Nikolaos, Migliore Marco, Milano Egidio, Mills Emily, Mingoli Andrea, Mitteregger Martin, Mittermair Christof, Mizrahi Joseph, Mizrahi Joseph, Moctezuma-Velázquez Paulina, Mohamed Dahy Toqa, Mohamed Hussein Aliae, Mohamed Shaymaa Elsayed, Ali Yasen Y. Mohamedahmed, Mohammad Azmi Mohd Azem Fathi, Mohammed Nada Esmaeel, Mohd Yunus Mohamad Fadli, Moitzi Gabriele, Montali Filippo, Monteiro de Barros James, Montemurro Leonardo, Monti Marco, Montroni Isacco, Monzur Farah, Monzur Farah, Moon Jeongyoon, Moore Tom, Morad Afnan, Morales Diaz Samuel, Morello Alessia, Moreno Suero Francisco, Morgan Matthew, Morini Andrea, Moro-Valdezate David, Morrison-Jones Victoria, Morton Alastair, Mosos Monica Briguitte, Mosquera Manuel, Mostafa Muhanad, Mostafa Hasan, Mostafa Ahmed, Mostafa Hasan, Moug Susan, Mpitsianis Stefanos, Msherghi Ahmed, Mulligan Eabhard, Muñoz Camarena Jose Manuel, Muñoz-Sornosa Ernesto, Muratore Andrea, Murgese Alessandra, Murphy Brenda, Musallam Marah, Mustafa Hamid, Myer Adam, Myer Adam, Nafea Ahmed, Naguib Mostafa Mahmoud, Naidoo Kamil, Naik Lesley-Ann, Nair Gavin, Nair Dheepa, Ñañez Pantoja María Alejandra, Napolitano Milena, Nasser Hussam, Naumann David, Navio Ana, Neary Peter, Nervini Andrea, Ng Vivian, Ng Sherwin, Ng Samantha, Nicastro Vincenzo, Nicholson Gary, Nikaj Herald, Nikiteas Nikolaos, Ninkovic Marijana, Nofal Heba, Nolan Ryan, Novak Ivana, novi alessandra, Nowak Kai, Ntampakis George, Nuñez O'Sullivan Sara, Nyssen Marie, O'Brien Stephen, Obrowski Stephanie, Okoth Kelvin, Oldani Massimo, Oldfield Fran, Ollier Misti, Omeroglu Sinan, Ong Daniel, Opocher Enrico, Orangio Guy, Ordoñez Victor Manuel, O'Reilly Colum, Osman Mohmed, Osorio Ramos Alexander, Othman Ahmad Riyad, Othman Muhammad Faeid, Ottl Stephanie, Ouzounidis Nikolaos, Ovejero-Merino Enrique, Owda Saed, Oyanik Ahmet Faruk, Özata Ibrahim Halil, Ozaydın Safa, Özcan Adem, Ozdemir Kayhan, Özden Sabri, Özocak Aysegul Bahar, Özoran Emre, Padilla-Valverde David, Pagano Gianluca, Pagliai Lorenzo, Pagliai Lorenzo, Palios Ifaistion, Palomares-Casasus Sara, Papadoliopoulou Maria, Papaeleftheriou Stavroula, Papagni Vincenzo, Papandrea Matteo, Papazarkadas Xenofon, Paraoan Marius, Pardo Lopez Sara, Parfitt Charlotte, Park Soo Rin, Parlanti Daniele, Parmar Chetan, Parra Baños Pedro Antonio, Passuello Nicola, Pata Francesco, Patel Panna, Patel Mahul, Patel Ben, Patel Meera, Pathmanathan Keren, Paul Emila, Pavão Tiago, Pavesi Franco, Payne Christopher, Pearce Lyndsay, Pegna Victoria, Pellicer Franco Enrique, Peltrini Roberto, Peña Ros Emilio, Peravali Rajeev, Carlos J. Perez, Pérez-Sánchez Luis Eduardo, Perez-Santiago Leticia, Perivoliotis Konstantinos, Perrone Gennaro, Perrone Fabrizio, Perry Rita, Pesce Antonio, Pestrin Olivia, Petracca Gabriele, Pettitt Michala, Pezzolla Francesco, Pezzuto Anna, Phumiphakmethakul Pawanda, Picarella Pietro, Picciariello Arcangelo, Pindozzi Fioralba, Pinkney Thomas, Pinto José, Pipitone Federico Nicoletta sveva, Pirozzi Nello, Pisani Ceretti Andrea, Polat Suleyman, Polyak Tatyana, Ponzo Paola, Pop Ionut, Porfidia Raffaele, Porta Andrea, Pou Macayo Sara, Poulios Efthimios, Pramanik Sanjeev, Pranesh Nagarajan, Preece Ryan, Presl Jaroslav, Prieto Fernando, Primo Vicent, Proud David, Puerari Gian Attilio, Putzu Giaime, Qasim Ahmad, Quddus Abdul, Quiroga-Valcárcel Ana, Quneis Ossaid, Rabie Mohammed, Racine Michael, Raheel Muhammad, Rahman Rafid, Rai Subash, Rajaretnam Niroshini, Rajput Kunal, Rajput Kunal, Ramadan Salma, Ramadan Widad, Ramasamy Sadhasivam, Ramirez Juan Sebastian, Ramirez Natalia, Ramirez Daniel Mauricio, Ramirez Caballero Ester, Ramírez Faraco María, Ramos Rodriguez Jose Luis, Ramos Soler Francisco, Ramsanahie Anthony, Rangaiah Chandrashekar, Rankin Adeline, Rashid Adil, Raskin Elizabeth, Rateb Fares, Ravi Prabhu, Raza Imran, Rees Adam, Reeves Nicola, Reggiori Alberto, Rehman Saad, Rehman Mutee, Reitano Elisa, Rengifo Carla, Rhayim Roaa, Riaz Samreena, Ricciardi Pietro, Riedl Peter, Riente Francesco, Rigby Sarah, Rimmer Lara Jane, Rimonda Roberto, Rivera Castellano Javier, Rocchi Paolo, Vitharanage Srimantha Dewsiri Rodrigo, Rodriguez Pedro, Rodríguez Sánchez Ana, Rojas-Khalil Yesenia, Rollo Alessio, Román Carlos, Romano Angela, Romano Lucia, Romanou Evdokia, Roncone Arturo, Ronellenfitsch Ulrich, Rooney Siobhan, Rotas Ioannis, Rottoli Matteo, Rozwadowski Sophie, Ruiz-Soriano María, Russo Giulia, Éanna J. Ryan, Ryan Jessica, Saavedra Juan David, Sabbar Mohammed, Sabboobeh Sarah, Sabry Fady, Sagar Jayesh, Sagoo Harkiran, Sahin Can, Şahin Alpaslan, Sainz-Hernández Juan, Saleh Ahmed, Saleh Mahmoud, Salgado-Nesme Noel, Salhan Jyoti, Salomone Sara, Salvemini Carlo, Samadov Elgun, Sambucci Daniele, Sami Sharukh, Sammarco Giuseppe, Sammour Tarik, Samy Hossam, Sanchez Estefania, Sánchez Arteaga Alejandro, Sánchez-Gollarte Ana, Sánchez-Peláez Daniel, Sancho-Muriel Jorge, Sanchon Fructuoso Lorena, Santandrea Letizia, Santillan Mateo, Santoro Giulio Aniello, Sapienza Paolo, Sapre Dimple, Sari Ahmet Can, Sarodaya Varun, Sartarelli Lodovico, Sarveswaran Janahan, Sasia Diego, Sauvain Marc-Olivier, Savoie-Hontoria María, Sayad Reem, Scaltrini Francesca, Schiller Philipp, Schirnhofer Jan, Schmid Alexandra, Segalini Edoardo, Seitinger Gerald, Sevdi Salih, Sexton Gerard, Sgrò Alessandro, Shabana Amanda, Shabbir Jamshed, Shahzad Khalid, Shalaby Mostafa, Shams Ola, Shamsher Shilpa, Sharma Natalie, Sharma Khatiwada Aagat, Sharpe Alexandra, Shebli Baraa, Shehada Anas, Mostafa A. Shehata, Shehata Zak, Shihab Oliver, Shinkwin Michael, Sidiropoulos Theodoros, Simeonidis Savvas, Vlad V. Simianu, Simondi Daniele, Şimşek Gürcan, Singhal Tarun, Sinha Ankit, Skotsimara Antonia, Christopher J. Smart, Neil J. Smart, Smerat Mohammad, Smith Dave, Smolarek Sebastian, Sodde Peter, Soh Jien Yen, Soldini Gabriele, Soler Frias Joan Ricard, Somuncu Erkan, Soria Aledo Víctor, Soriano Celine R, Sorocovici Rodica, Soto Montesinos Cristina, Savas D. Soysal, Sperber Jonas, Spinelli Antonino, Stavrou Gregor Alexander, Stefan Samuel, Stefanescu Iulia Alexandra, Stefanova Irena, Steiner Florian, Stokes Emily, Storey Sharon, Stratakis Konstantinos, Stubbs Benjamin, Stucchi Claudia, Suhardja Thomas, Sundhu Matthew, Swamanatham Christie, Syed Ali Waris, Sylla Patricia, Taffurelli Giovanni, Taggarsi Meghana, Talab Tamie, Tallón Aguilar Luis, Tamara Jorge Leonardo, Tamini Nicolò, Tanal Mert, Tanzanu Marta, Tapuria Niteen, Tartas Ruiz Aurea, Tasende-Presedo Marta, Tashan Nashwan, Tatar Ozan Can, Tawfik Ahmed, Tayyem Raed, Testa Valentina, Thaha Mohamed, Theodoropoulou Katerina, Thomas Rhys, Thomas Pradeep, Thomas-Williams Emily, Thompson Jessica, Thoukididou Sarah, Tinoco González Jose, Toale James, Tokocin Merve, Tokocin Onur, Tomažič Aleš, Tonini Valeria, Torkington Jared, Tornese Deborah, Torrado Maria Alejandra, Totis Mauro, Traeger Luke, Travaglio Elisabetta, Triantafyllou Alexandra, Triantafyllou Tania, Trigiante Giuseppe, Tropeano Francesca Paola, Trujillo Jeancarlos, Tuckey Laurel, Tufan Aydin Eray, Tüfekçi Tutku, Tuñon Fequánt Carlota Isabel, Turan Ersin, Turina Matthias, Turk Ismael, Tzovaras George, Can Uc, Ugolini Giampaolo, Uludag Mehmet, Ulutaş Mehmet Eşref, Tevfik Kıvılcım Uprak, Uraiqat Ahmad, Uranitsch Stefan, Uslu Gülberk, Utkan Nihat Zafer, Uyanik Mustafa Safa, Valério Fernando, Valero Camilo, Graciela Valero Navarro, Valle Rubio Ainhoa, Valverde-Mantecón José Miguel, Dayna van de Hoef, Van Vaerenbergh Wim, Vassiliu Pantelis, Vather Ryash, Peter G. Vaughan-Shaw, Nicholas T. Ventham, Vera-Mansilla Cristina, Vescio Giuseppina, Viamontes Ugalde Francisco Eduardo, Vijay Vardhini, Vila-Zárate Cristina, Vimalachandran Dale, Virgilio Edoardo, Vitone Louis, Vitón-Herrero Rebeca, Vitovska Eva, Vrakopoulou Gavriella-Zoi, Vuagniaux Aurelie, Wadham Bianca, Wallner Elisabeth, Wally Rim, Walters Michael, Wan Mokhter Wan Mokhzani, Warrag Ibrahim, Watfah Josef, Watson Eleanor, Watson Henry, Helmut G. Weiss, West Alex, Wheatstone Sarah, Whitehouse Arlo, Widyaningsih Rizky, Wiesler Benjamin, Williams Olatoyosi, Williams Gethin, Williams Katherine, Wilson Megan, Wimmer Angela, Wong Michael Pak-Kai, Wookey Rebecca, Woyton Michal, Wright Deborah, Wyatt James, Yalcinkaya Ali, Yao Lucy, Yassin Nuha, Yeboah Kwasi, Yeoh Adrian, Yeşilyurt Değercan, Yetkin Sitki Gurkan, Yip Cheerong, Yiu Andrew, Yoldas Tayfun, Younis Soha, Yousef A. Yousef, Zafar Muneeb, Zaghloul Karim, Zakaria Andee Dzulkarnaen, Zakaria Zaidi, Zaman Shafquat, Zambon Martina, Zanus Giacomo, Zapsalis Konstantinos, Zattoni Davide, Zazo Aya, Zazo Rama, Zhang Jennifer, Ziyad Ashwaq, Zor Omer Batuhan

**Keywords:** Acute diverticulitis, Surgery, Computed tomography, Antibiotics, Ambulatory care

## Abstract

**Background:**

Acute diverticulitis is a common condition that can be a surgical emergency. Its prevalence is increasing worldwide, however optimal management remains unclear. This study aimed to determine current practice and short-term outcomes globally.

**Methods:**

A prospective, international cohort study of patients presenting with acute diverticulitis to secondary care units was undertaken. Countries were stratified by geographical region and income groups. Patients presenting over a 44-week recruitment period (1/10/2020–31/8/2021) were included. Patient and disease covariates, management and short-term outcomes were captured in a secure web application. The primary outcome of interest was the geographical variation in presentation and treatment. The secondary outcome was treatment success at 30-days.

**Findings:**

6189 patients presenting with acute diverticulitis (confirmed by CT imaging and/or surgical findings) were recruited. 2798 of 6189 patients presented with uncomplicated disease of whom 849 (30.3%) were treated in an ambulatory manner. Overall antibiotic use ranged across geographical regions from 1838 of 1982 patients (92.7%) to 340 of 342 patients (99.4%). Surgical intervention was undertaken in 782 of 6189 patients (12.6%) varying between geographical regions from 29 of 342 patients (8.8%) to 59 of 195 patients (42.8%). 675 of 782 patients underwent resection, of whom 180 (26.6%) underwent formation of a primary anastomosis. 707 of 6189 (11%) patients experienced treatment failure, with an overall 30-day mortality of 2.8% (169/6189 patients). 30-day mortality was higher in patients with complicated disease (142/3391 patients, 4.5%) and in low-middle income units (20/335 patients, 6.2%).

**Interpretation:**

This large, global study reveals significant variation in the management and outcomes of patients presenting to hospital with acute diverticulitis. Antibiotic use and hospital admission for uncomplicated disease was high, and significant variation in stoma formation was observed. Patients presenting in low-middle income units were more likely to undergo emergency surgery and this was associated with a higher 30-day mortality rate.

**Funding:**

10.13039/100018063Bowel Research UK.

**Research in context**

**Evidence before this study**

We reviewed previous evidence by searching PubMed for all studies that looked at the global variation of the management and treatment of acute diverticulitis. We searched from the 1st January 2000 to 1st August 2020 using the search terms “acute diverticulitis” OR “variation” OR “global” OR “outcomes”. This revealed only several small retrospective studies taken from either single countries or small groups of countries. Datasets were often taken from hospital administrative databases rather than real world practice and there were no prospective studies of presentation, management or outcomes for patients presenting globally to hospitals.

**Added value of this study**

This study represents the largest prospective, international cohort analysis of acute diverticulitis management to date, encompassing 6285 patients from 248 hospitals across 48 countries. It highlights considerable global variation in diagnostic and therapeutic approaches, such as the high use of antibiotics, varying rates of CT imaging, and differences in surgical practices, including stoma formation. Importantly, the findings underscore the over-reliance on hospital-based care for uncomplicated disease and the need for broader adoption of ambulatory management and antibiotic stewardship.

**Implications of all the available evidence**

The findings reveal critical opportunities for healthcare systems and policymakers to optimize acute diverticulitis management pathways. Reducing unnecessary antibiotic use and expanding ambulatory care will lead to reduced utilisation of inpatient hospital beds and reduce unnecessary antibiotic use. Optimising surgical care may lead to reduced stoma formation, earlier surgical intervention and thus a reduction in readmission and ultimately improved quality of life for patients. Further studies are required to understand the reasons behind this variation and the limited uptake of the current evidence base.

## Introduction

Diverticulosis is the most common morphological abnormality of the colon and has a prevalence that is increasing globally.^1^ The exact underlying pathogenesis remains poorly understood, although prevalence may increase with age and obesity, affecting over 40% of the population over 60 years and increasing to 80% in those over 80.^2^^,^^3^ Approximately 5% of patients will develop acute diverticulitis^4^ encompassing a spectrum of disease and symptoms ranging from uncomplicated macroscopic inflammation requiring no treatment to a complicated, systemic illness necessitating emergency surgery.

Acute diverticulitis is traditionally thought to affect an elderly, Western demographic, however recent epidemiological studies suggest that the incidence is rising in younger patients, with those aged 18–44 accounting for over 12% of the total.^5^ The global disease profile is also changing, with Asian patients exhibiting a shift from right-sided to “Western” left-sided disease.^6^

The direct and indirect societal costs due to acute diverticulitis are significant and rising, estimated to be greater than $2 billion in the United States each year^7^ where over 200,000 patients are admitted annually, representing a 26% increase between 1998 and 2005.^8^ In the United Kingdom, admission rates have risen from 0.56 to 1.20 per 1000 person years between 1996 and 2006, including a 2.28-fold increase in admissions for perforated disease (approximately 12,000 emergency bowel resections/year).^9^ Complicated disease almost always requires hospital admission and is associated with a high fatality rate of up to 33% at one year.^10^ It is perceived that the burden of this mortality lies within developed nations, but mortality rates are now rising universally. Limited retrospective data from these countries suggests significant variation in the initial treatment of acute diverticulitis which may impact clinical outcomes.^11^

Amidst concerns for reducing pressure on healthcare systems, acute diverticulitis may be amenable to optimization of treatment pathways. International variations in guidelines and clinical practice highlight possibilities for more tailor-made treatment strategies (e.g. need for hospital admission and antibiotics) to reduce resource utilization and improve patient outcomes.^12^, ^13^, ^14^, ^15^ It is unclear whether real world practice adheres to these guidelines; prospective analysis of the current global trends and variations in acute diverticulitis are necessary to explore this.

The Diverticulitis Management: a SnApshot Collaborative aUdit Study (DAMASCUS) collaborative was formed to prospectively study the global management of patients presenting with acute diverticulitis. The primary objective of this first analysis was to describe the global variation in presentation and management. Subsequent analyses will assess the global variation in medium- (6 and 12 months) and long-term (24 months) outcomes.

## Methods

### Study design

An international, multicentre prospective study was performed to collect clinical outcome data from patients presenting to hospital or clinic with acute diverticulitis. The full protocol has been published previously.^16^ DAMASCUS was open to participation by all centres that admit patients with acute diverticulitis. Participation in the collaborative, as well as all tools for data management, were provided for free. The study opened in October 1st 2020, with a rolling start to accommodate disruption caused by the COVID-19 pandemic. New site registration closed on February 28th, 2021; new patient recruitment closed on August 31st, 2021; data collection ceased on December 23rd, 2021. Review of annual admission rates from existing population based studies^8^^,^^9^ suggested a pre-specified sample size of 4000 patients to be sufficient to demonstrate global variation in index presentation and management. Sites were open to recruitment for up to six months, with a minimum recruitment of three cases to be included in the study.

### Study participants

Data were collected from adult patients (18 years and older) presenting to a study site with acute diverticulitis (newly incident within the audit period). Inclusion in the study followed a confirmed diagnosis of acute diverticulitis through CT scan imaging or intraoperatively during emergency surgery. Patients were included regardless of severity of the acute diverticulitis (acute uncomplicated through to fecal peritonitis), mode of presentation (outpatient clinic, inpatient admission, emergency department consultation), or subsequent treatment (admission vs discharge, surgery vs no surgery). Patients who had been previously admitted with acute diverticulitis and then re-presented with a new episode during the study period were also included with time to presentation from prior episodes recorded. Patients with diverticulosis only (the presence of colonic diverticulum without evidence of acute inflammation or complication per CT scan) or bowel perforation due to other confirmed bowel conditions were excluded.

Following identification and inclusion, patients were subsequently classified by the central research team (by review of imaging report or operative note as uncomplicated (defined as the presence of a mildly inflamed segment of colon only) or complicated (defined as an inflamed segment of colon/phlegmon with/without associated complicated features such as abscess formation, localized or generalized free fluid or free air). Complicated cases were then further classified by central review of the CT findings according to the modified Hinchey classification,^17^ stages Ia/Ib representing a phlegmon alone or with associated abscess/localized air. Distant pelvic abscesses were classified as Hinchey II and stages III (purulent peritonitis) and IV (feculent peritonitis) were clarified by central review of the operative findings in those patients’ undergoing surgery.

Short-term (30-day) treatment outcome was defined as ‘successful’ if the patient did not require any escalation of treatment and was discharged. Treatment ‘failure’ was defined as any treatment escalation or reintervention within 30-days of index treatment (including readmission).

### Study variables

After confirming eligibility, patient demographic, diagnostic, disease severity covariates, as well as initial treatment data were collected pseudoanonymously using the electronic case report form (eCRF). Patient identity was protected by ensuring the central research team were firewalled from host institution systems. Recruiting sites were also unable to visualize data from other sites, ensuring patients remained anonymous other to than their own clinical team. Patient variables included age, weight, height, sex, ethnicity,^18^ smoking status, co-morbidities per Charlson Comorbidity Index, Covid-19 status and if Covid-19 impacted the decision making, and country of the hospital. Regions were divided into North America, UK, rest of Europe, Australasia, and rest of world (comprising units from Africa, Asia, Middle East and South America). Countries were also stratified into low-middle (LMIC) or high-upper income (HIC) according to the United Nation Human Development Index (HDI).^19^

Medical variables included vital signs on presentation (temperature, blood pressure, respiratory rate, Q-SOFA Score), baseline laboratory markers (White Cell Count and C-Reactive Protein), disease history (previous admission, time of the last presentation, number of presentations/admissions within the last 12 months, and treatment received over the last 12 months). Diagnostic variables collected were CT and/or operative findings, part of the colon affected, presence of inflammatory stranding, phlegmon mass, abscess (including size, location, and number), free fluid (including volume and location), extra-luminal gas (including volume and location), and visible perforation (including size). Initial treatment choices included ambulatory and inpatient care with antibiotics, percutaneous drainage, or surgery; these treatments could occur in single, combined or sequential regimens.

The main outcome measures of interest were the main disease state and trait characteristics at index presentation, the initial management at index presentation, and the international variability in disease state and management practices. Data have been reported by geographical region rather than individual country to prevent individual unit identification in those countries that had a small number of recruiting sites.

### Data management and analysis

Patient demographic and clinical data were recorded and managed using Research Electronic Data Capture (REDCap)^20^ hosted at Birmingham Centre for Observational and Prospective Studies (BiCOPS). Patients were allocated a unique study number at entry. Sites received secure passwords for designated investigators to log into REDCap. Data management staff checked all CRF data for completeness, data consistency and compliance. A minimum of three patients per site with 95% data completeness rate was required for inclusion. If discrepancies or missing data were identified, staff raised queries with the research team at the participating site. At the end of the data review and cleaning cycle the completeness of the data fields from sites was high (96%). All patient information was included where possible (including partial datasets where appropriate), only three patients were excluded due to unknown disease status.

### Statistical analysis

Descriptive statistics were used to summarize demographic, medical characteristics and outcomes by region. Mean and standard deviation (normally distributed data) or median and inter-quartile range (non-normally distributed data) were used to summarize continuous data, while frequencies and percentages were used to summarize categorical data. Where appropriate global variation in the overall cohort was also reported. For sub-group analysis propensity score matching (using logistic regression model, selecting nearest neighbour with ratio of 3:1 HIC to LMIC matching on sex, smoking status, age and BMI) was used to compare LMIC to HIC units.

### Ethical statement

Ethical approval and patient consent were obtained in those countries where required (Supplementary Table S1). In the United Kingdom following review by the Health Research Authority the study was registered as an audit as no identifiable data were collected, and thus formal consent was not required. The full list of countries that contributed data is available in the acknowledgments.

### Role of the funding source

The funder of the study had no role in study design, data collection, data analysis, data interpretation, or writing of the report.

## Results

6285 patients were recruited between October 1st 2020 and August 31st 2021 from 248 surgical units across 48 countries. Of these, 6189 patients were included in the final analysis (Supplementary Figure S1 demonstrates the reasons behind excluded records). Geographical recruitment was primarily from the UK (3268/6189, 52.8%) and Europe (1982/6189, 32.0%) with smaller contributions from North America (402/6189, 6.5%) and Australasia (342/6189, 5.5%). The remaining patients (195/6189, 3.2%) were recruited from 19 countries from Asia, Africa, South America, and the Middle East (classified as rest of the world); 13 of these were defined as low-middle income based on an HDI of less than 0.79.

Table 1 shows the patient and disease demographics of the whole cohort (uncomplicated and complicated). There was a slight female preponderance and a dominance of Caucasian patients. 1267/6189 (20.7%) of patients were current smokers and 2029/6189 (32.8%) had BMI of over 30 kg/m^2^. Patient and disease demographics were largely similar across geographical regions, although patients in the ‘rest of the world’ were more likely to be non-Caucasian, younger and male (Table 2). Patients from the rest of the world were more likely to have right-sided disease compared to Europe and Australasia or America (22/195, 13.4% vs 13/402–23/342, 3.2%–6.8%).

**Table 1:** Patient and disease characteristic of the whole cohort n = 6189.

Patient characteristics	N = 6189^a^
**Age**	
Age (years)	60 (15)
**Sex**	
Male	2840 (45.9%)
Female	3349 (54.1%)
**Ethnicity**	
White	5506 (90.7%)
Mixed/Multiple ethic groups	180 (3.0%)
Asian/Asian British	122 (2.0%)
Black/African/Caribbean/Black African	85 (1.4%)
Other ethnic group	178 (2.9%)
Missing or NA	118
**Smoking status**	
Never	3497 (57.0%)
Current (within the last six weeks)	1267 (20.7%)
Ex-smoker	1371 (22.3%)
Missing or NA	54
**BMI**	
Median (IQR)	27.8 (24.6, 31.8)
Obese (BMI ≥30 kg/m^2^)	2029 (32.8%)
BMI <30 kg/m^2^	3869 (62.5%)
Missing or NA	291 (4.7%)
**Disease characteristics**	
**Treatment setting**	
Ambulatory	1233 (19.9%)
Admitted	4956 (80.1%)
**Basis of diverticulitis diagnosis**	
CT	6093 (98.4%)
During emergency surgery	95 (1.6%)
(Missing) Or NA	1
**Previous admission/ambulatory care for acute diverticulitis**	
No	4503 (72.8%)
Yes	1685 (27.2%)
Missing	1
**Charlson score**	
None	1354 (21.9%)
Mild	2327 (37.6%)
Moderate	1477 (23.9%)
Severe	1031 (16.7%)
**Q-sofa score**	
0	5607 (90.7%)
1	459 (7.4%)
2	89 (1.4%)
3	25 (0.4%)
Missing	9
**Complicated case**	
No	2798 (45.2%)
Yes	3391 (54.8%)
**Which part of the colon was affected**	
Right	280 (4.6%)
Transverse	85 (1.4%)
Descending	764 (12.4%)
Sigmoid	5014 (81.6%)
Missing	46
**Hinchey score (if complicated n = 3391)**	
Hinchey Ia	1966 (59.5%)
Hinchey Ib	588 (17.8%)
Hinchey II	289 (8.7%)
Hinchey III	319 (9.7%)
Hinchey IV	141 (4.3%)
Missing	88

**Abbreviations:** BMI, body mass index; CT, computed tomography; Q-SOFA, quick Sepsis Related Organ Failure Assessment; SD, standard deviation.

a n (%); Mean (SD).

**Table 2:** Patient and disease characteristics by region.

Characteristic	Australasia, N = 342^a^	North America, N = 402^a^	Rest of Europe, N = 1982^a^	Rest of the world, N = 195^a^	UK, N = 3268^a^
**Ethnicity**					
White	297 (86.8%)	224 (73.4%)	1867 (94.2%)	58 (29.7%)	3060 (94.2%)
Mixed/Multiple ethic groups	12 (3.5%)	16 (5.2%)	61 (3.1%)	56 (28.7%)	35 (1.1%)
Asian/Asian British	12 (3.5%)	11 (3.6%)	9 (0.5%)	20 (10.3%)	70 (2.2%)
Black/African/Caribbean	0 (0.0%)	20 (6.6%)	5 (0.3%)	28 (14.4%)	32 (1.0%)
Other ethnic group	21 (6.1%)	34 (11.1%)	40 (2.0%)	33 (16.9%)	50 (1.5%)
(Missing) or NA	0	97	0	0	21
**Sex**					
Male	158 (46.2%)	193 (48.0%)	1003 (50.6%)	128 (65.6%)	1358 (41.6%)
Female	184 (53.8%)	209 (52.0%)	979 (49.4%)	67 (34.4%)	1910 (58.4%)
**Smoking status**					
Never	187 (54.7%)	276 (68.7%)	1132 (57.5%)	102 (52.6%)	1800 (55.8%)
Current (within the last six weeks)	64 (18.7%)	38 (9.5%)	424 (21.5%)	60 (30.9%)	681 (21.1%)
Ex-smoker	91 (26.6%)	88 (21.9%)	413 (21.0%)	32 (16.5%)	747 (23.1%)
(Missing) or NA	0	0	13	1	40
**Age**					
Age (years)	61 (16)	60 (14)	60 (15)	55 (14)	61 (15)
**BMI**					
Median (IQR)	29 (25, 33)	28 (24, 32)	27 (24, 30)	28 (25, 31)	28 (25, 33)
Obese (BMI ≥30 kg/m^2^)	148 (43.3%)	106 (26.4%)	505 (25.5%)	68 (34.9%)	1202 (36.8%)
BMI <30 kg/m^2^	194 (56.7%)	192 (47.8%)	1410 (71.1%)	122 (62.6%)	1951 (59.7%)
(Missing) or NA	0 (0.0%)	104 (25.9%)	67 (3.4%)	5 (2.6%)	115 (3.5%)
**How was this patient treated**					
Ambulatory	13 (3.8%)	239 (59.5%)	583 (29.4%)	55 (28.2%)	343 (10.5%)
Inpatient	329 (96.2%)	163 (40.5%)	1399 (70.6%)	140 (71.8%)	2925 (89.5%)
**How was acute diverticulitis diagnosed**					
CT	336 (98.2%)	400 (99.5%)	1959 (98.8%)	159 (81.5%)	3231 (98.8%)
During emergency surgery	6 (1.8%)	2 (0.5%)	23 (1.2%)	36 (18.5%)	28 (1.2%)
(Missing) or NA	0	0	0	0	1
**Previous admission/ambulatory care for acute diverticulitis**					
No	232 (67.8%)	218 (54.2%)	1404 (70.8%)	148 (75.9%)	2501 (76.6%)
Yes	110 (32.2%)	184 (45.8%)	578 (29.2%)	47 (24.1%)	766 (23.4%)
(Missing) or NA	0	0	0	0	1
**Charlson score**					
None	76 (22.2%)	80 (19.9%)	437 (22.0%)	43 (22.1%)	718 (22.0%)
Mild	122 (35.7%)	160 (39.8%)	757 (38.2%)	76 (39.0%)	1212 (37.1%)
Moderate	62 (18.1%)	104 (25.9%)	464 (23.4%)	51 (26.2%)	796 (24.4%)
Severe	82 (24.0%)	58 (14.4%)	324 (16.3%)	25 (12.8%)	542 (16.6%)
**Q-sofa score**					
0	305 (89.2%)	371 (93.0%)	1836 (92.8%)	125 (64.1%)	2970 (91.0%)
1	32 (9.4%)	24 (6.0%)	113 (5.7%)	50 (25.6%)	240 (7.4%)
2	5 (1.5%)	3 (0.8%)	25 (1.3%)	12 (6.2%)	44 (1.3%)
3	0 (0.0%)	1 (0.3%)	5 (0.3%)	8 (4.1%)	11 (0.3%)
Missing	0	3	3	0	3
**Complicated case**					
No	152 (44.4%)	229 (57.0%)	730 (36.8%)	69 (35.4%)	1618 (49.5%)
Yes	190 (55.6%)	173 (43.0%)	1252 (63.2%)	126 (64.6%)	1650 (50.5%)
**Which part of the colon was affected**					
Right	23 (6.8%)	13 (3.2%)	93 (4.7%)	22 (13.4%)	129 (4.0%)
Transverse	7 (2.1%)	7 (1.7%)	16 (0.8%)	7 (4.3%)	48 (1.5%)
Descending	39 (11.5%)	74 (18.4%)	269 (13.6%)	35 (21.3%)	347 (10.6%)
Sigmoid	271 (79.7%)	308 (76.6%)	1599 (80.9%)	100 (61.0%)	2736 (83.9%)
Missing	2	0	5	31	8
**Hinchey score**	n = 190	n = 173	n = 1252	n = 126	n = 1650
Hinchey Ia	122 (65.2%)	87 (50.6%)	723 (60.0%)	50 (43.9%)	984 (60.6%)
Hinchey Ib	30 (16.0%)	48 (27.9%)	225 (18.7%)	17 (14.9%)	268 (16.5%)
Hinchey II	18 (9.6%)	20 (11.6%)	88 (7.3%)	11 (9.6%)	152 (9.4%)
Hinchey III	11 (5.9%)	8 (4.7%)	130 (10.8%)	27 (23.7%)	143 (8.8%)
Hinchey IV	6 (3.2%)	9 (5.2%)	40 (3.3%)	9 (7.9%)	77 (4.7%)
(Missing) or NA	3	1	46	12	26

**Abbreviations:** BMI, body mass index; CT, computed tomography; Q-SOFA, quick Sepsis Related Organ Failure Assessment.

a n (%).

**Fig. 1:**
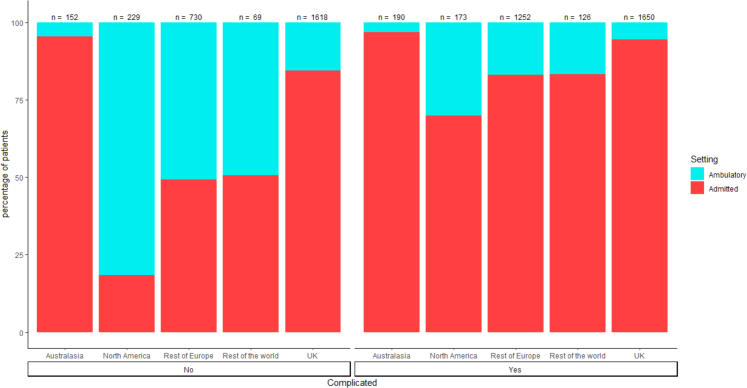
Treatment pathway by geographical location n = 6189.

**Fig. 2:**
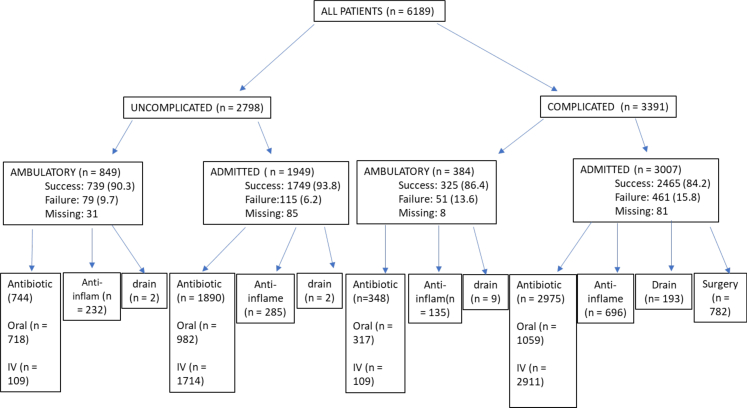
Treatment setting and type by disease status n = 6189.

2798/6189 (45.2%) patients were classified as presenting with uncomplicated disease and 3391/6189 (54.8%) with complicated disease. Fig. 1 demonstrates the global variation in the rates of ambulatory management of both uncomplicated and complicated disease which varied from 3.8% to 59.5% (Table 2) across the whole cohort. Patients with uncomplicated disease (849/2798, 30.3%) were more likely to be treated in an ambulatory manner than complicated disease (384/3391, 11.3%, Fig. 2).

**Fig. 3:**
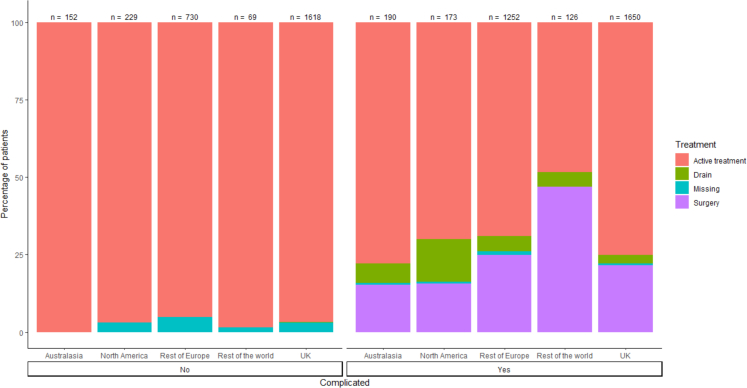
Treatment combination by geographical region n = 6189.

Fig. 2 shows treatment variation according to classification of uncomplicated or complicated disease. Both single and multiple treatment combinations were employed, however antibiotics were prescribed in most patients (5957/6189, 96.6%, range 1838/1982–340/342, 92.7%–99.4%, Table 3) regardless of disease status or treatment setting. In particular, rates of antibiotic use were high in patients with uncomplicated disease (2634/2798, 94.1%, Fig. 2). Fig. 3 demonstrates the variation in treatment combinations by geographical region.

**Table 3:** Treatment variation by geographical region n = 6189.

Characteristic	Australasia, N = 342^a^	North America, N = 402^a^	Rest of Europe, N = 1982^a^	Rest of the world, N = 195^a^	UK, N = 3268^a^	Overall, N = 6189^a^
**Oral Antibiotics**						
No	226 (66.1%)	88 (21.9%)	1203 (60.7%)	113 (58.5%)	1460 (45.0%)	3090 (50.1%)
Yes	116 (33.9%)	314 (78.1%)	779 (39.3%)	80 (41.5%)	1787 (55.0%)	3076 (49.9%)
(Missing) or NA	0	0	0	2	21	23
**IV antibiotics**						
No	13 (3.8%)	244 (60.7%)	576 (29.1%)	50 (25.9%)	441 (13.6%)	1324 (21.5%)
Yes	329 (96.2%)	158 (39.3%)	1406 (70.9%)	143 (74.1%)	2807 (86.4%)	4843 (78.5%)
(Missing) or NA	0	0	0	2	20	22
**Any antibiotic (oral and/or IV)**						
No	2 (0.6%)	8 (2.0%)	144 (7.3%)	5 (2.6%)	51 (1.6%)	210 (3.4%)
Yes	340 (99.4%)	394 (98.0%)	1838 (92.7%)	188 (97.4%)	3197 (98.4%)	5957 (96.6%)
(Missing) or NA	0	0	0	2	20	22
**Anti-inflammatory agent**						
None	330 (96.5%)	337 (83.8%)	1132 (57.1%)	81 (42.0%)	2939 (90.5%)	4819 (78.1%)
Oral agent	12 (3.5%)	65 (16.2%)	829 (41.8%)	92 (47.7%)	303 (9.3%)	1301 (21.1%)
Topical agent	0 (0.0%)	0 (0.0%)	21 (1.1%)	20 (10.4%)	6 (0.2%)	47 (0.8%)
(Missing) or NA	0	0	0	2	20	22
**Percutaneous drain**						
No	326 (95.3%)	377 (93.8%)	1908 (96.3%)	173 (89.6%)	3178 (97.8%)	5962 (96.7%)
Yes	16 (4.7%)	25 (6.2%)	74 (3.7%)	20 (10.4%)	71 (2.2%)	206 (3.3%)
(Missing) or NA	0	0	0	2	19	21
**Was surgery performed?**						
No	300 (91.2%)	136 (83.4%)	1088 (77.8%)	79 (57.2%)	2550 (87.7%)	4153 (84.2%)
Yes	29 (8.8%)	27 (16.6%)	311 (22.2%)	59 (42.8%)	356 (12.3%)	782 (15.8%)
(Missing) or NA	13	239	583	57	362	1254

**Abbreviations:** IV, intravenous.

a n (%).

Significant differences were observed in the treatment of patients in the rest of the world who were less likely to undergo cross sectional imaging prior to surgery and were more likely to be systemically unwell (Supplementary Table S2) and more likely to undergo surgery at admission (Tables 2 and 3). To determine whether this observation was due to observed differences in patient demographics or due to healthcare resources we performed a matched cohort analysis of low-middle income (LMIC) and high-income (HIC) countries according to their HDI (Supplementary Table S3). Patients were matched by age, sex, BMI and smoking status for this analysis (Supplementary Table 4A and B). Patients treated in LMIC units were much more likely to be systemically unwell (Q-SOFA scores of 3 (13/335, 3.9% vs 1/1005, <0.1%) and were more likely to undergo surgery either immediately or within 30-days of admission (85/335, 30.9% vs 118/1005, 14.5%) including for severe contamination (Hinchey stage IV, 13/335, 7.2% vs 17/1005, 3.1%, Table 4).

**Table 4:** Matched cohort analysis of LMIC vs HIC: Cohort characteristics and treatment.

Characteristic	HIC, N = 1005^a^	LMIC, N = 335^a^	Overall, N = 1340^a^	p-value^b^
**Ethnicity**				
White	942 (94.1%)	133 (39.7%)	1075 (80.5%)	
Mixed/Multiple ethic groups	16 (1.6%)	85 (25.4%)	101 (7.6%)	
Asian/Asian British	20 (2.0%)	24 (7.2%)	44 (3.3%)	
Black/African/Caribbean/Black African	7 (0.7%)	29 (8.7%)	36 (2.7%)	
Other ethnic group	16 (1.6%)	64 (19.1%)	80 (6.0%)	
(Missing) or NA	4	0	4	
**Sex**				
Male	596 (59.3%)	199 (59.4%)	795 (59.3%)	
Female	409 (40.7%)	136 (40.6%)	545 (40.7%)	
**Smoking status**				
Never	481 (47.9%)	158 (47.2%)	639 (47.7%)	
Current (within the last six weeks)	335 (33.3%)	111 (33.1%)	446 (33.3%)	
Ex-smoker	189 (18.8%)	66 (19.7%)	255 (19.0%)	
**Age**				
Age (SD)	57 (15)	56 (16)	57 (15)	
**BMI**				
Median (IQR)	28 (25, 32)	28 (25, 31)	28 (25, 32)	
**How was this patient treated**				
Ambulatory	190 (18.9%)	57 (17.0%)	247 (18.4%)	0.43
Admitted	815 (81.1%)	278 (83.0%)	1093 (81.6%)	
**How was acute diverticulitis diagnosed**				
Via multiplanar CT	995 (99.0%)	294 (87.8%)	1289 (96.2%)	<0.001
During emergency surgery	10 (1.0%)	41 (12.2%)	51 (3.8%)	
**Previously admission/ambulatory care for acute diverticulitis**				
No	730 (72.6%)	282 (84.2%)	1012 (75.5%)	<0.001
Yes	275 (27.4%)	53 (15.8%)	328 (24.5%)	
**Complicated case**				
No	444 (44.2%)	135 (40.3%)	579 (43.3%)	0.21
Yes	561 (55.8%)	200 (59.7%)	761 (56.7%)	
**Charlson score**				
None	298 (29.7%)	82 (24.5%)	380 (28.4%)	0.027
Mild	369 (36.7%)	110 (32.8%)	479 (35.7%)	
Moderate	217 (21.6%)	92 (27.5%)	309 (23.1%)	
Severe	121 (12.0%)	51 (15.2%)	172 (12.8%)	
**Q-sofa score**				
0	928 (92.3%)	243 (72.8%)	1171 (87.5%)	<0.001
1	68 (6.8%)	64 (19.2%)	132 (9.9%)	
2	8 (0.8%)	14 (4.2%)	22 (1.6%)	
3	1 (0.1%)	13 (3.9%)	14 (1.0%)	
(Missing) or NA	0	1	1	
**Was a CT performed**				
No	1 (0.1%)	31 (9.3%)	32 (2.4%)	<0.001
Yes	1004 (99.9%)	303 (90.7%)	1307 (97.6%)	
(Missing) or NA	0	1	1	
**Which part of the colon was affected?**				
Right	40 (4.0%)	35 (11.6%)	75 (5.7%)	<0.001
Transverse	12 (1.2%)	9 (3.0%)	21 (1.6%)	
Descending	124 (12.4%)	63 (20.8%)	187 (14.3%)	
Sigmoid	828 (82.5%)	196 (64.7%)	1024 (78.3%)	
(Missing) or NA	1	32	33	
**Hinchey score**	n = 561	n = 200	n = 761	
Hinchey Ia	320 (58.2%)	82 (45.6%)	402 (55.1%)	<0.001
Hinchey Ib	104 (18.9%)	32 (17.8%)	136 (18.6%)	
Hinchey II	54 (9.8%)	13 (7.2%)	67 (9.2%)	
Hinchey III	55 (10.0%)	40 (22.2%)	95 (13.0%)	
Hinchey IV	17 (3.1%)	13 (7.2%)	30 (4.1%)	
(Missing) Or NA	11	20	31	
**Treatment**				
**Oral antibiotics**				
No	490 (48.9%)	233 (70.0%)	723 (54.1%)	<0.001
Yes	513 (51.1%)	100 (30.0%)	613 (45.9%)	
(Missing) or NA	2	2	4	
**IV antibiotics**				
No	197 (19.6%)	48 (14.4%)	245 (18.3%)	0.033
Yes	806 (80.4%)	285 (85.6%)	1091 (81.7%)	
(Missing) or NA	2	2	4	
**Anti-inflammatory agent**				
None	798 (79.6%)	156 (46.8%)	954 (71.4%)	<0.001
Oral agent	203 (20.2%)	152 (45.6%)	355 (26.6%)	
Topical agent	2 (0.2%)	25 (7.5%)	27 (2.0%)	
(Missing) or NA	2	2	4	
**Percutaneous drain**				
No	968 (96.5%)	305 (91.6%)	1273 (95.3%)	<0.001
Yes	35 (3.5%)	28 (8.4%)	63 (4.7%)	
(Missing) or NA	2	2	4	
**Was surgery performed**				
No	695 (85.5%)	190 (69.1%)	885 (81.3%)	<0.001
Yes	118 (14.5%)	85 (30.9%)	203 (18.7%)	
(Missing) or NA	192	60	252	

**Abbreviations:** BMI, body mass index; CT, computed tomography; IQR, inter-quartile range; IV, intravenous; Q-SOFA, quick Sepsis Related Organ Failure Assessment; SD, standard deviation.

a n (%).

b Pearson's Chi-squared test; Fisher's exact test.

782/6189 patients (12.6%) underwent surgical treatment (range 29/342, 8.8%–59/195, 42.8%, Table 3). 687/782 (87.9%) patients undergoing CT imaging prior to surgery and 95/782 (12.1%) were diagnosed during emergency surgery without pre-operative imaging (Supplementary Table S5). Progression to surgery without pre-operative imaging was more common in LMIC units (41/335, 12.2% vs 10/1005, 1.0%, Table 4). Surgery most often involved resection of the affected segment of bowel (675/782, 86.4%) with only a small number of patients undergoing lavage with no resection (89/782, 11.4%, Supplementary Table S5). Of the 675 resections undertaken, 180/675 (26.6%) underwent a primary anastomosis. For the remaining patients who had a stoma formed, this was most often an end colostomy (443/504, 87.9%). Primary anastomosis rates varied across regions ranging from 23/304 (7.6%) in the UK to 12/23 (52.3%) in North America (Supplementary Table S2).

Patients with uncomplicated disease were more likely to experience successful treatment within 30 days compared to complicated disease (2486/2795, 88.9% vs 2791/3391, 82.3%, Table 5). Treatment success rates were similar in uncomplicated groups whether treated in an ambulatory setting or admitted to hospital (739/2795, 90.3% vs 1747/2794, 93.3%, Table 5). Patient characteristics between ambulatory and admitted uncomplicated patients were largely similar (Supplementary Table S6), although admitted patients tended to be slightly older and frailer.

**Table 5:** 30-day outcome for all patients grouped by severity and treatment location.

30-day outcome	All N = 6189	Uncomplicated N = 2795	Complicated N = 3391
Ambulatory N = 1233			
Success	1064 (86.7%)	739 (90.3%)	325 (86.4%)
Failure	130 (10.5%)	79 (9.7%)	51 (13.6%)
Missing	39	31	8
Admitted N = 4956			
Success	4213 (85%)	1747 (93.3%)	2466 (84.2%)
Failure	587 (15%)	125 (6.7%)	462 (15.8%)
Missing	156	75	81
All admission modes			
Success	5277 (88%)	2486 (88.9%)	2791 (82.3%)
Failure	717 (12%)	204 (7.3%)	513 (15.1%)
Missing	195	106	89
Overall 30-day mortality			
Overall mortality	169 (2.8%)	27 (1.0%)	142 (4.5%)
Diverticular disease related 30-day mortality			
Diverticular disease related mortality	100 (1.7%)	5 (0.2%)	95 (2.9%)

The 30-day mortality rate was higher in complicated patients (142/3391, 4.5% vs 27/2795, 1.0%, Table 5) and in LMIC units (20/335, 6.2% vs 20/1005, 2.1%, Supplementary Table S7). Further details of which treatment combination failed, and subsequent treatment escalation are available in Supplementary Figures S1 and S2.

## Discussion

The DAMASCUS study is the largest global prospective study of acute diverticulitis reported to date and confirms significant variation between and within countries in terms of index treatment and outcomes. The population presenting with acute diverticulitis had an over-representation of certain risk factors. For example, in the UK, presentation was more common in women and 1 in 5 patients were current smokers which is significantly greater than the smoking rate in the general population (1 in 8). Contrary to the common perception, most patients were not significantly overweight with BMI's similar to those of the general population reinforcing that factors such as smoking may be partially responsible for the development of acute diverticulitis. The proportion of women presenting was lower in some countries and may reflect aetiological differences or disparities in healthcare access for women in these countries.^21^ Almost one third of patients had previously suffered from an attack of acute diverticulitis in the 12-months prior to enrolment, reaffirming the financial and medical burden these patients represent to healthcare systems.

The reasons behind the treatment variation this are unclear, despite several published guidelines advising on best practice.^12^, ^13^, ^14^, ^15^ However, these guidelines are based on retrospective review of relatively short-term data. The only available prospective studies have been on small scales of specific groups or geographical regions. Furthermore, there are disparities in these guidelines for care and clinical practice internationally e.g. strength of recommendation for avoiding antibiotics, role of follow up colonic imaging. Therefore, there is no globally adopted completely standardised management of diverticular disease. It is worth noting that data collection took place during the Covid-19 pandemic which was associated with significant disruption to healthcare services which may have clearly influenced some of the findings such as diagnostic pathways and admission thresholds.

Having an evidence-based treatment pathway with consensus on the ideal timing, procedure, and platform for management and recovery could result in significant cost savings and improvement in outcomes. With respect to uncomplicated disease, where perhaps the largest burden lies, there are several areas where we have potentially observed significant geographical variation (1) managing uncomplicated disease in an ambulatory setting; (2) managing acute uncomplicated disease without antibiotics initially. In the current economic climate and era of antibiotic overuse and resistance,^22^ we feel that these findings are worthy of attention.

Managing acute uncomplicated disease without antibiotics and in an ambulatory management has perhaps the most robust evidence to date and is a key recommendation in most guidelines. Long-term follow up of the AVOD study at 10 years demonstrated no difference in the recurrence rate, surgical resection rate or quality of life between those patients with acute uncomplicated acute diverticulitis that did or did not receive antibiotics.^23^ Combining this strategy with ambulatory management has significant resource and financial benefits. A recent meta-analysis of studies comparing inpatient to outpatient treatment revealed potential costs savings of 42–82%.^24^ Extrapolated to larger healthcare systems this saving has the potential to be more significant. In our patient cohort, nearly all patients were treated with antibiotic therapy despite 50% having uncomplicated disease. The lowest rate of antibiotic use was seen in mainland Europe, but this was still very high at 92% suggesting that much more work is still required globally to implement research findings and guidelines. Overall, 70% of patients with acute uncomplicated disease were admitted to hospital for treatment. We believe these data support greater focus on the implementation of ambulatory management for selected patients with uncomplicated disease. Current guidelines do not specify a goal for the rate of ambulatory care, but it is our opinion that this should be much higher than the 30% observed in this study.

Although most patients with acute diverticulitis can be managed non-operatively, a small proportion with complicated disease require emergent surgery. CT imaging prior to such surgery was less common in LMIC units where emergent surgery and stoma formation were more common. Previous studies have similarly reported disparities in end stoma formation (for multiple disease indications) between high and low middle-income countries.^25^ The reasons behind these disparities vary, but access to high-quality imaging at all times may play a role which is a key target of the UN 2030 Sustainable Development Goal.^26^ Patients in LMIC were often sicker and may simply not have had time to undergo CT imaging. This observation must be interpreted with caution as the small cohort from LMIC's may not truly represent this demographic as no data are available on patients with acute diverticulitis who do not, or are unable to, seek modern secondary healthcare. Interestingly, within HIC units, the use of CT was notable lower in Australasia and this merits further attention.

Whilst diverticular disease surgery almost always involves resection of the affected segment of bowel, the initial enthusiasm that surrounded laparoscopic lavage^27^ has largely dissipated with only 11% of the whole patient cohort undergoing this procedure. Recent studies report that in stable patients with minimal contamination, resection and primary anastomosis can be safely performed.^28^ However, we observed significant global variation in restoration of gastrointestinal continuity and in particular stoma formation. The reasons for this are unclear and warrant further investigation, but they do not appear to be related to income or resources (e.g. laparoscopic or radiological capability) as the highest rates were observed in the UK and Australasia. We observed a higher rate of surgery in less severe disease (Hinchey I-II) in some countries, this may be related to failed conservative treatment but requires further exploration as to the reasons behind this as these stages can often be managed non-operatively with antibiotics or percutaneous drainage.

With regards to short-term outcomes, in this first analysis we have primarily assessed variation in presentation and treatment and have not adjusted for disease severity, outcomes could thus be influenced or caused by these baseline imbalances. All rates are presented as raw percentages and without adjustment for potential confounders the results should be interpreted with caution. Our future analysis of outcome data will adjust for these potential confounders.

We recognize the limitations in the data presented; the observational design means that a sampling or selection bias is likely to exist as not all geographical regions may have been fully or truly represented. The allocation of cases with an inflammatory phlegmon as ‘complicated’ is also controversial^2^^,^^29^ and may have overestimated the proportion of complicated cases. However given the variation in interpretation of this term, this allocation has ensured that our uncomplicated group are truly cases of mild diverticulitis only with minimal inflammatory changes only and thus any conclusions regarding antibiotic use or ambulatory care can be made more confidently. The inclusion of only CT or surgically proven diverticular disease limits the study as patients undergoing ultrasound or clinical diagnosis were not included. Likewise, we do not have data on all patients within a region treated conservatively or ambulatory and thus we accept that the cohort presented do not fully reflect all patients with diverticular disease. The limitation of only collecting data from a hospital setting also means that we may not have captured data on the ambulatory or non-antibiotic management of patients managed in primary care or local communities. Similarly, we can never fully control for unobserved biases and hidden interaction effects and as such the associations and disparities identified should be treated as hypothesis-generating rather than definite evidence of cause and effect. However, the size and prospective nature of the study does serve to mitigate these issues to some degree. We recognise that for some subsets and/or geographical regions the absolute numbers are small, making it difficult to draw meaningful conclusions in these areas.

This study has confirmed that significant geographical variation exists in the index management of the acute diverticulitis. We have observed significant variation from published guidelines which may be explained by healthcare resource availability, for many observations, the findings require further exploration. We would recommend that specialty associations and healthcare organisations work together to highlight these variations and develop clear patient pathways. This manuscript has only reported the short-term 30-day outcomes and further analysis will assess the medium and long-term outcomes at 6, 12 and 24 months to determine how these variabilities in early care provision may manifest in differences in longer-term patient-level outcomes.

### Manuscript writing group

The study writing group were involved in study design, study coordination, manuscript preparation and editing (Muhammed Elhadi, Gaetano Gallo, Hayley Fowler, Deborah Keller, Charles Knowles, Matthew Lee, Laura Magill, Kelvin Okoth, Francesco Pata, Rita Perry, Thomas Pinkney, Dale Vimalachandran). Bryar Kadir, Kelvin Okoth and Dale Vimalachandran conducted the data analysis. Michala Pettitt and Michael Walters were involved in ensuring data completeness and accuracy. Dale Vimalachandran, Tom Pinkney, Charles Knowles and Bryar Kadir assessed and verified the data and were responsible for ensuring the accuracy of the manuscript. All authors had full access to all the data in the study. All authors read and approved the final version of the manuscript.

## Contributors

The manuscript writing group contributed to the study design and coordination, writing, data interpretation, and critical revision of the manuscript and take full responsibility for the final manuscript. The study management group and country leads contributed to the study dissemination and co-ordination in each country. All other collaborators contributed to data collection and study governance in each site. Further details of all contributing collaborating authors are available in the supplementary material.

## Data sharing statement

Anonymised data are available upon request from the corresponding authors and successful completion of a data sharing agreement through an Application Programming Interface linked to the REDCap data server hosted at the University of Birmingham, Birmingham, UK.

## Declaration of interests

This study was funded by a charitable grant from the Bowel Research UK charity. All authors declare no competing interests.

## Appendix A Supplementary data

Supplementary Material

Study Protocol

Pubmed Citable Author List
