# Corrigendum to ‘Global hospital-based disease management of acute diverticulitis: a prospective, international cohort study’ eClinicalMedicine 2025; published online Sept 29. https://doi.org/10.1016/j.eclinm.2025.103548

**DOI:** 10.1016/j.eclinm.2025.103625

**Published:** 2025-11-03

**Authors:** DAMASCUS Collaborative, A Lawgaly Sami, A. Monib Fatma, Aakif Muhammad, Ababneh Hazim, Ababneh Malak, Abakar Mourtada, Abd elhady Hamdy, Abdel-Aziz Mohamed, Abdelbaset Mai, Abdel-Maboud Mohamed, Abdel-Maboud Mohamed, Abdelmalak Mina Ragaa Fekry, Abd-elsalam Sherief, Abdelzaher Abdurrahman, Abdul Aal Yasser, Abdulwahed Eman, Abete Roberta, Abo Ali Amira, Abotaleb Khadega, Abu Elnaga Nagm Eldin, Abu Hmaid Amer, Dima Y. Abu Ismail, Abu mahfouz Dema, Abu nawas Duaa, Abu Selmiyh Hala, Abu-Ismail Luai, Abuleil Amro, Mahmoud S. Abwini, Acharya Shivanie, Acosta Lina, Adam Alexis, Adams Katie, Adams Clare, Adegbola Samuel, Adel Jabr Bin Jabr Ala'a, Adel Mohammed Yasmine, Adeyeye Ademola, Adeyeye Rebecca, Adiamah Alfred, Adwi Mohamed, Afify Emma, Afzal Mohamed, Ahmad Shahrukh, Ahmad Bisan, Ahmed Rawan, Ahmed Rizwan, Ahmed Nauman, Ahmed Jamil, Ahonkhai Irele-Ifijeh, Aigner Felix, Ainsworth Paul, Akgun Erhan, Akin Emrah, Akingboye Akinfemi, Akinmade Akinola, Akmercan Ahmet, Aktimur Yunus, Aktokmakyan Talar, Al Daif Alla Abdallah, Al Hashemi Mohamad, Aladini Mohammed, Alajandro Artega Sanchez, Alaklook Safa, Alaklouk Marwa, Alam Mushfique, Al-Amiedy Zaid, Alan Koylu Zehra, Alarood Salameh, Alawashreh Mohammad, Alazzaq Youssef, Albakry Rudaina, Albarki Akram, Albarracín Marín-Blázquez Antonio, Albendary Mohamed, Albirnawi Hatim, Al-dhaheri Manal Jamal, Aldressi Wafa, Aldressi Sarah, Alemad Shada, Al-Eryani Fatima, Alfatih Hamza Mohamed, Alghazawi Laith, Algul Begum, Alhabil Belal, Alhasy Naser, Al-Hayek Tahani, Ali Roshneen, Ali Alshareea Entisar Ahmed, Ali karar Ali Adil, Aljaiuossi Anas, Alkaseek Akram, Alkhaldi Muzan, Al-Kholey Ahmed Emad, Alkikle Reem, Al-Kubati Waheeb, Allam Mohamed, Al-Lamee Noor, Allmer Caterina, Allocco Roberto, Allon Oliver, Alma'aitah Fares, Almaghrebi Asem, Almallah Ahmed, Almi'ani SARI, Al-Nagga Hamza, Al-nahwi Ghofran, AlNajem Hafssah, Alnuweiri Manal, Alqadi Zaid, Alqady Eithar, Al-Qasrawi Shahd, Alqudah Majdi, Al-Sadawi Mohammed, Alsadek Mohamed, Alser Mohammed, Alshaikh Bushra, Al-Shehari Mohammed Mohammed, Alsulaim Hatim, Altinel Yuksel, Altintoprak Fatih, Altiti Raed, Altomare Donato F, Altubi Ikhtiyar, Alvarez-Bautista Francisco, Al-Wahedi Abdulwahid, Al-wajeeh Ghadeer, Al-Wandi Amna, Alwhouhayb Maitham, Alzahran Ayham, Amer Mostafa, Amir Farhat, Ammar Ahmed, Amprayil Mathew, Amro Sarah, Anan Asmaa, Anestiadou Elissavet, Annese Sergio, Annicchiarico Alfredo, Apestegui Carlos Alejandro, Aremu Muyiwa, Arkadopoulos Nikolaos, Arslan Kemal, Ashcroft James, Assaf Nazrin, Assaker Jordan, Avellaneda Nicolas, Ahmed K. Awad, Awadallah lsam, Awbakh Mirna, Aybar Engin, Ayeni Adewale, Ayorinde Tobi, Ayoub Kusay, Babikir Mohammed A, Badenoch Thomas, Bader Franz, Badiani Sarit, Badr Helmy, Badran Yousef Sameh, Badrinath Krishnamurthy, Baeza Murcia Melody, Baggaley Alice, Baig Mirza, Bailey James, Baili Efstratia, Bakewell Zoe, Bakheit Imad M, Balasubramanya Supriya, Balık Emre, Baloyiannis Ioannis, Banks Jessica, Baran Elif, Barbaro Antonio, Barker Jonathan, Barlow Emma, Barnes Thomas, Bartsch Claudia, Bashir Manahil, Bassiony Mahmoud, Batra Paras, Bauza-Collado Mireia, Bayhan Zulfu, Bayraktar Yahya Alperen, Bayram Onur, Beddy David, Belhasan Anas, Bell Zara, Beltrán de Heredia Juan, Belvedere Angela, Benavides Buleje Jorge Alejandro, Benitez-Riesco Ana, Bennett Henry, Ben-Sassi Abozed, Bergmann Nicole, Bermejo Marcos Elena, Berney Christophe, Betoret Lidia, Bevier-Rawls Elyse, Bhasin Swati, Bhasin Deepika, Bhatta Gakul, Bhattacharya Pratik, Biala MarwaIsa, Bianco Francesco, Bierton Christopher, Bilton Henry, Binder Alf-Dorian, Birrer Dominique, Blake Iain, Blázquez-Martín Alma, Bleakley Anna, Bogdan Monica, Bonasso Carlotta, Bonati Elena, Bond Richard, Bond-smith Giles, Bonner Clare, Bonomi Alessandro Michele, Borakati Aditya, Borghi Felice, Borowski David W, Boura Maria, Boushnaq Mohammed, Boutros Marylise, Boyes Joshua, Bozbiyik Osman, Bozkurt Mehmet Abdussamet, Bozkurt Emre, Bracale Umberto, Brachini Gioia, Bravo-Avila Hector, Brett Aishling, Broadbent Jack, Broadhurst Damian, Brown Ben, Bruzzese Giuseppe, Bughio Mumtaz, Buijs Louis F, Buwaitel Mohammad, Bylapudi Seshu, Byrne Jim, Cabezudo Guillermo, Cabrera Paulo, Calabrò Marcello, Calikoglu Fikret, Calikoglu Tugba, Caliskan Cemil, Campbell Abigail, Canas-Martinez Angela, Candan Mert, Cannavera Alessandro, Cannavo Maurizio, Cantero Ramon, Capoglu Recayi, Capolupo Gabriella Teresa, Carannante Filippo, Cardia Roberto, Carlini Massimo, Carrasco Prats María Milagros, Carrie Augusto, Carvello Michele, Casali Lorenzo, Casas Felipe, Caserini Ottavia, Castelhano Rute, Castro-Suárez Marta, Catena Fausto, Cayetano Paniagua Ladislao, Cerdán Santacruz Carlos, Cervellera Maurizio, Chadwick Michael, Chang Jessica, Chan-Thu Aye, Chapman Polly, Chappaley Dimitri, Charalabopoulos Alexandros, Chase Thomas, Chatzmichail Theodora, Chautems Roland, Chavarria Nuria, Cheong Julia, Cholewa Hanna, Christodoulou Spyridon, Chui Hom Lap Jeffrey, Chung Alex, Ciabatti Giulia, Cicerchia Pierfranco Maria, Ciftci Ahmet Burak, Cifuentes-Rodenas José Andrés, Cigagna Luca, Cillara Nicola, Ciolli Maria Giovanna, Cipressi Chiara, Cirillo Bruno, Citgez Bulent, Claramonte-Bellmunt Olga, Clark Mhairi, Clifford Rachael, Cohen Hugo, Coladonato Massimiliano, Colak Elif, Colás-Ruiz Enrique, Collera Ormazábal Pablo, Collins Patrick, Colombari Renan Carlo, Connelly Tara, Cooke Fiachra, Corcione Francesco, Corcione Gregorio, Córdova-García Diego, Correa Bonito Alba, Corso Julian, Coşkun Mümin, Costi Renato, Cotronea Carmelo, Crespi Michele, Cribb Benjamin, Crisafi Daniel, Crisafi Daniel, Cross Katie, Crozier Joseph, Cruikshank Naomi, Curl-Roper Thomas, Currò Giuseppe, Curto López Javier, Cuypers Emma, Dale James, D'Aloisio Giordana, Danias Nikolaos, Danwaththa Liyanage Aloka Suwanna, Daoud Mohammed, Darwich Ayman, Dasilva Louise, Däster Silvio, Davakis Spyridon, Matthew G. Davey, Martin S. Davey, David Bryony, Davies Ioan, D'avino Raffaele, Davis Kurt, Davis George Neelankavil, Dawoud Mostafa, Beatriz De Andrés-Asenjo, Charles de Gheldere, Cristina De Padua, De Palma Giovanni Domenico, De Paola Gilda, De Toma Giorgio, Deeknah Abdulqudus, Del Rio Paolo, Delgado Búrdalo Livia, Delimpalta Christina, Demirli Atici Semra, Dhavala Pooja, Di Nuzzo Maria Michela, Di Saverio Salomone, Diab Jason, Diaz Nicolas Romario, Díaz Gómez Daniel, Díaz Pérez Beatriz, Díaz San Andres Beatriz, Dibra Rigers, Dickerson Luke, Díez-Alonso Manuel, Dikicier Enis, Dimitroulis Dimitrios, Farhat V.N. Din, Doganay Emre, Doheim Mohamed Fahmy, Dölzer Lisa, Donigiewicz Urszula, Douba Zain, Doudin Emad, Douglass Ben, Drozdov Evgeniy, Dubois Marc, Dudek Joanna, Dudi-Venkata Nagendra, Duff Sarah, Durán Muñoz-Cruzado Virginia María, Duval Jean-Luc, Dwidar Oliver, Earley Helen, East Simon, Ebrahim Saarah, Ebrahim Mohamed, Edwards Murphy Amy, Ejtehadi Farshid, Ektiren Mehmet, Elhadi Muhammed, El Salawi Omar, El Tohamy Ayman, El Zaafarany Ahmed, El-ashqar Dina, Merihan A. Elbadawy, Elbahnasawy Mohamed, Elbahnasawy Mohamed, El-Dhuwaib Yesar, Elhadi Muhammed, Elfeki Hossam, Elhajdawe Fras, Elkomy Osama, Elniel Mohammed, Elsabagh Abdallah, Elsaid Mirna, Elsayed Ahmed, Elshami Mohaemedraed, Elshennawy Eslam, Elwan Ayman, Emile Sameh, Emmanuel Klaus, En Oh Ke, English Caroline Louise, Enoch Elizabeth, Entwistle-Thompson Alexandra, Epifani Angelo Gabriele, Eraslan Huseyin, Erşen Ogün, Espada Fuentes Francisco Javier, Espi-Macias Alejandro, Essa Tohamy Tarek, Essam Esmail, Estaire-Gómez Mercedes, Fabbri Nicolò, Fakhrul-Aldeen Mohamed, Fannon Noor, Fannon Aseel, Fardanesh Armin, Farquharson Barnaby, Faulkner Gemma, Faux Will, Fellows David, Carlo V. Feo, Feria-González Ana María, Fernández López Lazaro Javier, Fernandez Martínez María, Fetiha Mohammed, Figueroa Rafael, Firat Necatin, Flatman Michael, Flores Clotet Roser, Foley Katarina, Foppa Caterina, Forero-Torres Alexander, Fournier Ian, Fowler Hayley, Francone Elisa, Franklyn Joshua, Franzini Christan, Frasson Matteo, Freed Ebru, Frontali Alice, Frountzas Maximos, G. Sayed Esraa, Gadea-Mateo Ricardo, Galiffa Giampaolo, Gallo Gaetano, Gamal Mohamed, Ganesan Nityanandan, Ganguly Timothy, Garbarino Sabrina, Garcés Palacios Diana Sofía, Garcia Marin Jose Andrés, García Muñoz Patricia, García Septiem Javier, Garcia-Chavez Hector, García-Niebla Jennifer, Gardiner Padraig, Garg Artu, Garofalidou Tatiana, Garoufalia Zoe, Gasser Elisabeth, Gates Zoe, Gattolin Andrea, Gennari Silvia, Gentilli Sergio, Georgiou Konstantinos, Gerdes Stephan, Ghanbari Amir, Ghanem Ahmed, Ghignone Federico, Gialamas Eleftherios, Gijón Moya Fernando, Gill Sonia, Gill Gurjot, Giménez Francés Clara, Gimeno Calvo Francisco Alberto, Giovenzana Marco, Giuffrida Mario, Giuffrida Maria Carmela, Giuliani Domenico, Giuliani Beatrice, Giuliani Antonio, Gómez Díaz Carlos Javier, Gómez-Sanz Tania, Gonullu Emre, González Hernández Sergio, Grandjean Steven, Grassia Sebastiano, Grechenig Michael, Green Suzie, Green Dylan, Grimaldi Sergio, Grosek Jan, Grossi Ugo, Groundwater Ellen, Gruber Ricarda, Grünbart Martin, Guariglia Claudio Antonio, Guboug Ali, Guendil Boumediene, Guerra Bayron, Gulcek Emre, Guler Sertaç Ata, Guneyli Cem, Gupta Sapna, Gupta Vivek, Gürtler Thomas, Gut Anna Eleonora, Guven Onur, Guy Richard, Habash Elham, Hackett James, Häivälä Reetta, Hajirawala Luv, Halle-Smith James, Hamadi Haider, Hamdan Alaa, Hamed Mazin, Hytham K.S. Hamid, Hammad Farah, Hamza Hamza, Hamza Amr, Handa Siddhartha, Harivallavan Nagendiram, Harmantepe Tarik, Harris Dean, Hart Alex, Hasan Dina, Hasırcı İsmail, Hassam Mohamed, Hassan Mohamed Mare'y, Hassan Mohammed, Hayward Abigail, Hearle Joseph, Helley Michael, Hemadasa Niroshini, Henniger Georg, Herbert Geraint, Hernández-Juara Pilar, Herrero Muñoz Irene, Gabriel F. Hess, Hewett Peter, Heywood Nick, Hickey Lorraine, Hijazin Nadeen, Hijazin Marleen, Hill James, Hill Arnold, Hine Rachael, Hmeidan Majedah, Hogan Aisling M, Hollington Paul, Horisberger Karoline, Horwood James, Hosfield Thomas, Hosking Rachel, Howe Louise, Howie Emma, Hoyos-Torres Alejandro, Hsabo Elmuiz, Hudson Victoria, Hughes James, Humayun Quasim, Humes David, Husain Najam, Husain Zain, Hussain Aimatnuddin Husairi, Huth Marcus, Iacomino Alessandro, Iannone Immacolata, Ibrahimli Arturan, Incollingo Paola, Ioannidis Orestis, Iosifidis Pavlos, Iqbal Atif, Isa Alaa, Isleem Wejdan, Ismail Iyad, Issa Mohamed, Izquierdo-Moreno Ana, Jacqmin Geoffrey, Jamal Ghmagh Reem, Javid Zahra, Jayarajah Umesh, Jezieniecki Carlos, Jia Kevin, Jichi Tarik, Jimenez Cristina, Jiménez Carneros Virginia, Jiménez Miramón Francisco Javier, Jimenez-Gomez Luis Miguel, Jiménez-Higuera Elisa, Jobran Rania, Meredith P. Johnson, Johnston Sean, Robert P. Jones, Jones Sian, Jones Andrew, Jorgensen Lars Nannestad, Joshi Heman, Jover Navalón Jose Maria, Jovine Elio, Kacimi Salah Eddine, Kadamani Akram, Kadir Bryar, Kafka-Ritsch Reinhold, Kalogiannis Evangelos, Kampourakis Christos Antonios, Kang Gurpawan, Kang Mandeep, Kanna Sanad, Kanou Loay, Kara Yasin, Karabulut Kerim, Karaca Berkay Enes, Karamitsau Evangeline, Karamitsou Aikaterini, Karategos Athanasios, Kardassis Dimitrios, Karderirinis Irene, Karim Seiver, Karout Lina, Kattakayam Arjun, Kauppila Joonas, Kaur Mandeep, Kaya Tayfun, Kaya Cemal, Kayode-Nissi Victor, Kayyal Mohammed Yasser, Keeler Barrie, Keller Deborah S, Kennett Jessica, Kerin Michael J, Khafagy Wael, Khalifa Alaa, Khalifa Haneen, Khalil Mohammed, Khalil Aoff, Khalil Omar, Khalil Ahmed Aly, Khamees Almu'atasim, Khan Jan, Khan Fatma, Khan Azam, Khan Jim, Khoza Charlene, Kilinc Tuncer Gizem, Kılınç Ahmet, Kleeff Jorg, Klose Johannes, Klyachman Leslie, Klyachman Leslie, Knowles Charles, Knowles Hannah, Ko Emily, Koh Hoey, Kokoropoulos Panagiotis, Kollias Victoria, Komolafe Segun, Kontaxi Ourania, Korkut Mustafa, Koshel Andrey, Košir Jurij Aleš, Košir Božič Tajda, Kotsifa Eugenia, Kouskos Efstratios, Kozman Mathew, Kronberger Irmgard E, Kroon Hidde, Kudchadkar Shantata, Kumar Dileep, Kumar Sidharth, Kyriacou Harry, Kyriakidis Dimitrios, Kyriakopoulos Georgios, Labró Ciurans Meritxell, Lahlooh Raghad Abed-Allateef, Lait Emily, Lam Kit, Landsweerdt Simon, Lapolla Pierfrancesco, Larentzakis Andreas, Larri Samuel, Lasheen Omar, lauricella sara, Lawday Samuel, Ledda Virginia, Lee Katherine, Lee Crystal, Lee Matthew, Lefroy Rebecca, Lena Adriana, Lenzi Elisa, Leonard Eliot, Leventoglu Sezai, Lewis-Lloyd Christopher, Li Nicolas, Liao Christopher, Lieberman Anna, Lim Sean Tee, Lim Jeffrey, Lim Michael, Lima Pinto Francisca, Lippolis Giuseppe, Lisi Giorgio, Liu Jianliang, Livingston Charles, Lloyd Angus, Loganathan Santhosh, Lombardi Raffaele, Longo Alberto, Lopesino González Jose María, López Morales Pedro, Lourido Ana María, Loutzidou Lydia, Luglio Gaetano, Lunevicius Raimundas, Lyons Thomas, Maccabe Thomas, Macleod Anne, MacRury Marion, Maffione Francesco, Magee Sean, Magill Laura, Mahapatra Sunanda, Maher Ciara, Mahmoud Omar, Maiuri Vincenzo, Mäkäräinen-Uhlbäck Elisa, Mäkelä-Kaikkonen Johanna, Malcolm Francesca, Malvaux Philippe, Mansour Bassam, Mantoglu Baris, Manu Nichola, Marano Alessandra, Marco Caricato, Marcu Vasilica, Mariani Nicolò Maria, Marino Fabio, Marinos Spyros, Marks Bertie, Maroli Annalisa, Marra Ester, Marsanic Patrizia, Martin-Arevalo Jose, Martinez Alegre Javier, Martinez-Iglesias Marta, Martinez-Moreno Jose Luis, Mascianà Gianluca, Masetti Michele, Massey Lisa, Massie Eleanor, Math Suraj, Mathur Pawan, Matías-García Belén, Mattar Ahmed, Maung Min, Maurus Christine, Mawhinney Jamie, Mazzotta Erica, Mazzotti Federico, McAvoy Andrew, McAvoy Dean, McClintick Jessica, McClune Anna, McColl Gillian, McCullough Peter, McDermott Frank D, McGuigan Mari-Claire, McMahon Ross, McNair Angus, Md Hashim Mohd Nizam, Mehmood Iftikhar, Mendoza-Moreno Fernando, Menegat Nevio, Meneghini Simona, Mengual-Ballester Monica, Meric Serhat, Merlini Ilenia, Meza Cabrera Maria Del Mar, Mezead Mohmmad, Michail Marina R, Michalopoulos Nikolaos, Migliore Marco, Milano Egidio, Mills Emily, Mingoli Andrea, Mitteregger Martin, Mittermair Christof, Mizrahi Joseph, Mizrahi Joseph, Moctezuma-Velázquez Paulina, Mohamed Dahy Toqa, Mohamed Hussein Aliae, Mohamed Shaymaa Elsayed, Ali Yasen Y. Mohamedahmed, Mohammad Azmi Mohd Azem Fathi, Mohammed Nada Esmaeel, Mohd Yunus Mohamad Fadli, Moitzi Gabriele, Montali Filippo, Monteiro de Barros James, Montemurro Leonardo, Monti Marco, Montroni Isacco, Monzur Farah, Monzur Farah, Moon Jeongyoon, Moore Tom, Morad Afnan, Morales Diaz Samuel, Morello Alessia, Moreno Suero Francisco, Morgan Matthew, Morini Andrea, Moro-Valdezate David, Morrison-Jones Victoria, Morton Alastair, Mosos Monica Briguitte, Mosquera Manuel, Mostafa Muhanad, Mostafa Hasan, Mostafa Ahmed, Mostafa Hasan, Moug Susan, Mpitsianis Stefanos, Msherghi Ahmed, Mulligan Eabhard, Muñoz Camarena Jose Manuel, Muñoz-Sornosa Ernesto, Muratore Andrea, Murgese Alessandra, Murphy Brenda, Musallam Marah, Mustafa Hamid, Myer Adam, Myer Adam, Nafea Ahmed, Naguib Mostafa Mahmoud, Naidoo Kamil, Naik Lesley-Ann, Nair Gavin, Nair Dheepa, Ñañez Pantoja María Alejandra, Napolitano Milena, Nasser Hussam, Naumann David, Navio Ana, Neary Peter, Nervini Andrea, Ng Vivian, Ng Sherwin, Ng Samantha, Nicastro Vincenzo, Nicholson Gary, Nikaj Herald, Nikiteas Nikolaos, Ninkovic Marijana, Nofal Heba, Nolan Ryan, Novak Ivana, novi alessandra, Nowak Kai, Ntampakis George, Nuñez O'Sullivan Sara, Nyssen Marie, O'Brien Stephen, Obrowski Stephanie, Okoth Kelvin, Oldani Massimo, Oldfield Fran, Ollier Misti, Omeroglu Sinan, Ong Daniel, Opocher Enrico, Orangio Guy, Ordoñez Victor Manuel, O'Reilly Colum, Osman Mohmed, Osorio Ramos Alexander, Othman Ahmad Riyad, Othman Muhammad Faeid, Ottl Stephanie, Ouzounidis Nikolaos, Ovejero-Merino Enrique, Owda Saed, Oyanik Ahmet Faruk, Özata Ibrahim Halil, Ozaydın Safa, Özcan Adem, Ozdemir Kayhan, Özden Sabri, Özocak Aysegul Bahar, Özoran Emre, Padilla-Valverde David, Pagano Gianluca, Pagliai Lorenzo, Pagliai Lorenzo, Palios Ifaistion, Palomares-Casasus Sara, Papadoliopoulou Maria, Papaeleftheriou Stavroula, Papagni Vincenzo, Papandrea Matteo, Papazarkadas Xenofon, Paraoan Marius, Pardo Lopez Sara, Parfitt Charlotte, Park Soo Rin, Parlanti Daniele, Parmar Chetan, Parra Baños Pedro Antonio, Passuello Nicola, Pata Francesco, Patel Panna, Patel Mahul, Patel Ben, Patel Meera, Pathmanathan Keren, Paul Emila, Pavão Tiago, Pavesi Franco, Payne Christopher, Pearce Lyndsay, Pegna Victoria, Pellicer Franco Enrique, Peltrini Roberto, Peña Ros Emilio, Peravali Rajeev, Carlos J. Perez, Pérez-Sánchez Luis Eduardo, Perez-Santiago Leticia, Perivoliotis Konstantinos, Perrone Gennaro, Perrone Fabrizio, Perry Rita, Pesce Antonio, Pestrin Olivia, Petracca Gabriele, Pettitt Michala, Pezzolla Francesco, Pezzuto Anna, Phumiphakmethakul Pawanda, Picarella Pietro, Picciariello Arcangelo, Pindozzi Fioralba, Pinkney Thomas, Pinto José, Pipitone Federico Nicoletta sveva, Pirozzi Nello, Pisani Ceretti Andrea, Polat Suleyman, Polyak Tatyana, Ponzo Paola, Pop Ionut, Porfidia Raffaele, Porta Andrea, Pou Macayo Sara, Poulios Efthimios, Pramanik Sanjeev, Pranesh Nagarajan, Preece Ryan, Presl Jaroslav, Prieto Fernando, Primo Vicent, Proud David, Puerari Gian Attilio, Putzu Giaime, Qasim Ahmad, Quddus Abdul, Quiroga-Valcárcel Ana, Quneis Ossaid, Rabie Mohammed, Racine Michael, Raheel Muhammad, Rahman Rafid, Rai Subash, Rajaretnam Niroshini, Rajput Kunal, Rajput Kunal, Ramadan Salma, Ramadan Widad, Ramasamy Sadhasivam, Ramirez Juan Sebastian, Ramirez Natalia, Ramirez Daniel Mauricio, Ramirez Caballero Ester, Ramírez Faraco María, Ramos Rodriguez Jose Luis, Ramos Soler Francisco, Ramsanahie Anthony, Rangaiah Chandrashekar, Rankin Adeline, Rashid Adil, Raskin Elizabeth, Rateb Fares, Ravi Prabhu, Raza Imran, Rees Adam, Reeves Nicola, Reggiori Alberto, Rehman Saad, Rehman Mutee, Reitano Elisa, Rengifo Carla, Rhayim Roaa, Riaz Samreena, Ricciardi Pietro, Riedl Peter, Riente Francesco, Rigby Sarah, Rimmer Lara Jane, Rimonda Roberto, Rivera Castellano Javier, Rocchi Paolo, Vitharanage Srimantha Dewsiri Rodrigo, Rodriguez Pedro, Rodríguez Sánchez Ana, Rojas-Khalil Yesenia, Rollo Alessio, Román Carlos, Romano Angela, Romano Lucia, Romanou Evdokia, Roncone Arturo, Ronellenfitsch Ulrich, Rooney Siobhan, Rotas Ioannis, Rottoli Matteo, Rozwadowski Sophie, Ruiz-Soriano María, Russo Giulia, Éanna J. Ryan, Ryan Jessica, Saavedra Juan David, Sabbar Mohammed, Sabboobeh Sarah, Sabry Fady, Sagar Jayesh, Sagoo Harkiran, Sahin Can, Şahin Alpaslan, Sainz-Hernández Juan, Saleh Ahmed, Saleh Mahmoud, Salgado-Nesme Noel, Salhan Jyoti, Salomone Sara, Salvemini Carlo, Samadov Elgun, Sambucci Daniele, Sami Sharukh, Sammarco Giuseppe, Sammour Tarik, Samy Hossam, Sanchez Estefania, Sánchez Arteaga Alejandro, Sánchez-Gollarte Ana, Sánchez-Peláez Daniel, Sancho-Muriel Jorge, Sanchon Fructuoso Lorena, Santandrea Letizia, Santillan Mateo, Santoro Giulio Aniello, Sapienza Paolo, Sapre Dimple, Sari Ahmet Can, Sarodaya Varun, Sartarelli Lodovico, Sarveswaran Janahan, Sasia Diego, Sauvain Marc-Olivier, Savoie-Hontoria María, Sayad Reem, Scaltrini Francesca, Schiller Philipp, Schirnhofer Jan, Schmid Alexandra, Segalini Edoardo, Seitinger Gerald, Sevdi Salih, Sexton Gerard, Sgrò Alessandro, Shabana Amanda, Shabbir Jamshed, Shahzad Khalid, Shalaby Mostafa, Shams Ola, Shamsher Shilpa, Sharma Natalie, Sharma Khatiwada Aagat, Sharpe Alexandra, Shebli Baraa, Shehada Anas, Mostafa A. Shehata, Shehata Zak, Shihab Oliver, Shinkwin Michael, Sidiropoulos Theodoros, Simeonidis Savvas, Vlad V. Simianu, Simondi Daniele, Şimşek Gürcan, Singhal Tarun, Sinha Ankit, Skotsimara Antonia, Christopher J. Smart, Neil J. Smart, Smerat Mohammad, Smith Dave, Smolarek Sebastian, Sodde Peter, Soh Jien Yen, Soldini Gabriele, Soler Frias Joan Ricard, Somuncu Erkan, Soria Aledo Víctor, Soriano Celine R, Sorocovici Rodica, Soto Montesinos Cristina, Savas D. Soysal, Sperber Jonas, Spinelli Antonino, Stavrou Gregor Alexander, Stefan Samuel, Stefanescu Iulia Alexandra, Stefanova Irena, Steiner Florian, Stokes Emily, Storey Sharon, Stratakis Konstantinos, Stubbs Benjamin, Stucchi Claudia, Suhardja Thomas, Sundhu Matthew, Swamanatham Christie, Syed Ali Waris, Sylla Patricia, Taffurelli Giovanni, Taggarsi Meghana, Talab Tamie, Tallón Aguilar Luis, Tamara Jorge Leonardo, Tamini Nicolò, Tanal Mert, Tanzanu Marta, Tapuria Niteen, Tartas Ruiz Aurea, Tasende-Presedo Marta, Tashan Nashwan, Tatar Ozan Can, Tawfik Ahmed, Tayyem Raed, Testa Valentina, Thaha Mohamed, Theodoropoulou Katerina, Thomas Rhys, Thomas Pradeep, Thomas-Williams Emily, Thompson Jessica, Thoukididou Sarah, Tinoco González Jose, Toale James, Tokocin Merve, Tokocin Onur, Tomažič Aleš, Tonini Valeria, Torkington Jared, Tornese Deborah, Torrado Maria Alejandra, Totis Mauro, Traeger Luke, Travaglio Elisabetta, Triantafyllou Alexandra, Triantafyllou Tania, Trigiante Giuseppe, Tropeano Francesca Paola, Trujillo Jeancarlos, Tuckey Laurel, Tufan Aydin Eray, Tüfekçi Tutku, Tuñon Fequánt Carlota Isabel, Turan Ersin, Turina Matthias, Turk Ismael, Tzovaras George, Can Uc, Ugolini Giampaolo, Uludag Mehmet, Ulutaş Mehmet Eşref, Tevfik Kıvılcım Uprak, Uraiqat Ahmad, Uranitsch Stefan, Uslu Gülberk, Utkan Nihat Zafer, Uyanik Mustafa Safa, Valério Fernando, Valero Camilo, Graciela Valero Navarro, Valle Rubio Ainhoa, Valverde-Mantecón José Miguel, Dayna van de Hoef, Van Vaerenbergh Wim, Vassiliu Pantelis, Vather Ryash, Peter G. Vaughan-Shaw, Nicholas T. Ventham, Vera-Mansilla Cristina, Vescio Giuseppina, Viamontes Ugalde Francisco Eduardo, Vijay Vardhini, Vila-Zárate Cristina, Vimalachandran Dale, Virgilio Edoardo, Vitone Louis, Vitón-Herrero Rebeca, Vitovska Eva, Vrakopoulou Gavriella-Zoi, Vuagniaux Aurelie, Wadham Bianca, Wallner Elisabeth, Wally Rim, Walters Michael, Wan Mokhter Wan Mokhzani, Warrag Ibrahim, Watfah Josef, Watson Eleanor, Watson Henry, Helmut G. Weiss, West Alex, Wheatstone Sarah, Whitehouse Arlo, Widyaningsih Rizky, Wiesler Benjamin, Williams Olatoyosi, Williams Gethin, Williams Katherine, Wilson Megan, Wimmer Angela, Wong Michael Pak-Kai, Wookey Rebecca, Woyton Michal, Wright Deborah, Wyatt James, Yalcinkaya Ali, Yao Lucy, Yassin Nuha, Yeboah Kwasi, Yeoh Adrian, Yeşilyurt Değercan, Yetkin Sitki Gurkan, Yip Cheerong, Yiu Andrew, Yoldas Tayfun, Younis Soha, Yousef A. Yousef, Zafar Muneeb, Zaghloul Karim, Zakaria Andee Dzulkarnaen, Zakaria Zaidi, Zaman Shafquat, Zambon Martina, Zanus Giacomo, Zapsalis Konstantinos, Zattoni Davide, Zazo Aya, Zazo Rama, Zhang Jennifer, Ziyad Ashwaq, Zor Omer Batuhan

In this Article, the following were accidentally omitted from the acknowledgements section:

Nagendra Dudi-Venkata (manuscript writing group).

Association of Coloproctology of Great Britain and Ireland (supporting organisation).

This correction does not alter the main results or the conclusion of the paper. The corrected versions are now as below.

**Manuscript Writing Group**

The study writing group were involved in study design, study coordination, manuscript preparation and editing (Nagendra Dudi-Venkata, Muhammed Elhadi, Gaetano Gallo, Hayley Fowler, Deborah Keller, Charles Knowles, Matthew Lee, Laura Magill, Kelvin Okoth, Francesco Pata, Rita Perry, Thomas Pinkney, Dale Vimalachandran). Bryar Kadir, Kelvin Okoth and Dale Vimalachandran conducted the data analysis. Michala Pettitt and Michael Walters were involved in ensuring data completeness and accuracy. Dale Vimalachandran, Tom Pinkney, Charles Knowles and Bryar Kadir assessed and verified the data and were responsible for ensuring the accuracy of the manuscript. All authors had full access to all the data in the study. All authors read and approved the final version of the manuscript.

