# Supplementary material for: Global hospital-based disease management of acute diverticulitis: a prospective, international cohort study
Source: eClinicalMedicine. 2025 Sep 30;89:103548. doi: 10.1016/j.eclinm.2025.103548 (PMC12512976; doi:10.1016/j.eclinm.2025.103548)

**SUPPLEMENTARY MATERIAL**

**TABLE OF CONTENTS**


| | |
|---|---|
| **Contributors** | pages 2-9 |
| **Supplementary tables** | pages 10-17 |
| **Supplementary figures** | pages 18-20 |

**Contributors**

Study Management Group:

John Ayorinde, Mohamedraed Elshami, Hayley Fowler, Gaetano Gallo, Bryar Kadir, Deborah S Keller, Charles Knowles, Matthew Lee, Laura Magill, Kelvin Okoth, Francesco Pata, Rita Perry, Michala Pettitt, Tom Pinkney, Mohamed Rabie, Tarik Sammour, Nagendra Dudi-Venkata, Dale Vimalachandran, Michael Walters

Manuscript Group:

All authors named in the manuscript group were involved in the design of the study, acquisition and interpretation of the data, drafted and reviewed the manuscript, reviewed and approved the final manuscript and take responsibility and accountability for the published work.

Dale Vimalachandran MD,[1] Charles Knowles PhD,[2] Tom Pinkney MD,[3] Bryar Kadir PhD,[3] Matthew Lee PhD,[3] Deborah S Keller MD,[4] Kelvin Okoth PhD,[3] Nagendra Dudi-Venkata PhD,[5] Muhammed Elhadi MB BCh,[6] Hayley Fowler MRCS,[7] Gaetano Gallo PhD,[8] Francesco Pata PhD,[9] Rita Perry PhD,[3] Michala Pettitt PhD,[3] Michael Walters BSc,[10] Laura Magill PhD[3]

1. Institute of Systems, Molecular and Integrative Biology, University of Liverpool, United Kingdom
2. Queen Mary University of London and the Cleveland Clinic, London, United Kingdom
3. Department of Applied Health Sciences, University of Birmingham, United Kingdom
4. School of Biological and Health Systems Engineering, Arizona State University, Phoenix, United States of America.
5. Epworth Healthcare, Richmond, Victoria 3121, Australia
6. Faculty of Medicine, University of Tripoli, Libya
7. Health Education Northwest, United Kingdom
8. Department of Surgery, Sapienza University of Rome, Rome, Italy
9. Dipartimento di Farmacia e Scienze della Salute e della Nutrizione – DFSSN, Università della Calabria Rende, Italy
10. College of Life Sciences, University of Leicester, United Kingdom

Country Leads:

Amro Abuleil, Mohammed Alser, Alajandro Sanchez Arteaga, Nico Avellaneda, Mohamed Fahmy Doheim, Muhammed Elhadi, Mohamedraed Elshami, Gaetano Gallo, Francesco Pata, Eleftherios Gialamas, Alaa Hamdan, Salah Eddine Kacimi, Lina Karout, Hamza Al-Nagga, Mohammed Rabie, Ulrich Ronellenfitsch, Mahmoud Saleh, Gerard Sexton, Theodoros Sidiropoulos, Mert Tanal, Nagendra Dudi-Venkata.

Collaborating sites:

Argentina: Dr Nicolas Avellaneda, Dr Augusto Carrie, Dr Mateo Santillan, Dr Milena Napolitano(CEMIC).

Australia: Mr David Proud Dr Daniel Crisafi, Mr Adrian Yeoh, Mr Thomas Suhardja, Dr Sonia Gill, Dr Eleanor Watson, Dr Daniel Crisafi, Dr Gurjot Gill(Austin Hospital); Prof. Christophe Berney, Dr Sarit Badiani, Dr Jason Diab, Dr Kevin Jia, Assoc. Prof Matthew Morgan(Bankstown Hospital); Dr Paul Hollington, Dr Abdallah Elsabagh, Dr Mathew Amprayil, Dr Gavin Nair(Flinders Medical Centre); Dr Mathew Kozman, Dr Timothy Ganguly, Dr Mohamed Afzal, Dr Daniel Ong, Dr Charles Livingston(Lyell McEwin Hospital); Prof. Peter Hewett, Mr Benjamin Cribb, Dr Katarina Foley, Dr Christopher Bierton, Dr Victoria Kollias, Dr Antonio Barbaro, Dr Jianliang Liu, Shilpa Shamsher(Queen Elizabeth Hospital); Assoc. Prof Tarik Sammour, Dr Nagendra Dudi-Venkata, Dr Hidde Kroon, Luke Traeger, Dr Ryash Vather(Royal Adelaide Hospital).

Austria: Dr Jan Schirnhofer, Prof. Helmut G. Weiss, Dr Christof Mittermair, Dr Martin Grünbart(Hospital St. John of God); Prof. Felix Aigner, Dr Martin Mitteregger, Dr Stefan Uranitsch, Dr Caterina Allmer, Dr Gerald Seitinger, Dr Elisabeth Wallner, Ms Gabriele Moitzi(Krankenhaus der Barmherzigen Brüder Graz); Dr Irmgard E Kronberger, Dr Marijana Ninkovic, Mrs Nicole Bergmann, Dr Elisabeth Gasser, Dr Reinhold Kafka-Ritsch, Dr Marcus Huth (Medical

University Innsbruck); Dr Jaroslav Presl, Dr Ricarda Gruber, Dr Ivana Novak (Paraselsus Medical University); Dr Claudia Bartsch, Dr Alexandra Schmid, Dr Florian Steiner(Salzkammergut Klinikum Voecklabruck); Dr Alf-Dorian Binder, Dr Peter Riedl, Dr Thomas Gürtler(University Hospital Tulln).

Azerbaijan: Dr Elgun Samadov, Dr Arturan Ibrahimli(Leyla Medical Centre).

Belgium: Dr Philippe Malvaux, Dr Steven Grandjean, Dr Marie Nyssen, Dr Simon Landsweerdt, Dr Iulia Alexandra Stefanescu, Dr Geoffrey Jacqmin, Dr Jordan Assaker(Central Hospital of Wallonie Picarde); Dr Charles de Gheldere, Dr Emma Cuypers, Dr Omar El Salawi, Dr Wim Van Vaerenbergh, Dr Marc Dubois(Heilig Hart Hospital).

Canada: Dr Marylise Boutros, Ms Sarah Sabboobeh, Dr Jeongyoon Moon, Dr Hatim Alsulaim, Dr Ikhtiyar Altubi, Dr Hafssah AlNajem(Jewish General Hospital).

Colombia: Dr Rafael Figueroa, Dr Monica Briguitte Mosos, Dr Juan Sebastian Ramirez, Dr Jorge Leonardo Tamara, Mr Juan David Saavedra, Ms Maria Alejandra Torrado, Mr Victor Manuel Ordoñez, Ms Natalia Ramirez, Mr Nicolas Romario Diaz, Mr Daniel Mauricio Ramirez(Avidanti Ibagué Clinic); Dr Paulo Cabrera, Dr Cristina Jimenez, Dr Lina Acosta, Dr Julian Corso, Dr Carlos J- Perez, Dr Carlos Román, Dr Akram Kadamani, Dr Bayron Guerra, Dr Felipe Casas, Dr Manuel Mosquera(Grupo de Investigación Cirugia Fundacion Cardioinfaitl-IC); Dr Maria Del Mar Meza Cabrera, Dr Diana Sofía Garcés Palacios, Dr María Alejandra Ñañez Pantoja, Dr Ana María Lourido(Susana Lopez of Valencia Hospital).

Denmark: Prof. Lars Nannestad Jorgensen, M.D. Mohamed Ebrahim(Bispebjerg Hospital); Dr Anna Eleonora Gut, Prof. Franz Bader, Ms Stephanie Ottl(Isarklinikum).

Egypt: Prof. Ayman Elwan, Dr Mohamed Hassan, Dr Youssef Alazzaq, Dr Ahmed Almallah, Dr Mostafa Naguib, Dr Alaa Isa, Dr Fares Rateb, Dr Hamza Hamza, Dr Mohamed Alsadek, Dr Ahmed Emad Al-Kholey(Al-Azhar University Hospital, New Damietta); Dr Mohamed Abdel-Aziz, Dr Mahmoud Bassiony, Dr Ahmed Saleh, Dr Toqa Mohamed, Dr Amira Abo Ali, Dr Mohamed Elbahnasawy, Dr Ahmed Nafea, Dr Muhanad Mostafa, Dr Mostafa Shehata, Dr Esmail Essam(Alexandria Main University Hospital ); Dr Nagm Eldin Abu Elnaga, Prof. Aliae Mohamed Hussein, Ms Fatma A. Monib, Ms Eithar Alqady, Mr Tarek Essa Tohamy, Ms Esraa G. Sayed, Ms Yasmine Adel, Ms Afnan Morad, Ms Mai Abdelbaset, Ms Reem Sayad(Assiut University Hospital); Prof. Ayman El Tohamy, Dr Alaa Khalifa, Dr Mohamed Allam, Dr Mohamed Hassam(Banha University Hospital ); Prof. Ahmed Aly Khalil, Mr Abdurrahman Abdelzaher, Ms Salma Ramadan, Mr Yousef Sameh Badran, Ms Marina R Michail, Ms Merihan A. Elbadawy, Mr Ahmed K. Awad, Ms Nada Esmaeel Mohammed, Mr Mostafa Dawoud, Ms Shaymaa Elsayed Mohamed (Ain Shams University Hospitals); Dr Mohamed Abdel-Maboud, Dr Hasan Mostafa(El Hussein University Hospital); Dr Eslam Elshennawy(Kafr Elshikh University Hospital ); Dr Hossam Elfeki, Dr Mostafa Shalaby, Dr Sameh Emile, Dr Mirna Elsaid, Dr Fady Sabry, Prof. Wael Khafagy(Mansoura University); Prof. Hamdy Abd elhady, Dr Helmy Badr, Prof. Mohamed Elbahnasawy, Prof. Sherief Abd-elsalam(Tanta University Hospital); Dr Rim Wally, Dr Bassam Mansour, Dr Ahmed Ghanem, Dr Mohamed Gamal(Suez Canal University Hospitals).

Finland: Prof. Joonas Kauppila, Dr Reetta Häivälä, Dr Elisa Mäkäräinen-Uhlbäck, Dr Johanna Mäkelä-Kaikkonen(Oulu University Hospital).

Germany: Dr Philipp Schiller, Dr. med. univ. Lisa Dölzer, Prof. Kai Nowak(RoMed Clinic Rosenheim); Dr Gregor Alexander Stavrou, Dr Jonas Sperber, Dr Rizky Widyaningsih, Dr Dimitrios Kardassis(Saarbruecken Hospital); Prof. Ulrich Ronellenfitsch, Prof. Jorg Kleeff, PD Dr. Johannes Klose, Dr Onur Bayram(University Hospital Halle (Saale)).

Greece: Dr Nikolaos Michalopoulos, Mr Theodoros Sidiropoulos, Ms Maria Papadoliopoulou, Prof. Nikolaos Arkadopoulos, Dr Nikolaos Danias, Dr Pantelis Vassiliu, Mr Panagiotis Kokoropoulos, Mr Maximos Frountzas, Dr Spyridon Christodoulou, Dr Efthimios Poulios(Attikon University Hospital); Dr Efstratios Kouskos, Dr Stavroula Papaeleftheriou, Dr Evdokia Romanou(General Hospital of Mytilene "Vostanio"); Dr Orestis Ioannidis, Ms Lydia Loutzidou, Ms Elissavet Anestiadou, Mr Stefanos Mpitsianis, Mr George Ntampakis, Mr Savvas Simeonidis, Dr Nikolaos Ouzounidis, Mr Konstantinos Zapsalis, Ms Aikaterini Karamitsou, Ms Ourania Kontaxi(General Hospital of Thessaloniki 'George Papanikolaou'); Mr Andreas Larentzakis, Dr Konstantinos Georgiou, Dr Gavriella-Zoi Vrakopoulou, Dr Alexandra Triantafyllou, Dr Tania TRIANTAFYLLOU(Hippocratio General Athens Hospital); Mr Alexandros Charalabopoulos, Ms Maria Boura, Ms Antonia Skotsimara, Mr Spyridon Davakis, Ms Efstratia Baili, Prof. Nikolaos Nikiteas, Mr Dimitrios Dimitroulis, Ms Zoe Garoufalia, Ms Eugenia Kotsifa, Mr Ifaistion Palios(Laiko Hospital Clinic); Prof. George Tzovaras, Prof. Ioannis Baloyiannis, Dr Konstantinos Perivoliotis(University Hospital of Larissa).

Ireland: Prof. Arnold Hill, Dr Gerard Sexton, Dr Angus Lloyd, Dr Ciara Maher, Dr James Toale, Dr Emily Lait, Dr Angela Canas-Martinez(Beaumont Hospital); Mr Muyiwa Aremu, Ms Brenda Murphy, Mr David Beddy, Dr Colum O'Reilly, Dr Megan Wilson(Connolly Hospital Blanchardstown); Dr Jim Byrne, Mr Mohammed Yasser Kayyal, Dr Amy Edwards Murphy, Mr Hamid Mustafa, Mr Muhammad Aakif, Dr Padraig Gardiner, Dr Ali Waris Syed, Dr Stephen O'Brien, Mr Mohammed Daoud(Cork University Hospital); Mr Sean Johnston, Mr Eabhard Mulligan, Dr Irele-Ifijeh Ahonkhai, Dr Sean Tee Lim, Mr Éanna J Ryan(Midlands Regional Hospital Tullamore); Ms Aisling M Hogan, Prof. Michael J Kerin, Dr Matthew G Davey, Dr Martin S Davey, Dr Dayna van de Hoef, Dr Ke En Oh(University Hospital Galway (UHG)); Prof. Peter Neary, Ms Tara Connelly, Helen Earley, Prof. Fiachra Cooke, Mr Peter McCullough, Ms Jessica Ryan, Mr Mohamed Alfatih Hamza(University Hospital Waterford).

Italy: Dr Nicoletta sveva Pipitone Federico, Dr Marcello Calabrò, Dr Andrea Muratore, Dr Alessandra Murgese, Dr Patrizia Marsanic, Dr Carlotta Bonasso, Dr Roberto Allocco(Agnelli Pinerolo Hospital); Dr Edoardo Segalini, Dr Marco Monti, Dr Domenico Giuliani, Dr Alberto Longo, Dr Gian Attilio Puerari, Dr Alessia Morello, Dr Ottavia Caserini(ASST Maggiore Hospital Crema); Dr Nicolò Tamini, Dr Claudia Stucchi, Dr Mauro Totis, Dr Luca Cigagna, Dr Massimo Oldani(ASST Monza, Ospedale San Gerardo); Dr Nicolò Maria Mariani, Dr Andrea Pisani Ceretti, Prof. Enrico Opocher, Dr Marco Giovenzana, Dr Vincenzo Nicastro, Dr Beatrice Giuliani(ASST Santi Paolo e Carlo); Prof. Donato F Altomare, Dr Arcangelo Picciariello, Dr Vincenzo Papagni, Dr Rigers Dibra, Dr Giuseppe Trigiante(Azienda Ospedaliero Universitaria Policlinico Bari); Dr Nicolò Fabbri, Prof. Carlo V. Feo, Dr Antonio Pesce, Dr Fioralba Pindozzi, Dr Silvia Gennari(Azienda Unità Sanitaria Locale di Ferrara, Chirurgia Generale); Dr Gabriella Teresa Capolupo, Prof. Caricato Marco, Dr Filippo Carannante, Dr Erica Mazzotta, Dr sara lauricella, Dr Gianluca Mascianà(Campus BIo Medico Hospital of Rome); Dr Valeria Tonini, Dr Maurizio Cervellera, Dr Egidio Milano, Dr Lodovico Sartarelli(Central Hospital of Taranto - SS. Annunziata); Mr Federico Ghignone, Prof. Giampaolo Ugolini, Mr Giovanni Taffurelli, Mr Davide Zattoni, Mr Isacco Montroni, Dr Letizia Santandrea, Mr Federico Mazzotti(Faenza Ospedale Degli Infermi); Prof. Gaetano Luglio, Prof. Giovanni Domenico De Palma, Dr Francesca Paola Tropeano, Dr Gianluca Pagano, Prof. Umberto Bracale, Dr Roberto Peltrini, Dr Maria Michela Di Nuzzo, Dr Nello Pirozzi, Prof. Francesco Corcione(Federico II University Hospital); Prof. Antonino Spinelli, Dr Caterina Foppa, Dr Annalisa Maroli, Dr Michele Carvello(Humanitas Research Hospital); Dr Raffaele Lombardi, Dr Michele Masetti, Dr Chiara Cipressi, Dr Giulia Ciabatti, Prof. Elio Jovine, Dr Matteo Rottoli, Dr Marta Tanzanu, Dr Angela Belvedere, Dr Anna Pezzuto, Dr Daniele Parlanti(IRCCS Azienda Ospedaliero Universitaria di Bologna, Bologna, Italy; Alma Mater Studiorum University of Bologna, Bologna, Italy (prev. Policlinico Sant'Orsola-Malpighi)); Dr Fabio Marino, Dr Fabrizio Perrone, Dr Giuseppe Lippolis, Dr Francesco Pezzolla(IRCCS 'Saverio de Bellis'); Dr Alessandro Michele Bonomi, Dr Michele Crespi, Dr Angelo Gabriele Epifani, Dr Alice Frontali(L. Sacco University Hospital); Mr Raffaele D'avino, Ms Ester Marra, Ms Roberta Abete(Naples Ospedale del mare); Dr Lucia Romano, Dr Andrea Nervini, Dr Francesco Maffione, Dr Antonio Giuliani(Ospedale Civile San Salvatore); Dr

Raffaele Porfidia, Prof. Sergio Grimaldi, Dr Maria Giovanna Ciolli, Dr Angela Romano, Dr Pietro Picarella(Ospedale Convenzionato Villa dei Fiori Acerra); Dr Giorgio Lisi, Dr Giulia Russo, Dr Leonardo Montemurro, Dr Giampaolo Galiffa, Prof. Massimo Carlini(Ospedale Sant'Eugenio); Dr Fausto Catena, Dr Gennaro Perrone, Dr Gabriele Petracca, Dr Elena Bonati, Prof. Paolo Del Rio, Dr Mario Giuffrida, Dr Lorenzo Pagliai(Parma University Hospital); Prof. Andrea Mingoli, Dr Pierfrancesco Lapolla, Dr Gioia Brachini, Dr Bruno Cirillo, Prof. Giorgio De Toma, Dr Immacolata Iannone, Dr Cristina De Padua, Dr Martina Zambon, Dr Simona Meneghini, Dr Pierfranco Maria Cicerchia, Prof. Paolo Sapienza(Policlinico Umberto I, Sapienza University of Rome); Mr Andrea Gattolin, Mr Roberto Rimonda, Ms Elisabetta Travaglio, Mr Francesco Riente, Ms Adriana Lena(Regina Montis Regalis Hospital); Dr Francesco Bianco, Dr Paola Incollingo, Sebastiano Grassia, Dr alessandra novi, Dr gregorio corcione(S. Leonardo Hospital - ASLNA3); Dr Daniele Sambucci, Dr Andrea Porta, Dr Massimiliano Coladonato, Dr Gabriele Soldini, Dr Francesca Scaltrini(Sacred Family Fatebenefratelli Hospital); Dr Diego Sasia, Dr Felice Borghi, Dr Maria Carmela Giuffrida, Dr Paola Ponzo, Dr Sara Salomone, Dr Marco Migliore, Dr Valentina Testa, Dr Alessandra Marano, Dr Daniele Simondi(Santa Croce and Carle Hospital); Dr Nicola Cillara, Dr Giaime Putzu, Dr Alessandro Cannavera, Dr Roberto Cardia(Santissima Trinità Hospital ATS Sardegna); Dr Arturo Roncone, Dr Matteo Papandrea, Dr Vincenzo Maiuri, Dr Giuseppe Bruzzese(Soverato Civil Hospital); Dr Alessandro Iacomino, Dr Ugo Grossi, Dr Giulio Aniello Santoro, Prof. Giacomo Zanus, Dr Nicola Passuello(Treviso Regional Hospital Cà Foncello); Dr Herald Nikaj, Prof. Sergio Gentilli, Dr Elisa Francone, Dr Elisa Reitano, Dr Giordana D'Aloisio(University Hospital Maggiore della Carità); Mr Gaetano Gallo, Ms Gilda De Paola, Prof. Giuseppina Vescio, Prof. Giuseppe Sammarco, Prof. Giuseppe Currò(University 'Magna Graecia' of Catanzaro); Prof. Salomone Di Saverio, Dr Sabrina Garbarino, Dr Maurizio Cannavo, Dr Nevio Menegat, Dr Alberto Reggiori, Dr Franco Pavesi, Dr Deborah Tornese, Dr Paolo Rocchi, Dr Carmelo Cotronea, Dr Ilenia Merlini (University of Insubria - Cittiglio Hospital and Surgeru 1 Varese); Edoardo Virgilio, Renato Costi, Filippo Montali, Alfredo Annicchiarico, Andrea Morini, Lorenzo Pagliai, Christan Franzini, Lorenzo Casali, Carlo Salvemini, Alessio Rollo(Vaio Hospital).

Jordan: Dr Hussam Nasser, Dr Majedah Hmeidan, Dr Mohammad Alawashreh(Al Hussain Hospital); Dr Anas Aljaiuossi, Dr Almu'atasim Khamees, Dr Luai Abu-Ismail, Mr Yousef A Yousef, Mr Ossaid Quneis, Mr Amer Abu Hmaid, Mr Mahmoud S Abwini, Dr Ahmad Riyad Othman, Mr Ali Guboug, Ms Dima Y Abu Ismail(Irbid Specialty Hospital); Dr SARI ALMI'ANI, Dr Nadeen Hijazin, Dr Farah Hammad, Dr Mirna Awbakh, Dr Karim Zaghloul, Dr Salameh Alarood, Dr AMRO ABULEIL, Dr Tahani Al-Hayek, Dr Ahmad Uraiqat, Dr Marleen Hijazin(King Hussein Medical Center); Prof. Raed Tayyem, Dr Ahmad Qasim, Dr Dema Abu mahfouz, Dr Zaid Alqadi, Dr Haneen Khalifa, Dr Majdi Alqudah, Dr Emad Doudin, Dr Abdallah Al daif alla, Dr Dina Hasan, Dr Duaa Abu nawas(New Zarqa Hospital ); Dr Fares Alma'aitah, Dr Hazim Ababneh, Dr Mohammad Buwaitel, Dr Malak Ababneh(Prince Hashim Bin Al Hussein Hospital).

Libya: Prof. Naser Alhasy, Ms Wafa Aldressi, Dr Akram Albarki, Ms Sarah Aldressi, Ms Soha Younis, Dr Sami Lawgaly(Benghazi Medical Center ); Dr Akram Alkaseek(Gharyan Central Hospital); Dr Fras Elhajdawe, Dr Reem Alkikle, Dr Reem Jamal Ghmagh, Dr Abdulqudus Deeknah, Dr Entisar Ahmed Ali Alshareea, Dr Eman Abdulwahed, Dr Rania Jobran, Dr MarwaIsa Biala(Tripoli Central Hospital); Dr Ahmed Msherghi, Dr Sanad Kanna(Tripoli University Hospital).

Malaysia: Dr Andee Dzulkarnaen Zakaria, Dr Zaidi Zakaria, Dr Muhammad Faeid Othman, Dr Jien Yen Soh, Dr Mohd Nizam Md Hashim, Dr Wan Mokhzani Wan Mokhter, Dr Michael Pak-Kai Wong, Dr Mohd Azem Fathi Mohammad Azmi, Dr Aimatnuddin Husairi Hussain, Dr Mohamad Fadli Mohd Yunus(Department of Surgery, School of Medical Sciences & Hospital USM, Universiti Sains Malaysia).

Mexico: Dr Noel Salgado-Nesme, Dr Paulina Moctezuma-Velázquez, Dr Juan Sainz-Hernández, Dr Francisco Alvarez-Bautista, Dr Hector Bravo-Avila, Dr Alejandro Hoyos-Torres(Instituto Nacional de Ciencias Medicas y Nutrición "Salvador Zubirán").

Namibia: Dr David W Borowski, Dr Kwasi Yeboah(Welwitschia Hospital).

New Zealand: Dr Deborah Wright, Dr Mostafa Amer, Dr Alex Hart(Dunedin Hospital).

Nigeria: Dr Ademola Adeyeye, Dr Elizabeth Enoch, Dr Akinola Akinmade, Dr Victor Kayode-Nissi, Dr Rebecca Adeyeye, Dr Samuel Larri(Afe Babalola University (ABUAD) Multi-system Hospital).

Palestinian Territory: Dr Raed Altiti, Dr Sarah Amro, Dr Shahd Al-Qasrawi, Raghad Abed-Allateef Lahlooh, Mohammad Smerat(Ahli Hospital); Dr lsam Awadallah, Dr Asmaa Anan, Ms Dina El-ashqar, Ms Wejdan Isleem, Ms Hala Abu Selmiyh, Mr Loay Kanou, Mr Belal Alhabil, Ms Noor Fannon, Ms Aseel Fannon, Dr Saed Owda(Al Shifaa Medical Complex); Dr Mohammed Aladini, Dr Iyad Ismail, Dr Bisan Ahmad, Dr Marah Musallam, Dr Mohmmad Mezead, Dr Safa Alaklook, Dr Marwa Alaklouk, Dr Mohammed Fetiha, Dr Anas Shehada, Dr Manal Alnuweiri(Al-Aqsa Martyrs Hospital).

Portugal: Mr José Pinto, Mr Fernando Valério, Mr Tiago Pavão(Centro Hospitalar Tondela-Viseu).

Russia: Prof. Andrey Koshel, Dr Evgeniy Drozdov(Siberian State Medical University).

Slovenia: Prof. Aleš Tomažič, Dr Jan Grosek, Dr Tajda Košir Božič, Dr Jurij Aleš Košir(University Medical Centre Ljubljana).

Spain: Dr Cristina Soto Montesinos, Dr Claudio Antonio Guariglia, Dr Meritxell Labró Ciurans, Dr Pablo Collera Ormazábal, Dr Lorena Sanchon Fructuoso, Dr Alexander Osorio Ramos, Dr Sara Pardo Lopez, Dr Roser Flores Clotet, Dr Carlos Javier Gómez Díaz, Dr Javier Curto López(Althaia Foundation Manresa Healthcare Network); Mr Ladislao Cayetano Paniagua, Ms Sara Pou Macayo(Consorci Sanitari de Terrassa - Hospital de Terrassa); Dra. Mercedes Estaire-Gómez, Dr Daniel Sánchez-Peláez, Dra. Elisa Jiménez-Higuera, Dr David Padilla-Valverde, Dr José Miguel Valverde-Mantecón, Dra. Rebeca Vitón-Herrero(Hospital General Universitario De Ciudad Real); Mr Renan Carlo Colombari, Ms María Fernandez Martínez, Dr Luis Miguel Jimenez-Gomez(Hospital General Universitario Gregorio Marañón); Mr Víctor Soria Aledo, Mr Jose Andrés Garcia Marin, Mr Francisco Ramos Soler, Ms Enrique Pellicer Franco, Ms Monica mengual-ballester, Ms Graciela valero Navarro, Dr Melody Baeza Murcia, Dr Lidia Betoret, Dr Sergio Annese, Dr Jose Luis Martinez-Moreno(Hospital General Universitario Morales Meseguer); Dr Virginia Jiménez Carneros, Dr Jose Luis Ramos Rodriguez, Dr Francisco Javier Jiménez Miramón, Ms Ainhoa Valle Rubio, Ms Jose Maria Jover Navalón, Ms Irene Herrero Muñoz, Ms Estefania Sanchez, Ms Aurea Tartas Ruiz(Hospital Universitario de Getafe); Mr Jorge Sancho-Muriel, Mr Pedro Rodriguez, Ms Ana Navio, Ms Hanna Cholewa, Mr Vicent Primo, Mr Matteo Frasson(Hospital Universitario La Fe, Valencia); Dr Alexander Forero-Torres, Prof. Ramon Cantero, Ms Beatriz Díaz San Andres, Ms Nuria Chavarria, Mr Fernando Prieto(Hospital Universitario La Paz); Dr Javier Martinez Alegre, Dr Francisca Lima Pinto, Ms Sara Nuñez O´Sullivan(Infanta Sofia University Hospital / Hospital Universitario Infanta Sofía); Dr Belén Matías-García, Dr Manuel Díez-Alonso, Dr Fernando Mendoza-Moreno, Dr Cristina Vera-Mansilla, Dr Ana Quiroga-Valcárcel, Dr Alma Blázquez-Martín, Dr Diego Córdova-García, Dr Pilar Hernández-Juara, Dr Enrique Ovejero-Merino, Dr Ana Sánchez-Gollarte(Principe de Asturias Hospital); Mr Pedro López Morales, Dr Jorge Alejandro Benavides Buleje, Dr Pedro Antonio Parra Baños, Ms María Ramírez Faraco, Mr Jose Manuel Muñoz Camarena, Dr Emilio Peña Ros, Ms María Milagros Carrasco Prats, Ms

Clara Giménez Francés, Mr Antonio Albarracín Marín-Blázquez(Reina Sofía General University Hospital); Mr José Andrés Cifuentes-Rodenas, Ms Marta Castro-Suárez, Ms Olga Claramonte-Bellmunt, Dr Enrique Colás-Ruiz, Ms Marta Tasende-Presedo(Son Llatzer University Hospital); Mr Alejandro Sánchez Arteaga, Mr Jose Tinoco González, Mr Daniel Díaz Gómez, Mr Luis Tallón Aguilar, Dr Virginia María Durán Muñoz-Cruzado, Ms Patricia García Muñoz, Mr Francisco Moreno Suero, Mr Joan Ricard Soler Frias, Mr Francisco Javier Espada Fuentes(The Virgen del Rocio University Hospital / Hospital Universitario Virgen del Rocío (HUVR)); Ms Alba Correa Bonito, Ms Ana Rodríguez Sánchez, Mr Carlos Cerdán Santacruz, Ms Elena Bermejo Marcos, Mr Francisco Eduardo Viamontes Ugalde, Mr Francisco Alberto Gimeno Calvo, Mr Jose María Lopesino González, Ms Livia Delgado Búrdalo, Mr Fernando Gijón Moya, Mr Javier García Septiem(Universitary Hospital of La Princesa); Prof. David Moro-Valdezate, Prof. Jose Martin-Arevalo, Dr Ana Izquierdo-Moreno, Dr Mireia Bauza-Collado, Dr Ricardo Gadea-Mateo, Prof. Alejandro Espi-Macias, Dr Leticia Perez-Santiago, Dr Ernesto Muñoz-Sornosa, Dr Sara Palomares-Casasus, Dr Ana Benitez-Riesco(University Clinic Hospital of Valencia); Mr Javier Rivera Castellano, Ms Ester Ramirez Caballero, Ms Beatriz Díaz Pérez, Mr Sergio González Hernández, Mr Samuel Morales Diaz, Ms Carlota Isabel Tuñon Fequánt, Mr Lazaro Javier Fernández López(University Hospital of the Canary Islands); Mr Luis Eduardo Pérez-Sánchez, Ms Ana María Feria-González, Ms María Savoie-Hontoria, Ms Cristina Vila-Zárate, Ms Jennifer García-Niebla(University Hospital of the Nuestra Senora de Candelaria / Hospital Universitario Ntra Sra de Candelaria); Prof. Beatriz De Andrés-Asenjo, Dr Jeancarlos Trujillo, Dr María Ruiz-Soriano, Dr Carlos Jezieniecki, Dr Tania Gómez-Sanz, Dr Guillermo Cabezudo, Prof. Juan Beltrán de Heredia(Valladolid University Clinical Hospital).

Sri Lanka: Dr Umesh Jayarajah, Dr Nagendiram Harivallavan, Dr Vitharanage Srimantha Dewsiri Rodrigo(Chilaw District General Hospital).

Sudan: Dr Mohmed Osman, Dr Ali Adil Ali karar(El-Rajhi Hospital); Mr Imad M. Bakheit, Dr Hytham K. S. Hamid, Dr Mohammed A. Babikir(Ibrahim Malik Teaching Hospital); Mr Mahmoud Saleh(Wad Madani Teaching Hospital).

Switzerland: Dr Christine Maurus, Dr Boumediene Guendil, Dr Ian Fournier, Carlos Alejandro Apestegui, Dr Ioannis Rotas, Dr Evangelos Kalogiannis, Dr Konstantinos Stratakis, Dr Ismael Turk, Dr Dimitri Chappaley(Hospital of Sion, Valais); Dr Marc-Olivier Sauvain, Dr Eleftherios Gialamas, Dr Aurelie Vuagniaux, Dr Michael Racine, Dr Roland Chautems, Dr Oliver Dwidar, Dr Xenofon Papazarkadas, Dr Pietro Ricciardi(Neuchâtel Hosptial); Dr Silvio Däster, Dr Savas D. Soysal, Dr Benjamin Wiesler, Dr Gabriel F. Hess, Dr Georg Henniger(University Hospital Basel); Prof. Matthias Turina, Dr Dominique Birrer, Dr Stephan Gerdes, Dr Karoline Horisberger(University Hospital Zurich / Universitatsspital Zurich).

Syria: Prof. Kusay Ayoub, Dr Aya Zazo, Dr Rama Zazo, Dr Baraa Shebli, Dr Ayham Alzahran, Dr Amr Hamza, Dr Zain Douba, Dr Mohamad Al Hashemi, Dr Elham Habash, Dr Roaa Rhayim(Aleppo University Hospital); Dr Mohamed Abdel-Maboud, Dr Mohamed Adwi, Dr Hasan Mostafa(Tishreen University Hospital).

Turkey: Dr Yuksel Altinel, Dr Merve Tokocin, Dr Serhat Meric, Dr Talar Aktokmakyan, Dr Fikret Calikoglu, Dr Tugba Calikoglu, Dr Cem Guneyli, Dr Onur Tokocin, Dr Salih Sevdi, Dr Yunus Aktimur(Bagcilar Research and Training Hospital); Dr Osman Bozbiyik, Prof. Tayfun Yoldas, Prof. Cemil Caliskan, Prof. Erhan Akgun, Prof. Mustafa Korkut, Dr Can Uc(Ege University Hospital); Prof. Sezai Leventoglu, Dr Can Sahin, Dr Emre Gulcek, Dr Safa Ozaydın, Dr Ali Yalcinkaya, Dr Begum Algul, Dr Kerim Karabulut, Dr Ahmet Faruk Oyanik, Dr Omer Batuhan Zor, Dr Berkay Enes Karaca(Gazi University Hospital); Dr Mehmet Eşref Ulutaş, Dr İsmail Hasırcı, Dr Yahya Alperen Bayraktar, Dr Gürcan Şimşek, Dr Kemal Arslan, Dr Ogün Erşen, Dr Alpaslan Şahin, Dr Ahmet Kılınç, Dr Sabri Özden, Dr Ersin Turan(Health Science University Konya City Hospital); Dr Yasin Kara, Dr Adem Özcan, Dr Mehmet Abdussamet Bozkurt, Dr Erkan Somuncu (Kanuni Sultan Süleyman Training and Research Hospital, Department of General Surgery); Prof. Emre Balık, Dr Emre Bozkurt, Dr Ibrahim Halil Özata, Dr Emre Özoran, Dr Tutku

Tüfekçi(Koc University Hospital); Dr Sertaç Ata Guler, Prof. Nihat Zafer Utkan, Dr Ozan Can Tatar(Kocaeli University Teaching Hospital); Dr Tevfik Kıvılcım uprak, Dr Aysegul Bahar Özocak, Dr Ahmet Akmercan, Dr Mümin Coşkun(Marmara university pendik research hospital ); Prof. Dr. Fatih Altintoprak, Dr Emre Gonullu, Dr Baris Mantoglu, Dr Zulfu Bayhan, Dr Recayi Capoglu, Dr Necatin Firat, Dr Emrah Akin, Dr Enis Dikicier, Dr Kayhan Ozdemir, Dr Tarik Harmantepe(Sakarya training and Research Hospital); Dr Elif Colak, Dr Huseyin Eraslan, Dr Engin Aybar, Dr Ahmet Can Sari, Dr Mustafa Safa Uyanik, Dr Suleyman Polat, Dr Ahmet Burak Ciftci, Dr Zehra Alan Koylu, Dr Mert Candan(Samsun Egitim ve Arastirma Hastanesi); Dr Değercan Yeşilyurt, Dr Gizem Kilinc Tuncer, Dr Gülberk Uslu, Dr Tayfun Kaya, Dr Semra Demirli Atici(University of Health Sciences Tepecik Training and Research Hospital); Dr Mert Tanal, Prof. Sitki Gurkan Yetkin, Prof. Mehmet Uludag, Dr Sinan Omeroglu, Prof. Bulent Citgez, Dr Cemal Kaya, Dr Onur Guven, Dr Aydin Eray Tufan, Dr Elif Baran, Dr Mehmet Ektiren(University of Health Sciences, Sisli Hamidiye Etfal Research and Training).

United Arab Emirates: Dr Amna Al-Wandi, Dr Haider Hamadi, Dr Abdulwahid Al-Wahedi, Dr Muzan Alkhaldi, Dr Heba Nofal(Al-Qassimi Hospital).

United Kingdom: Mr Heman Joshi, Mr James Ashcroft, Ms Victoria Hudson, Ms Siobhan Rooney, Mr Harry Kyriacou, Mr Ahmed Mostafa, Mr Henry Bennett, Ms Alexandra Entwistle-Thompson, Ms Rachel Hosking, Jean-Luc Duval, Mr Mohamed Rabie (Addenbrooke's Hospital); Mr Raimundas Lunevicius, Mr Robert P. Jones, Mr Andrew McAvoy, Mr Khalid Shahzad, Mr Mushfique Alam, Mr Zaid Al-Amiedy, Mr Arjun Kattakayam, Mr Matthew Sundhu, Mr Mirza Baig (Aintree University Hospital); Mr Richard Guy, Mr Mohamed Issa, Ms Dheepa Nair, Bryony David(Arrowe Park Hospital); Mr Pawan Mathur, Mr Maitham Alwhouhayb, Mr Asem Almaghrebi, Dr Armin Fardanesh(Barnet Hospital / The Royal Free London NHS Foundation Trust); Mr Jonathan Barker, Mr Mohammed Elniel, Dr Noor Al-Lamee, Dr Fatma Khan, Miss Emma Afify(Blackpool Teaching Hospitals NHS Foundation Trust); Mr Thomas Barnes, Mr Spyros Marinos(Buckinghamshire NHS Trust); Mr Yesar El-Dhuwaib, Louise Howe, Naomi Cruikshank, Aagat Sharma Khatiwada, Dr Hannah Knowles(Conquest Hospital); Ms Nichola Manu, Mr Sunanda Mahapatra, Mr Dale Vimalachandran, Mr Kunal Rajput, Mr Andrew Jones(Countess of Chester); Ms Julia Cheong, Mr Benjamin Stubbs, Mr George Neelankavil Davis, Dr Theodora Chatzmichail, Dr Mandeep Kang(Dorset County Hospital); Mr Patrick Collins, Mr Sharukh Sami, Mr Muneeb Zafar, Dr Niroshini Hemadasa(Dumfries and Galloway Royal Infirmary); Mr Ahmed El Zaafarany, Ms Vivian Ng, Mr Oliver Shihab, Ms Irena Stefanova, Mr Ahmed Tawfik(East Surrey Hospital); Dr Mhairi Clark, Mr Joseph Crozier, Mr Ben Douglass(Forth Valley Royal Hospital); Mr Shahrukh Ahmad, Mr Peter Sodde, Mr Joshua Boyes, Mr Siddhartha Handa, Ms Panna Patel(Furness General Hospital); Dr Rute Castelhano, Mr Jeffrey Lim, Dr Sherwin Ng, Dr Tatiana Garofalidou, Dr Alexis Adam(Great Western Hospital); Ms Katie Adams, Ms Sarah Wheatstone, Dr Andrew Yiu, Mr Imran Raza(Guy's and St Thomas' NHS Foundation Trust); Mr Chandrashekar Rangaiah, Ms Clare Adams, Mr Christos Antonios Kampourakis, Mr Iftikhar Mehmood(Harrogate District Hospital); Mr Thomas Chase, Mr Amir Ghanbari, Ms Irene Karderirinis, Mr Abdul Quddus, Ms Katerina Theodoropoulou, Dr Rafid Rahman, Aditya Borakati(Homerton Hospital); Mr Christopher Liao, Mr Thomas Curl-Roper, Ms Adeline Rankin, Mr Mazin Hamed, Mr Gakul Bhatta, Ms Monica Bogdan, Mr Subash Rai(James Paget University Hospital); Mr Giles Bond-smith, Ms Ola Shams, Ms Roshneen Ali, Dr Amanda Shabana, Dr Katherine Lee, Dr Rawan Ahmed, Dr David Fellows, Dr Mahul Patel(John Radcliffe Hospital); Dr Nityanandan Ganesan, Mr Dimitrios Kyriakidis, Dr Stephanie Obrowski, Ms Emila Paul, Ms Sian Jones, Ms Misti Ollier, Dr James Hackett, Mr Luke Dickerson, Mrs Sarah Rigby(Leighton Hospital); Mr Vivek Gupta, Mr Samuel Adegbola, Mr Seiver Karim, Mr Barnaby Farquharson, Dr Alex West, Dr Anna Lieberman, Dr Tamie Talab, Dr Ankit Sinha, Dr Zain Husain, Dr Jeffrey Chui Hom Lap(Lister Hospital); Miss Shantata Kudchadkar, Dr Harkiran Sagoo, Dr Saarah Ebrahim, Mr Jayesh Sagar(Luton & Dunstable Hospital); Dr Laith Alghazawi, Mr Mumtaz Bughio, Ms Farhat Amir, Mr Christopher J Smart, Mina Ragaa Fekry Abdelmalak(Macclesfield District General Hospital); Mr Yasser Abdul Aal, Mr Mohammed Khalil, Mr Aoff Khalil, Mr Mohammed Boushnaq, Mr Mohammed Hassan(Maidstone and Tunbridge Wells NHS Trust); Prof. James Hill, Mr Sean Magee(Manchester Royal Infirmary); Mr Niteen Tapuria, Mr Barrie Keeler, Mr Sidharth Kumar, Mr Sadhasivam Ramasamy, Mr Seshu Bylapudi(Milton Keynes University Hospital); Mr Min Maung, Ms Anne Macleod, Dr Emily Stokes(Monklands Hospital); Prof. Dean Harris, Mr Rhys Thomas, Dr Oliver Allon, Miss Emma Barlow(Morriston Hospital); Mr Thomas Badenoch(Musgrove Park Hospital); Ms Swati Bhasin, Ms Nuha Yassin, Jessica Chang, Zoe Gates, Saad Rehman, Rebecca Lefroy, Widad Ramadan(New Cross Hospital); Dr Varun Sarodaya, Mr Anthony Ramsanahie, Ms Artu Garg(Newham

University Hospital); Mr Jan Khan, Mr Dileep Kumar, Mr Christopher Payne(Ninewells Hospital); Mr Camilo Valero, Miss Christina Delimpalta, Mr Omar Khalil(Norfolk and Norwich University Hospital); Ms Katie Cross, Mr Mohamed Fakhrul-Aldeen(North Devon District Hospital); Ms Samreena Riaz, Mr Jamil Ahmed, Ms Vasilica Marcu, Dr Ionut Pop, Ebru Freed(Northampton General Hospital); Dr Evangeline Karamitsau, Mr Kamil Naidoo, Mr Josef Watfah, Dr Manahil Bashir(Northwick Park Hospital); Mr Ahmed Elsayed, Mr Janahan Sarveswaran, Dr Ayman Darwich, Mr Ahmed Ammar(Pinderfields General Hospital); Mr Farshid Ejtehadi, Ms Nazrin Assaf, Dr Emily Mills, Ibrahim Warrag, Ms Vardhini Vijay(Princess Alexandra Hospital); Ms Joanna Dudek, Mr Rizwan Ahmed, Mr Tarun Singhal, Dr Rodica Sorocovici, Dr Ahmed Mattar (Princess Royal University Hospital ); Mr Jim Khan, Ms Lesley-Ann Naik, Dr Charlotte Parfitt, Dr Soo Rin Park, Mr Samuel Stefan(Queen Alexandra Hospital, Portsmouth Hospitals NHS Trust); Mr Gary Nicholson, Miss Jessica Thompson, Miss Zahra Javid, Mr Ross McMahon, Dr Crystal Lee, Dr Mari-Claire McGuigan(Queen Elizabeth University Hospital Glasgow); Mr Hatim Albirnawi, Mr Anas Belhasan, Ms Rebecca Wookey, Bertie Marks(Queen Elizabeth University Hospital, Gateshead); Mr Pradeep Thomas, Ms Olatoyosi Williams, Mr prabhu Ravi, Dr Keren Pathmanathan, Mr Najam Husain(Queens Hospital Burton); Mr David Humes, Dr Christopher Lewis-Lloyd, Mr James Bailey, Mr Alfred Adiamah, Mr Alastair Morton, Dr Adil Rashid(Queen's Medical Centre); Prof. Susan Moug, Dr Ellen Groundwater, Miss Eleanor Massie(Royal Alexandra Hospital); Ms Meghana Taggarsi, Mr Louis Vitone, Mr Nick Heywood, Miss Lorraine Hickey, Dr Pawanda Phumiphakmethakul, Dr Cheerong Yip, Dr Sanjeev Pramanik, Mr Nauman Ahmed(Royal Blackburn Teaching Hospital); Ms Gemma Faulkner, Adam Rees, Henry Bilton(Royal Bolton Hospital); Mr Will Faux, Mr James Monteiro de Barros, 0 Thomas Lyons, Dr James Hughes(Royal Cornwall Hospital); Ms Urszula Donigiewicz, Ms Lisa Massey, Mr Frank D McDermott, Ms Niroshini Rajaretnam, Prof. Neil J Smart(Royal Devon and Exeter Hospital); Mr Gethin Williams, Mr Geraint Herbert, Ms Sapna Gupta, Ioan Davies(Royal Gwent Hospital); Dr Mandeep Kaur, Ms Victoria Pegna, Ms Natalie Sharma, Mr Christie Swamanatham(Royal Sussex County Hospital); Mr Akinfemi Akingboye, Mr Adewale Ayeni, Mr Muhammad Raheel(Russells Hall Hospital); Dr Thomas Hosfield, Mr Georgios Kyriakopoulos, Ms Lyndsay Pearce, Dr Michal Woyton, Dr Zak Shehata, Mourtada Abakar(Salford Royal Hospital); Ms Victoria Morrison-Jones, Mr Dylan Green, Jamie Mawhinney, Jack Broadbent, Charlene Khoza(Salisbury District Hospital); Mr Mohamed Albendary, Mr Rajeev Peravali, Mr Pratik Bhattacharya, Mr Ali Yasen Y Mohamedahmed, Mr Shafquat Zaman(Sandwell General Hospital); Miss Elisa Lenzi, Miss Dimple Sapre, Miss Francesca Malcolm, Mr Krishnamurthy Badrinath(Sherwood Forest Hospitals NHS Foundation Trust); Mr Angus McNair, Mr Samuel Lawday, Dr Hugo Cohen, Dr Tarik Jichi, Dr Jessica Kennett, Mr Ben Patel, Mr Kit Lam, Dr Ryan Nolan, Dr Zoe Bakewell(Southmead Hospital Bristol); Mr Paul Ainsworth, Mr Aloka Suwanna Danwaththa Liyanage, Bianca Wadham, Virginia Ledda(Southport & Ormskirk Hospital); Mr Alex Chung, Ms Katherine Williams, Ms Sarah Thoukididou, Ms Alice Baggaley, Mr James Dale, Mr Tom Moore(St George's Hospital); Dr Damian Broadhurst, Mr Quasim Humayun, Ms Carla Rengifo, Mr Omar Lasheen, Mr Michael Flatman(Stepping Hill Hospital); Mr Mohamed Thaha, Mr Tobi Ayorinde, Dr Jyoti Salhan(The Royal London Hospital); Mr Chetan Parmar, Dr Emre Doganay, Dr Alexandra Sharpe, Dr Emily Thomas-Williams(The Whittington Hospital); David Naumann, James Halle-Smith, Arlo Whitehouse, Prof. Thomas Pinkney(University Hospital Birmingham); Mr Michael Helley, Ms Hoey Koh, Ms Emma Howie, Ms Gillian McColl, Ms Abigail Hayward, Dr Zara Bell(University Hospital Crosshouse ); Mr James Horwood, Nicola Reeves, Michael Shinkwin, Prof. Jared Torkington, Louise Dasilva, Athanasios Karategos(University Hospital of Wales); Mr Segun Komolafe, Ms Anna Bleakley, Dr Samantha Ng(University Hospital Wishaw); Mr Jamshed Shabbir, Mr Thomas Maccabe, Ms Sophie Rozwadowski, Dr Ryan Preece, Dr Abigail Campbell(University Hospitals Bristol / Bristol Royal Infirmary); Mr Sebastian Smolarek, Ms Supriya Balasubramanya, Mr Joshua Franklyn, Dr Shivanie Acharya, Dr Aye Chan-Thu, Dr Joseph Hearle, Dr Lucy Yao, Dr Nicolas Li, Dr Emily Ko, Dr Laurel Tuckey, Dr Eliot Leonard, Dr Pooja Dhavala(University Hospitals Plymouth NHS Trust); James Wyatt, Mr Nagarajan Pranesh, Ms Fran Oldfield, Caroline Louise English, Anna McClune, Jessica Banks, Mr Omar Mahmoud, Mr Iain Blake(Warrington Hospital); Ms Farhat VN Din, Mr Peter G Vaughan-Shaw, Mr Nicholas T Ventham, Ms Marion MacRury, Mr Louis F Buijs, Ms Olivia Pestrin, Dr Jennifer Zhang, Dr Polly Chapman, Dr Dean McAvoy, Dr Jessica McClintick, Mr Alessandro Sgrò(Western General Hospital); Mr Michael Chadwick, Ms Eva Vitovska, Miss Rachael Clifford, Dr Kunal Rajput, Dr Lara Jane Rimmer(Whiston Hospital); Mr Abozed Ben-Sassi, Mr Osama Elkomy(Wrexham Maelor Hospital); Mr Marius Paraoan, Mr Suraj Math, Mr Santhosh Loganathan(Wrightington, Wigan and Leigh Teaching Hospitals NHS Foundation Trust); Ms Sarah Duff, Ms Sharon Storey, Ms Rachael Hine, Mr Paras Batra, Ms Meera Patel, Mr Azam Khan, Mr Ben Brown, Mutee Rehman, Clare Bonner, Elmuiz Hsabo(Wythenshawe Hospital); Mr Michael Lim, Ms Suzie Green, Mr Henry Watson, Mr Pavlos Iosifidis, Mr Hossam Samy, Mr Simon East, Mr Richard Bond(York Teaching Hospital).

United States of America: Dr Yesenia Rojas-Khalil, Dr Hector Garcia-Chavez, Dr Atif Iqbal(Baylor College of Medicine); Dr Guy Orangio, Dr Kurt Davis, Dr Luv Hajirawala, Ms Elyse Bevier-Rawls(Louisiana State University Health Sciences Center); Dr Patricia Sylla, Ms Gurpawan Kang, Ms Deepika Bhasin (Mount Sinai Hospital); Dr Farah Monzur, Dr Mohammed Al-Sadawi, Dr Joseph Mizrahi, Dr Adam Myer, Dr Leslie Klyachman, (Stony Brook Medicine); Dr Elizabeth Raskin, Dr Tatyana Polyak, Dr Deborah S Keller(University of California at Davis Medical Center); Dr Vlad V. Simianu, Dr Celine R. Soriano, Dr Meredith P. Johnson (Virginia Mason Medical Center).

Yemen: Dr Shada Alemad, Dr Bushra alshaikh, Ala'a Adel Jabr Bin Jabr, Manal Jamal Al-dhaheri, Mohammed Mohammed Al-Shehari(Alkhamseen hospital); Dr Fatima Al-Eryani, Dr Nashwan Tashan, Dr Rudaina Albakry(Alkuwait University Hospital); Prof. Waheeb Al-Kubati, Dr Khadega Abotaleb, Dr Ashwaq Ziyad, Ghadeer Al-wajeeh, Ghofran Al-nahwi, Mohammed Sabbar(Alwesam International Hospital).

## SUPPLEMENTARY FIGURES & TABLES

### Supplementary Table 1: Consent waiver by country

| Country | Consent waived | Approving body |
|---|---|---|
| Argentina | Yes | National government approval |
| Australia | No | |
| Austria | No | |
| Azerbaijan | No | |
| Belgium | No | |
| Canada | Yes | Local institute ethical board |
| Columbia | No | |
| Denmark | No | |
| Egypt | No | |
| Finland | Yes | Local institute ethics |
| Germany* | Yes | Local institutional ethical |
| Greece | No | |
| Ireland | Yes | Local research and audit committee |
| Italy | No | |
| Jordan | Yes | |
| Libya | No | |
| Malaysia | No | |
| Mexico | No | |
| Namibia | Yes | Local institute ethics |
| New Zealand | No | |
| Nigeria | Yes | Local institute ethics |
| Palestine Territory | No | |
| Portugal | No | |
| Russia | No | |
| Slovenia | No | |
| Spain | No | |
| Sri Lanka | Yes | Institutional ethics board |
| Sudan | Yes | Local institute ethics |
| Switzerland | No | |
| Syria | Yes | |
| Turkey | No | |
| United Arab Emirates | No | |
| United Kingdon | Yes | Health Research Authority |
| United States of America | No | |
| Yemen | Yes | |

## Supplementary Table 2: Surgical treatment by geographical region

| Characteristic | **Australasia**, N = 29[1] | **North America**, N = 27[1] | **Rest of Europe**, N = 311[1] | **Rest of the world**, N = 59[1] | **UK**, N = 356[1] |
|---|---|---|---|---|---|
| ***Indication for surgery*** | | | | | |
| Peritonitis | 12 (41.4%) | 14 (53.8%) | 182 (58.5%) | 39 (67.2%) | 238 (66.9%) |
| Failed conservative treatment | 17 (58.6%) | 9 (34.6%) | 104 (33.4%) | 9 (15.5%) | 105 (29.5%) |
| Fistula | 0 (0.0%) | 3 (11.5%) | 20 (6.4%) | 2 (3.4%) | 13 (3.7%) |
| Bleeding | 0 (0.0%) | 0 (0.0%) | 5 (1.6%) | 8 (13.8%) | 0 (0.0%) |
| (Missing) Or NA | 0 | 1 | 0 | 1 | 0 |
| **How was acute diverticulitis diagnosed?** | | | | | |
| Via multiplanar CT | 23 (79.3%) | 25 (92.6%) | 288 (92.6%) | 23 (39.0%) | 328 (92.1%) |
| During emergency surgery | 6 (20.7%) | 2 (7.4%) | 23 (7.4%) | 36 (61.0%) | 28 (7.9%) |
| **Hinchey score** | | | | | |
| Hinchey Ia | 5 (19.2%) | 3 (11.5%) | 47 (17.7%) | 6 (12.8%) | 65 (19.7%) |
| Hinchey Ib | 2 (7.7%) | 3 (11.5%) | 27 (10.2%) | 1 (2.1%) | 25 (7.6%) |
| Hinchey II | 2 (7.7%) | 3 (11.5%) | 21 (7.9%) | 4 (8.5%) | 20 (6.1%) |
| Hinchey III | 11 (42.3%) | 8 (30.8%) | 130 (49.1%) | 27 (57.4%) | 143 (43.3%) |
| Hinchey IV | 6 (23.1%) | 9 (34.6%) | 40 (15.1%) | 9 (19.1%) | 77 (23.3%) |
| Missing | 3 | 1 | 46 | 12 | 26 |
| **Pre-operative CRP (mg/L)** | | | | | |
| < 2 mg/L | 1 (3.6%) | 0 (0.0%) | 8 (2.8%) | 1 (3.7%) | 6 (1.7%) |
| 2 to 100 mg/L | 14 (50.0%) | 7 (53.8%) | 102 (35.7%) | 8 (29.6%) | 100 (28.6%) |
| >100 to 200 mg/L | 4 (14.3%) | 3 (23.1%) | 82 (28.7%) | 4 (14.8%) | 84 (24.0%) |
| > 200 mg/L | 9 (32.1%) | 3 (23.1%) | 94 (32.9%) | 14 (51.9%) | 160 (45.7%) |
| Missing | 1 | 14 | 25 | 32 | 6 |
| **Q-SOFA score** | | | | | |
| 0 | 19 (65.5%) | 21 (77.8%) | 249 (80.1%) | 25 (42.4%) | 276 (77.5%) |
| 1 | 9 (31.0%) | 5 (18.5%) | 50 (16.1%) | 23 (39.0%) | 61 (17.1%) |
| 2 | 1 (3.4%) | 1 (3.7%) | 8 (2.6%) | 5 (8.5%) | 12 (3.4%) |
| 3 | 0 (0.0%) | 0 (0.0%) | 4 (1.3%) | 6 (10.2%) | 7 (2.0%) |
| ***Free fluid*** | | | | | |
| No | 9 (31.0%) | 10 (37.0%) | 107 (34.4%) | 15 (25.9%) | 120 (33.8%) |
| Yes | 20 (69.0%) | 17 (63.0%) | 204 (65.6%) | 43 (74.1%) | 235 (66.2%) |
| (Missing) Or NA | 0 | 0 | 0 | 1 | 1 |
| ***If free fluid*** | | | | | |
| Localised fluid | 9 (45.0%) | 4 (23.5%) | 108 (52.9%) | 21 (48.8%) | 108 (46.0%) |
| Generalised | 11 (55.0%) | 13 (76.5%) | 96 (47.1%) | 22 (51.2%) | 127 (54.0%) |
| ***Free fluid type*** | | | | | |
| Haemosanginous | 3 (15.0%) | 0 (0.0%) | 34 (16.7%) | 7 (16.3%) | 15 (6.4%) |
| Purulent | 11 (55.0%) | 8 (47.1%) | 130 (63.7%) | 27 (62.8%) | 143 (60.9%) |
| Faeculant | 6 (30.0%) | 9 (52.9%) | 40 (19.6%) | 9 (20.9%) | 77 (32.8%) |
| ***Perforation*** | | | | | |
| No | 8 (27.6%) | 5 (18.5%) | 116 (37.3%) | 19 (32.8%) | 96 (27.0%) |
| Yes | 21 (72.4%) | 22 (81.5%) | 195 (62.7%) | 39 (67.2%) | 260 (73.0%) |
| (Missing) Or NA | 0 | 0 | 0 | 1 | 0 |
| ***Fibrinous adhesions*** | | | | | |
| No | 16 (55.2%) | 13 (48.1%) | 116 (37.3%) | 19 (32.8%) | 160 (44.9%) |
| Yes | 13 (44.8%) | 14 (51.9%) | 195 (62.7%) | 39 (67.2%) | 196 (55.1%) |
| (Missing) Or NA | 0 | 0 | 0 | 1 | 0 |
| ***Interloop abscesses*** | | | | | |
| No | 24 (82.8%) | 9 (33.3%) | 246 (79.1%) | 40 (69.0%) | 247 (69.4%) |
| Yes | 5 (17.2%) | 18 (66.7%) | 65 (20.9%) | 18 (31.0%) | 109 (30.6%) |
| (Missing) Or NA | 0 | 0 | 0 | 1 | 0 |
| ***Subphrenic abscesses*** | | | | | |
| No | 29 (100.0%) | 26 (96.3%) | 302 (97.1%) | 47 (81.0%) | 329 (92.7%) |
| Yes | 0 (0.0%) | 1 (3.7%) | 9 (2.9%) | 11 (19.0%) | 26 (7.3%) |
| (Missing) Or NA | 0 | 0 | 0 | 1 | 1 |
| ***Fistula*** | | | | | |

| | | | | | |
|---|---|---|---|---|---|
| No | 28 (96.6%) | 24 (88.9%) | 287 (92.3%) | 55 (94.8%) | 335 (94.1%) |
| Yes | 1 (3.4%) | 3 (11.1%) | 24 (7.7%) | 3 (5.2%) | 21 (5.9%) |
| (Missing) Or NA | 0 | 0 | 0 | 1 | 0 |
| ***Surgical approach*** | | | | | |
| Open | 21 (72.4%) | 10 (37.0%) | 168 (54.0%) | 42 (72.4%) | 292 (82.0%) |
| Laparoscopic | 4 (13.8%) | 12 (44.4%) | 114 (36.7%) | 10 (17.2%) | 48 (13.5%) |
| Robotic | 0 (0.0%) | 0 (0.0%) | 1 (0.3%) | 0 (0.0%) | 0 (0.0%) |
| Laparoscopic / Robotic - converted to open | 4 (13.8%) | 5 (18.5%) | 28 (9.0%) | 6 (10.3%) | 16 (4.5%) |
| (Missing) Or NA | 0 | 0 | 0 | 1 | 0 |
| ***Lavage only*** | | | | | |
| No | 28 (96.6%) | 25 (92.6%) | 289 (92.9%) | 46 (79.3%) | 304 (85.4%) |
| Yes | 1 (3.4%) | 2 (7.4%) | 22 (7.1%) | 12 (20.7%) | 52 (14.6%) |
| (Missing) Or NA | 0 | 0 | 0 | 1 | 0 |
| ***Surgical drain*** | | | | | |
| No | 11 (37.9%) | 13 (48.1%) | 165 (53.1%) | 10 (17.2%) | 94 (26.4%) |
| Yes | 18 (62.1%) | 14 (51.9%) | 146 (46.9%) | 48 (82.8%) | 262 (73.6%) |
| (Missing) Or NA | 0 | 0 | 0 | 1 | 0 |
| ***Resection*** | | | | | |
| No | 2 (6.9%) | 4 (14.8%) | 28 (9.0%) | 20 (34.5%) | 52 (14.6%) |
| Yes | 27 (93.1%) | 23 (85.2%) | 283 (91.0%) | 38 (65.5%) | 304 (85.4%) |
| (Missing) Or NA | 0 | 0 | 0 | 1 | 0 |
| ***Primary anastomosis*** | ***n=27*** | ***n=23*** | ***n=283*** | ***n=38*** | ***n=304*** |
| No | 24 (89.9%) | 11(47.7%) | 153 (54.1%) | 26 (68.4%) | 281 (92.4%) |
| Yes | 3 (11.1%) | 12 (52.3%) | 130 (45.9%) | 12 (31.6%) | 23 (7.6%) |
| ***Stoma formation*** | | | | | |
| End colostomy | 23 (92.0%) | 11 (64.7%) | 143 (86.1%) | 18 (69.2%) | 248 (91.9%) |
| End ileostomy | 0 (0.0%) | 0 (0.0%) | 0 (0.0%) | 3 (11.5%) | 4 (1.5%) |
| Loop colostomy | 0 (0.0%) | 0 (0.0%) | 4 (2.4%) | 4 (15.4%) | 6 (2.2%) |
| Loop ileostomy | 2 (8.0%) | 5 (29.4%) | 19 (11.4%) | 1 (3.8%) | 11 (4.1%) |
| Diverting / Abcarian (blowhole) loop stoma | 0 (0.0%) | 1 (5.9%) | 0 (0.0%) | 0 (0.0%) | 1 (0.4%) |
| ***Post op complications (30-day)*** | | | | | |
| None | 10 (35.7%) | 15 (75.0%) | 197 (63.5%) | 33 (56.9%) | 203 (59.2%) |
| One | 12 (42.9%) | 4 (20.0%) | 74 (23.9%) | 16 (27.6%) | 105 (30.6%) |
| More than one | 6 (21.4%) | 1 (5.0%) | 39 (12.6%) | 9 (15.5%) | 35 (10.2%) |
| Missing | 1 | 7 | 1 | 1 | 13 |
| ***Clavien Dindo grade of worst complication (30-day)*** | | | | | |
| Minor (I-II) | 12/18 (66%) | 3/5 (60%) | 60/113 (53%) | 12/25 (48%) | 84/140 60%) |
| Major (III-V) | 6/18 (34%) | 2/5(40%) | 53 /113(47%) | 13/25 (52%) | 56/140 (40%) |

[1]n (%)

**Abbreviations:** BMI: body mass index, CT: computed tomography, Q-SOFA: quick Sepsis Related Organ Failure Assessment

**Supplementary Table 3: Countries included in the matched cohort analysis**

| **Characteristic** | **HIC** N = 1,005[1] | **LMIC** N = 335[1] |
|---|---|---|
| Country | | |
| Argentina | 2 (0.2%) | 0 (0.0%) |
| Australia | 43 (4.3%) | 0 (0.0%) |
| Austria | 8 (0.8%) | 0 (0.0%) |
| Azerbaijan | 0 (0.0%) | 9 (2.7%) |
| Belgium | 14 (1.4%) | 0 (0.0%) |
| Canada | 1 (0.1%) | 0 (0.0%) |
| Colombia | 0 (0.0%) | 27 (8.1%) |
| Denmark | 1 (0.1%) | 0 (0.0%) |
| Egypt | 0 (0.0%) | 49 (14.6%) |
| Finland | 23 (2.3%) | 0 (0.0%) |
| Germany | 5 (0.5%) | 0 (0.0%) |
| Greece | 9 (0.9%) | 0 (0.0%) |
| Ireland | 29 (2.9%) | 0 (0.0%) |
| Italy | 88 (8.8%) | 0 (0.0%) |
| Jordan | 0 (0.0%) | 25 (7.5%) |
| Libya | 0 (0.0%) | 15 (4.5%) |
| Malaysia | 0 (0.0%) | 6 (1.8%) |
| Mexico | 0 (0.0%) | 10 (3.0%) |
| Namibia | 0 (0.0%) | 8 (2.4%) |
| New Zealand | 1 (0.1%) | 0 (0.0%) |
| Nigeria | 0 (0.0%) | 4 (1.2%) |
| Palestinian Territory, Occupied | 0 (0.0%) | 5 (1.5%) |
| Russian Federation | 0 (0.0%) | 6 (1.8%) |
| Slovenia | 3 (0.3%) | 0 (0.0%) |
| Spain | 113 (11.2%) | 0 (0.0%) |
| Sri Lanka | 0 (0.0%) | 1 (0.3%) |
| Sudan | 0 (0.0%) | 6 (1.8%) |
| Switzerland | 27 (2.7%) | 0 (0.0%) |
| Syria | 0 (0.0%) | 4 (1.2%) |
| Turkey | 0 (0.0%) | 152 (45.4%) |
| United Kingdom | 579 (57.6%) | 0 (0.0%) |
| United States of America | 59 (5.9%) | 0 (0.0%) |
| Yemen | 0 (0.0%) | 8 (2.4%) |

[1]n (%)

**Supplementary Tables 4A and 4B: Pre and propensity score matching in the matched cohort analysis**

| Pre matching | level | HIC | LMIC | p-value | SMD |
|---|---|---|---|---|---|
| N | | 5544 | 335 | | |
| Age (mean (SD)) | | 60.59 (14.86) | 56.21 (15.51) | <0.001 | 0.288 |
| Sex (%) | Male | 2504 (45.2) | 199 (59.4) | <0.001 | 0.288 |
| | Female | 3040 (54.8) | 136 (40.6) | | |
| BMI (mean (SD)) | | 31.24 (26.18) | 35.86 (42.26) | 0.003 | 0.131 |
| Smoking status (%) | Never | 3139 (56.6) | 158 (47.2) | <0.001 | 0.294 |
| | Current | 1123 (20.3) | 111 (33.1) | | |
| | Ex-smoker | 1282 (23.1) | 66 (19.7) | | |

| Post matching | level | HIC | LMIC | p-value | SMD |
|---|---|---|---|---|---|
| N | | 1005 | 335 | | |
| Age (mean (SD)) | | 57.00 (14.67) | 56.21 (15.51) | 0.401 | 0.052 |
| Sex (%) | Male | 596 (59.3) | 199 (59.4) | 1.000 | 0.002 |
| | Female | 409 (40.7) | 136 (40.6) | | |
| BMI (mean (SD)) | | 37.64 (48.14) | 35.86 (42.26) | 0.546 | 0.039 |
| Smoking status (%) | Never | 481 (47.9) | 158 (47.2) | 0.935 | 0.023 |
| | Current | 335 (33.3) | 111 (33.1) | | |
| | Ex-smoker | 189 (18.8) | 66 (19.7) | | |

**Abbreviations:** BMI: body mass index, HIC: High income countries, LMIC: low-middle income countries, SD: standard deviation, SMD: standardised mean difference

**Supplementary Table 5: Indications and procedures undertaken in those patients undergoing surgical intervention for the whole cohort**

| **Characteristic** | **N = 782**[1] |
|---|---|
| ***Indication for surgery*** | |
| Peritonitis | 485 (62.2%) |
| Failed conservative treatment | 244 (31.3%) |
| Fistula | 38 (4.9%) |
| Bleeding | 13 (1.7%) |
| (Missing) Or NA | 2 |
| **How was acute diverticulitis diagnosed?** | |
| Via multiplanar CT | 687 (87.9%) |
| During emergency surgery | 95 (12.1%) |
| **Hinchey score** | |
| Hinchey Ia | 126 (18.2%) |
| Hinchey Ib | 58 (8.4%) |
| Hinchey II | 50 (7.2%) |
| Hinchey III | 319 (46.0%) |
| Hinchey IV | 141 (20.3%) |
| Missing or N/A | 88 |
| **C-reactive protein test (CRP), mg/L** | |
| < 2 mg/L | 16 (2.3%) |
| 2 to 100 mg/L | 231 (32.8%) |
| >100 to 200 mg/L | 177 (25.1%) |
| > 200 mg/L | 280 (39.8%) |
| Missing | 68 |
| **Q sofa score** | |
| 0 | 590 (75.4%) |
| 1 | 148 (18.9%) |
| 2 | 27 (3.5%) |

| | |
|---|---|
| 3 | 17 (2.2%) |
| ***Free fluid*** | |
| No | 261 (33.5%) |
| Yes | 519 (66.5%) |
| (Missing) Or NA | 2 |
| ***If free fluid n=519*** | |
| Localised fluid | 250 (48.2%) |
| Generalised | 269 (51.8%) |
| ***Free fluid type n=519*** | |
| Haemosanginous | 59 (11.4%) |
| Purulent | 319 (61.5%) |
| Faeculant | 141 (27.2%) |
| ***Perforation*** | |
| No | 244 (31.2%) |
| Yes | 537 (68.8%) |
| (Missing) Or NA | 1 |
| ***Fibrinous adhesions*** | |
| No | 324 (41.5%) |
| Yes | 457 (58.5%) |
| (Missing) Or NA | 1 |
| ***Interloop abscesses*** | |
| No | 566 (72.5%) |
| Yes | 215 (27.5%) |
| (Missing) Or NA | 1 |
| ***Subphrenic abscesses*** | |
| No | 733 (94.0%) |
| Yes | 47 (6.0%) |
| (Missing) Or NA | 2 |
| ***Fistula*** | |
| No | 729 (93.3%) |
| Yes | 52 (6.7%) |
| (Missing) Or NA | 1 |
| ***Surgical approach*** | |
| Open | 533 (68.2%) |
| Laparoscopic | 188 (24.1%) |
| Robotic | 1 (0.1%) |
| Laparoscopic / Robotic - converted to open | 59 (7.6%) |
| (Missing) Or NA | 1 |
| ***Lavage only*** | |
| No | 692 (88.6%) |
| Yes | 89 (11.4%) |
| (Missing) Or NA | 1 |
| ***Surgical drain*** | |
| No | 293 (37.5%) |
| Yes | 488 (62.5%) |
| (Missing) Or NA | 1 |
| ***Resection*** | |
| No | 106 (13.6%) |
| Yes | 675 (86.4%) |
| (Missing) Or NA | 1 |
| ***Primary anastomosis if resection n=675*** | |
| No | 495 (73.3%) |
| Yes | 180 (26.6%) |
| ***Stoma formation (end or covering) n=504*** | |
| End colostomy | 443 (87.9%) |
| End ileostomy | 7 (1.4%) |
| Loop colostomy | 14 (2.8%) |
| Loop ileostomy | 38 (7.5%) |
| Diverting / Abcarian (blowhole) loop stoma | 2 (0.4%) |
| ***Post operative complications (30-day)*** | |
| None | 458 (60.3%) |
| One | 211 (27.8%) |
| More than one | 90 (11.9%) |
| Missing | 23 |
| ***Clavien Dindo grade of worst complications (30-day)*** | |
| Minor (I-II) | 171/301 (56.9%) |

| | |
|---|---|
| Major (III-IV) | 130/301 (43.1%) |

[1]n (%)

**Abbreviations:** BMI: body mass index, CRP: c-reactive protein, CT: computed tomography, Q-SOFA: quick Sepsis Related Organ Failure Assessment

**Supplementary Table 6 Uncomplicated disease ambulatory vs admitted: patient characteristics**

| **Characteristic** | **Ambulatory,** N = 849[1] | **Inpatient,** N = 1,949[1] | **Overall,** N=2798[1] | **p-value**[2] |
|---|---|---|---|---|
| **Ethnicity** | | | | |
| White | 715 (89.4%) | 1,768 (91.4%) | 2,483 (90.8%) | 0.002 |
| Mixed / Multiple ethnic groups | 21 (2.6%) | 61 (3.2%) | 82 (3.0%) | |
| Asian / Asian British | 15 (1.9%) | 42 (2.2%) | 57 (2.1%) | |
| Black / African / Caribbean / Black African | 18 (2.3%) | 13 (0.7%) | 31 (1.1%) | |
| Other ethnic group | 31 (3.9%) | 50 (2.6%) | 81 (3.0%) | |
| (Missing) Or NA | 49 | 15 | 64 | |
| **Sex** | | | | |
| Male | 379 (44.6%) | 806 (41.4%) | 1,185 (42.4%) | 0.11 |
| Female | 470 (55.4%) | 1,143 (58.6%) | 1,613 (57.6%) | |
| **Smoking status** | | | | |
| Never | 533 (63.2%) | 1,087 (56.2%) | 1,620 (58.4%) | <0.001 |
| Current (within the last six weeks) | 161 (19.1%) | 380 (19.7%) | 541 (19.5%) | |
| Ex-smoker | 149 (17.7%) | 466 (24.1%) | 615 (22.2%) | |
| (Missing) Or NA | 6 | 16 | 22 | |
| **Age** | | | | |
| Age (years) | 58 (13) | 61 (15) | 60 (15) | <0.001 |
| **BMI** | | | | |
| Median (IQR) | 27.8 (24.8, 31.6) | 28.2 (24.9, 32.7) | 28.0 (24.8, 32.2) | 0.078 |
| (Missing) Or NA | 113 | 63 | 176 | |
| **Charlson score** | | | | |
| None | 200 (23.6%) | 409 (21.0%) | 609 (21.8%) | <0.001 |
| Mild | 373 (43.9%) | 705 (36.2%) | 1,078 (38.5%) | |
| Moderate | 195 (23.0%) | 469 (24.1%) | 664 (23.7%) | |
| Severe | 81 (9.5%) | 366 (18.8%) | 447 (16.0%) | |

[1]n (%); Median (IQR) [2]Fisher's exact test; Pearson's Chi-squared test

**Abbreviations:** BMI: body mass index, IQR: inter-quartile range

**Supplementary Table 7: Overall 30-day outcomes in the matched cohort analysis**

| All patients | HIC n=1005 | LMIC n=335 |
|---|---|---|
| **What was the outcome of the initial treatment (for ambulatory/outpatients)** | **N=190** | **N=59** |
| Failure | 23 (12.6%) | 6 (11.1%) |
| Successful | 160 (87.4%) | 48 (88.9%) |
| (Missing) Or NA | 7 | 5 |
| **What was the outcome of the initial treatment (for inpatients)** | **N=815** | **N=276** |
| Failure | 87 (10.9%) | 33 (12.4%) |
| Successful | 710 (89.1%) | 233 (87.6%) |
| Missing Or NA | 18 | 10 |
| **Was the patient still alive at the time of the 30-day follow-up?** | **N=1005** | **N=335** |
| No | 20 (2.1%) | 20 (6.2%) |
| Yes | 949 (97.9%) | 304 (93.8%) |
| (Missing) Or NA | 36 | 10 |
| **If NO, was death related to diverticulitis** | **N=16** | **N=20** |
| Yes | 13 (65.0%) | 10 (50%) |
| No | 5 (25.0%) | 10 (50%) |
| Not known | 2 (10.0%) | 0 (0%) |

[1]n (%)

**Supplementary Figure 1: Patient flow and reasons for exclusion**

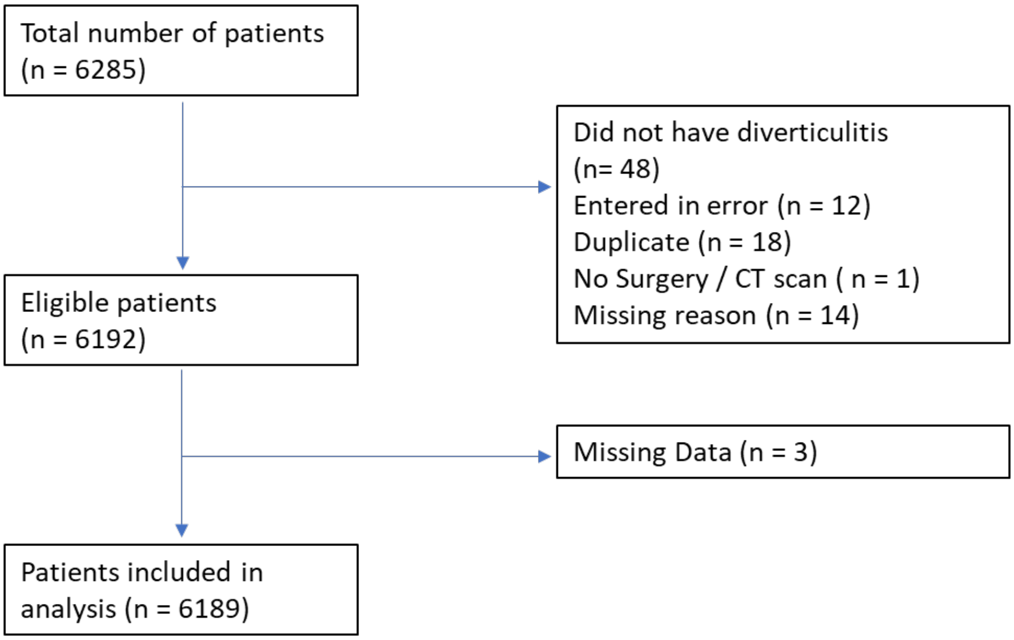

**Supplementary figure 2: Treatment failure in uncomplicated patients by treatment type, location and outcome**

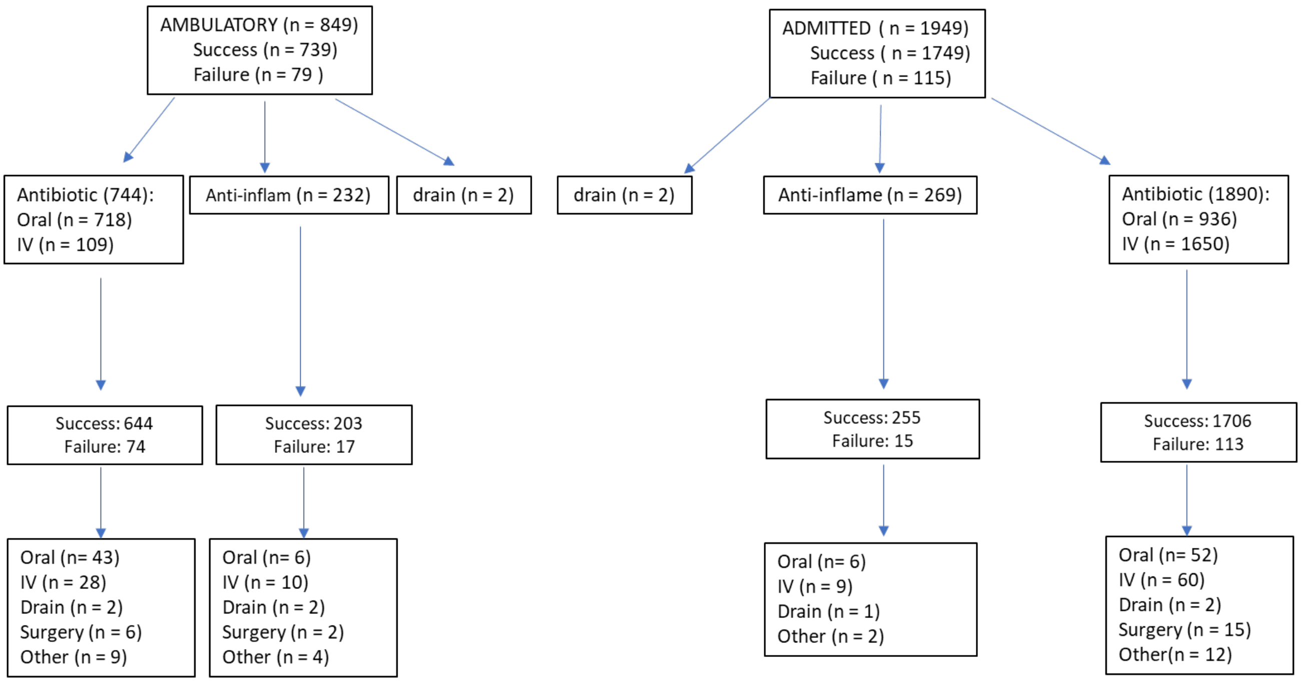

**Supplementary figure 3: Treatment failure in complicated patients by treatment type, location and outcome**

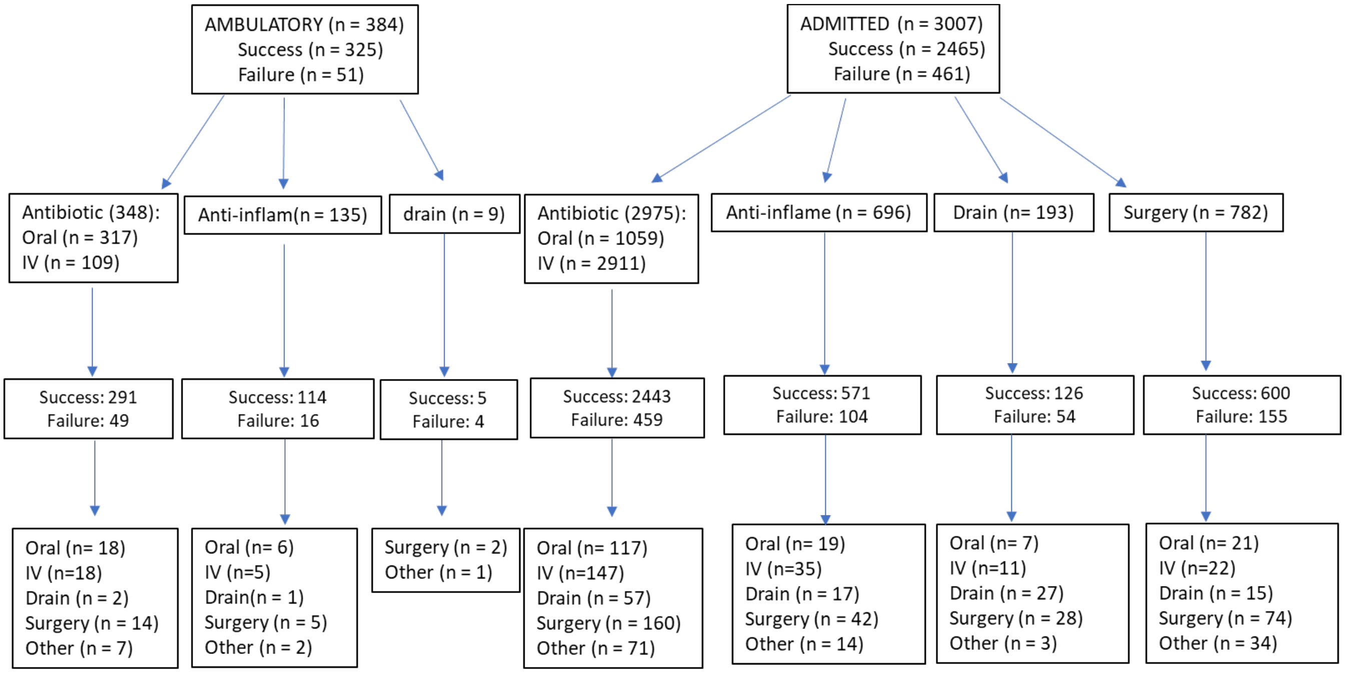

Supplement: Supplementary Material [file mmc1.pdf]
