# Supplementary material for: Global hospital-based disease management of acute diverticulitis: a prospective, international cohort study
Source: eClinicalMedicine. 2025 Sep 30;89:103548. doi: 10.1016/j.eclinm.2025.103548 (PMC12512976; doi:10.1016/j.eclinm.2025.103548)
Supplement: Study Protocol [file mmc2.pdf]

Check for updates

Received: 5 March 2021 | Revised: 9 April 2021 | Accepted: 20 April 2021
DOI: 10.1111/codi.15699

TRIAL PROTOCOL

Diverticulitis Management, a Snapshot Collaborative Audit Study (DAMASCUS): Protocol for an international, multicentre, prospective observational study

DAMASCUS Study Management Group

Correspondence
Dale Vimalachandran, University of Liverpool, Liverpool L69 3BX, UK
Email: dale.vimalachandran@nhs.net

Funding information
This study has been funded by Bowel Research UK. The study will be coordinated via Birmingham Surgical Trials Consortium and thus the burden of the cost will lie within the UK. Participating centres will not bear any costs for being part of this audit. Similarly, no financial reimbursement will be made to units or investigators for their involvement. The sponsor for DAMASCUS is the Countess of Chester NHS Foundation Trust. Neither the funder nor the sponsor have had any input in the design of the study or data analysis.

Abstract

Aim: Diverticular disease is an increasingly common problem in Western society with a variety of treatment options for those presenting with acute diverticulitis, dependent on clinical presentation. Additionally, there is significant international variability in the index management, and few published data on real-world clinical practice. The aim of DAMASCUS is to identify areas of practice variability and their potential association with differences in short- and medium-term clinical outcomes.

Methods and analysis: DAMASCUS is an international, collaborative, prospective observational study, recruiting patients from over 200 sites across six continents. The study opened in October 2020, with a rolling start. Identification of new sites ceased in February 2021 and data collection will cease in August 2021. All adult patients diagnosed with acute diverticulitis (radiologically or intra-operatively) at each participating centre will be included. The primary objective of DAMASCUS is to assess for national and international variability in the presentation and index management of acute diverticulitis (medical, interventional radiology and surgical). Secondary objectives include assessing 30-day and 6-month clinical outcome data (readmission, re-intervention, morbidity and mortality) and variations in surgical procedures for those undergoing surgery. All data will be recorded and managed using a secure REDCap electronic data capture tool and analysed using Stata (SE) version 16.1. The results will be reported in accordance with the STROBE statement.

Conclusion: By analysing variations in the management of acute diverticulitis and the subsequent outcomes, DAMASCUS will be an important step towards identifying optimal care for patients with diverticulitis.

KEYWORDS
acute diverticulitis, audit, diverticular disease, management, prospective

Protocol version: DAMASCUS Protocol Version 3.0 28/9/20

© 2021 Association of Coloproctology of Great Britain and Ireland.

INTRODUCTION

Diverticular disease is a common problem affecting up to 65% of people aged over 80 years [1] Complications may affect 10%–25% of these patients [2] and, although some such as bleeding and acute inflammation can usually be managed conservatively, others such as perforation are more serious. Perforation may present as peritonitis requiring emergency surgery but can also occur in a more indolent fashion as a sealed off abscess needing radiological drainage or a fistula into adjacent organs requiring interval resection.

The incidence of acute diverticulitis and hospital admissions for its complications are both steadily increasing, probably due to a population cohort that increasingly exhibits risk factors for complicated diverticular disease (age and obesity). The UK admission rate for acute diverticulitis has risen from 0.56 to 1.20/1000/year between 1996 and 2006 along with a 2.28-fold increase in admissions for perforated disease, equating to approximately 12,000 emergency bowel resections per year [3]. Perforated disease has an associated short-term (8.2%) [4] and long-term (14.5%) mortality rate [4,5] and these rates are particularly high in the UK. The exact cost to the National Health Service of this disease is unknown, but European and US studies have suggested that direct and indirect costs range from £63 million to over £1 billion per year, respectively [6,7].

Broad initial management strategies for acute diverticulitis can vary from conservative measures such as antibiotics and drainage procedures to more invasive surgical procedures such as laparoscopic lavage and bowel resection. Although there are several prospective studies advocating optimal treatment strategies, little is known about real-life clinical practice patterns. Retrospective analysis of administrative datasets has suggested significant international variability in the index management of acute diverticulitis, and this may contribute to the observed differences in mortality rates [4]. There are few prospective data, however, regarding this perceived international variation. Furthermore, very little is known about the optimal management of specific subgroups of patients with acute diverticulitis such as those with a diverticular abscess.

This study will audit the different types of management strategies employed in patients presenting with acute diverticulitis. The aim is to investigate international variation in presentation and management and explore associations between index management choices and short- and medium-term clinical outcomes. Evidence of potential predictive factors may provide strong support for future prospective, pragmatic clinical trials looking to inform the definition of gold standard management in acute diverticulitis.

METHODS

Study design

DAMASCUS is an international, multicentre, prospective audit aiming to collect short-term 30-day and 6-month clinical outcome data of patients presenting to hospital with acute diverticulitis. In addition, it will capture national and international variability in the presentation and management of acute diverticulitis. DAMASCUS is open to participation by all centres that admit patients with acute diverticulitis. The study opened in October 2020, with a rolling start to accommodate the COVID-19 pandemic. The identification of new sites ceased on 28 February 2021, with any sites starting recruitment after this date having a reduced recruitment period ending in August 2021. Routine patient and clinical data will be recorded and managed using Research Electronic Data Capture (REDCap) hosted at Birmingham Centre for Observational and Prospective Studies (BiCOPS) [8,9]. REDCap is a secure, web-based software platform designed to support data capture for research studies on bespoke case report forms (CRFs). In the light of the COVID-19 pandemic, this audit will also seek to collect baseline demographic and initial management data relating to both patient and unit-level COVID-19 status. The results of DAMASCUS will be reported in accordance with the STROBE (Strengthening the Reporting of Observational studies in Epidemiology) statement [10].

Study population

The study opened to all sites globally in October 2020. It is anticipated that the DAMASCUS collaborative will recruit approximately 4000 patients, from at least 200 sites, from the continents of Europe, Australasia, Africa, Asia and the Americas.

Inclusion criteria

All adult patients (defined as 18 years and above) presenting with acute diverticulitis (newly incident within the audit period) to a participating site will be included in the study, regardless of severity (acute uncomplicated through to faecal peritonitis) and subsequent treatment (e.g., admission vs. discharge, surgery vs. no surgery). Diagnosis of acute diverticulitis needs to be confirmed either via multiplanar CT scans or during emergency surgery. Patients admitted previously with acute diverticulitis who re-present with a new episode of acute diverticulitis will also be included.

Exclusion criteria

Patients with diverticulosis only (the presence of colonic diverticulum without evidence of acute inflammation or complication) on CT scan will not be included. Additionally, patients with bowel perforation due to other bowel conditions (e.g., perforated tumours, inflammatory bowel disease or bowel ischaemia) will also be excluded.

Patient pathway and identification

Following confirmation of the diagnosis and eligibility, patient data will be collected for the audit as patients progress through local

14631318, 2021, 8, Downloaded from https://onlinelibrary.wiley.com/doi/10.1111/codi.15699 by <Shibboleth>-member@liv.ac.uk, Wiley Online Library on [07/09/2025]. See the Terms and Conditions (https://onlinelibrary.wiley.com/terms-and-conditions) on Wiley Online Library for rules of use; OA articles are governed by the applicable Creative Commons License

routine care pathways. Patients may present with a range of severity from relatively mild diverticulitis through to localized abscess formation or peritonitis. Consequently, treatment may range from intravenous antibiotics, percutaneous drainage or surgery and these treatments may occur in single, combined or sequential regimens. Each treatment regimen will be recorded and, if patients are readmitted within 6 months, data regarding any treatment or interventions will also be recorded. Patients who are admitted acutely with peritonitis and undergo emergency surgery without any form of imaging and are subsequently found to have acute diverticulitis will also be included following surgery and/or pathological diagnosis (Figure 1).

Study variables

The data collected by the study will include patient characteristics, diagnosis and severity of diverticulitis, the treatment received and the associated outcomes. Patients included in the study will be followed-up for 6 months from the date of the first diagnosis. Follow-up data will include hospital readmissions and complications related to acute diverticulitis.

These data will be collected prospectively using electronic secure REDCap accounts. The summary of the study CRFs is given in Appendix S1.

Baseline data

Baseline data will be recorded at the index admission following confirmation of eligibility (Figure 2). Electronic CRFs (incorporating baseline, 30-day outcome data and 6-monthly outcomes) will be used by the clinical care team to capture data on (a) the patient (fitness, frailty and main risk factors for disease recurrence [previous admissions, smoking, non-steroidal anti-inflammatory drug use [11] and obesity [12]; (b) the disease (including sepsis markers and CT findings); (c) the index management and any operative findings and strategy; (d) 30-day outcome (routine perioperative measures); and (e) 6-month readmission/re-intervention rates. This CRF (Appendix S1) has been minimalized to facilitate rapid completion based on the principles embodied by other large-scale studies in the emergency setting (e.g., NELA [13]. Only routine data will be collected; no additional information will be sought as this is an audit of practice only.

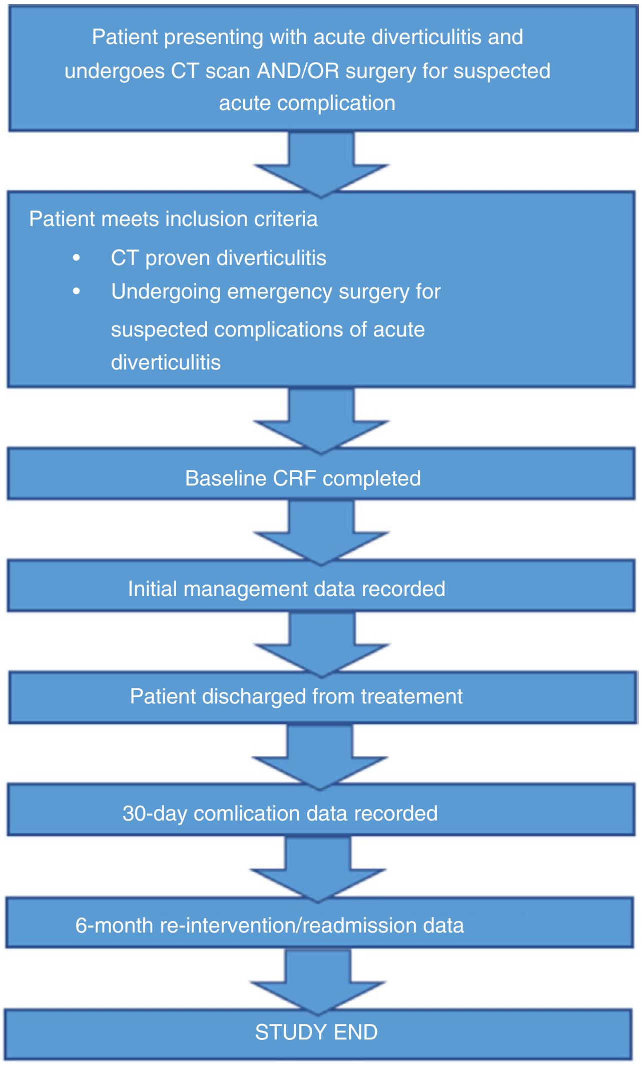

FIGURE 1 Patient identification and pathway

Patient variables		
		Age
		Weight
		Height
		Sex
		Ethnicity
		Smoking status
		Co-morbidities – described using Charlson Comorbidity Index
		Covid-19 status
		Country
Medical variables		
	Clinical parameters on admission	
		Temperature
		Blood pressure
		Respiratory rate
		Altered Glasgow Coma Score (GCS)
	Baseline laboratory markers	
		White Cell Count (WCC)
		C-Reactive Protein (CRP)
Disease variables		
	Previous admission with diverticulitis	
		How long ago was the last presentation/admission
		Number of presentations/admissions within the last 12 months
		Treatment received over the last 12 months
	CT and/or operative findings	
		Part of the colon affected
		- Inflammatory stranding
		- Phlegmon mass
		- Abscess including size, location and number
		- Presence of free fluid, volume and location
		- Presence extra-luminal gas, volume and location
		- Presence of a visible perforation and size

FIGURE 2 Patient, medical and disease covariates

14631318, 2021, 8, Downloaded from https://onlinelibrary.wiley.com/doi/10.1111/codi.15699 by <Shibboleth>-member@liv.ac.uk, Wiley Online Library on [07/09/2025]. See the Terms and Conditions (https://onlinelibrary.wiley.com/terms-and-conditions) on Wiley Online Library for rules of use; OA articles are governed by the applicable Creative Commons License

Objectives

The primary objective of DAMASCUS is to prospectively audit the national and international variability in the presentation and index management of acute diverticulitis. Secondary objectives include assessing the 30-day and 6-month outcomes (below) following index management and to detail national and international variation in surgical procedure type for those patients undergoing surgery.

Study outcomes

The study outcomes will include the following:

1. main disease state and trait characteristics at index presentation
2. initial management at index presentation
3. length of stay
4. post-intervention complication rates [14]
5. the 30-day recurrent admission rate (as a surrogate marker of all health utilization)
6. the 30-day recurrent admission rate for diverticulitis and complications (as a surrogate of disease-specific health utilization)
7. the 30-day recurrent intervention rate
8. the 6-month readmission and/or re-intervention rate
9. patient or disease-specific covariates associated with treatment strategy or short-term clinical outcomes
10. the impact of the COVID-19 pandemic on the management of acute diverticulitis
11. clinician equipoise for the recruitment of patients to future randomized controlled trials.

Data handling

Data will be entered directly onto the DAMASCUS REDCap database by study collaborators at the participating hospital sites. Collaborators will be provided with a paper copy of the eCRF to facilitate data collection. If this is used, they should then transfer data from the paper CRF to the online DAMASCUS database located at https://www.bicops.redcap.bham.ac.uk. DAMASCUS data management staff will check all incoming CRF data for completeness, data consistency and compliance with the protocol. If discrepancies or missing data are identified, the DAMASCUS data management staff will raise queries with the research team at the participating hospital.

Data management

The security of the study database system is governed by the policies of the University of Birmingham. The DAMASCUS study database will be hosted on the university's REDCap system managed and maintained by BiCOPS.

Data management and security within BiCOPS will abide by the requirements of the General Data Protection Regulations and any subsequent amendments. The study will be conducted at collaborating sites following country-specific data protection requirements. Data will be acquired and stored on the REDCap platform. Access to data will be restricted; each individual collaborator entering data for DAMASCUS will have their own username and password. Each patient will be allocated a unique study number at entry. All communication will use this as the identifier. All data will be analysed and reported in summary format. No individual will be identifiable.

Data analysis plan

Data analysis will primarily explore variations in clinical practice at a country or continent level. Secondly, we aim to evaluate treatment failure rate. This will be defined as any escalation of treatment from the index admission until the 6-month follow-up is complete. We will explore baseline covariants that may be predictive of failure, including patient demographics and clinical parameters. For the purposes of subgroup analyses, radiological or operative findings will be subsequently categorized according to the modified Hinchey classification. Furthermore, we will evaluate 6-month readmission rates from a health economics point of view and 30-day mortality rates.

Statistical analysis

Statistical analysis will be undertaken by our international management group which includes statisticians at the Institute of Applied Health Research, University of Birmingham. It will be performed on Stata (SE) version 16.1 (Stata Corp. LLC, College Station, Texas, USA). No surgeon- or hospital-specific comparisons will be performed, and analyses will only be reported at a country level for those in which at least 15 patients in total, from a minimum of three hospitals, have been included in the final dataset.

Descriptive analysis

Descriptive statistics will be used to summarize demographic, lifestyle, medical characteristics and outcomes by country or continent. Mean and standard deviation (normally distributed data) or median and interquartile range (non-normally distributed data) will be used to summarize continuous data. Frequencies and percentages will be used to summarize categorical data. The chi-squared test will be used to compare categorical data. Continuous, normally distributed data will be compared using the *t* test or ANOVA while non-normally distributed data will be compared using the Mann–Whitney *U* test or Kruskal–Wallis test as appropriate.

14631318, 2021, 8, Downloaded from https://onlinelibrary.wiley.com/doi/10.1111/codi.15699 by <Shibboleth>-member@liv.ac.uk, Wiley Online Library on [07/09/2025]. See the Terms and Conditions (https://onlinelibrary.wiley.com/terms-and-conditions) on Wiley Online Library for rules of use; OA articles are governed by the applicable Creative Commons License

Univariable and multivariable analyses

Logistic regression models will be developed to examine the relationship between treatment failure (yes/no) and independent predictors. Treatment failure will be defined as escalation of therapy if it was initially non-operative. The independent predictor variables will include demographic, lifestyle, medical and disease characteristics. For all analyses, a two-sided p value of 0.05 will be considered as statistically significant.

Missing data

We anticipate that a few key variables may have a significant proportion of missing data. Multiple imputation is the preferred method to deal with missing data in order to minimize bias [15]. If there is a considerable amount of missing data, multiple imputation techniques will be used as appropriate.

Implausible values and quality checks

Implausible physiological or laboratory measurements, outside of pre-determined cut-off ranges, will be identified and assigned a missing value status.

Ethics

In the UK, this study is categorized as an audit, not research (see Appendix S2 for the Health Research Authority decision tool outcome). Therefore, sites may participate once local clinical audit approval is in place. Non-UK centres should seek advice from their local regulatory bodies (ethical committees) and apply for the required approvals according to local, state or national policy before the study starts. For centres in the United States, surgeons must obtain Institutional Review Board approval before enrolling patients.

Only routinely collected data will be collected in the DAMASCUS study. Patients will not undergo any additional investigations or clinical follow-up for the study. No sensitive or identifiable data will be collected on the REDCap database. We anticipate that most ethics review boards will waive the requirement for patient consent, as only anonymized audit data will be collected. However, there may be variation in international regulations, and it will be the responsibility of the local principal investigators to seek advice from the local research ethics committee in each participating country to determine whether informed consent should be sought.

Funding

This study has been funded by the UK-registered charity Bowel Research UK (no. 1186061). in the UK. The study will be coordinated via BiCOPS and thus the burden of the cost will lie within the UK. Participating centres will not bear any costs for being part of this audit. Similarly, no financial reimbursement will be made to units or investigators for their involvement.

Result dissemination

The results of this study will be submitted for publication in a peer-reviewed scientific journal. Given the international nature of the study it is anticipated that this will be reflected in the journal selected. Results of the study will also be presented at both national and international meetings, with the assistance of investigators in the contributing nations. The findings of this study may be used to inform the design of further studies into diverticular disease.

Authorship policy

The chief investigators will coordinate the dissemination of data from the study. All publications using data from the study to undertake original analyses will be submitted to the Study Management Group (SMG) for review before release. The success of the study depends on a large number of clinicians. For this reason, credit for the results will not be given to the committees or central organizers but to all who have collaborated and participated in the study. The acknowledgement will include all local coordinators and collaborators, members of the study committees, country leads, the SMG and administrative staff. Authorship at the head of the primary results paper will be cited as a collaborative group to avoid giving undue prominence to any individual. All contributors to the trial will be listed at the end of the report, with their contribution to the study identified. Those responsible for other publications reporting specific aspects of the study may wish to utilize a different authorship model, such as '[name], [name] and [name] on behalf of the collaborative group'. Decisions about the authorship of additional papers will be discussed and agreed by the study investigators and the SMG.

Discussion

Diverticular disease and its complications account for an increasing number of hospital admissions globally. This is in part due to the obesity pandemic, an ageing population, low dietary fibre, lack of exercise and smoking. In the UK alone, the number of patients with a primary diagnosis of diverticular disease doubled in the period 1996–2006 to a rate of 1.2 per 1000 per year [3]. Additionally, age at first presentation is becoming significantly younger [3,4,15]. With this increase in incidence we see the associated financial burden resulting from recurrent admissions and in 16.3% of cases the need for surgery [3] all of which carry a risk of concomitant mortality. Colostomy rates for those undergoing surgery have remained static at 56% which is a cause of significant morbidity for many patients [16].

14631318, 2021, 8, Downloaded from https://onlinelibrary.wiley.com/doi/10.1111/codi.15699 by <Shibboleth>-member@liv.ac.uk, Wiley Online Library on [07/09/2025]. See the Terms and Conditions (https://onlinelibrary.wiley.com/terms-and-conditions) on Wiley Online Library for rules of use; OA articles are governed by the applicable Creative Commons License

Although diverticular disease is common, it is still poorly understood. Many important questions remain regarding the definition of optimal care. This audit aims to obtain data for over 4000 patients, which will be the largest prospectively obtained diverticular database so far. This large number of patients will be invaluable and will improve the generalizability and applicability of any trends that it may identify or recommendations it may make. It aims primarily to identify any international variability of the management of diverticulitis at the index admission. Any prior attempts at this have been retrospective in nature. Hong et al. [4] identified significantly higher emergency operative rates in the United States, particularly for the uncomplicated disease, compared to Australia and England. However, England had higher operative rates for complicated disease and a higher inpatient mortality rate. The main limitation of that study was that it relied on coding which in turn is influenced by financial drivers. Additionally, it was unable to provide details of thresholds for admission, Hinchey grading, type of operative intervention or palliative treatments. Despite this variation over recent years, there has been a reduction in emergency operative intervention with a rise in a trial of conservative management in clinically stable patients, without an increase in mortality [16,17].

Secondly, DAMASCUS aims to evaluate treatment failure rates from index admission until follow-up and to identify any covariants identified with failure. Evidence of potential predictive factors may provide strong support for future prospective, pragmatic clinical trials looking to inform the definition of gold standard management in acute diverticulitis. One important unanswered question which remains an issue of equipoise amongst colorectal surgeons is if or when to operate on patients presenting with a diverticular abscess. To date, the largest prospective study that included patients presenting with Hinchey Ib/II was published in 2005 by Ambrosetti et al. [18] Of those presenting with a pelvic abscess, 71% of patients required surgery either acutely or during the follow-up period. Other large studies focusing on this cohort of patients have been retrospective with varying definitions of failure of non-operative treatment. Additionally, guidelines regarding the management of diverticular abscesses vary between countries. In 2020 the American Society of Colon and Rectal Surgeons published practice parameters for diverticulitis and recommended that an elective resection should be considered following non-operative management of a diverticular abscess (level 1B) [19]. In 2019 the National Institute of Clinical Excellence also advised that patients should be considered for elective resection once recovered from the acute complicated episode if symptoms persist, although this included all complications and was not limited to abscesses [20]. Conversely, the European Association for Endoscopic Surgery and the Society of American Gastrointestinal and Endoscopic Surgeons reached a consensus that, following a single episode of Hinchey Ib/II diverticulitis which was successfully managed conservatively, a patient should not routinely be offered surgery [21]. This conclusion was also reached in guidelines published in 2020 by the European Society of Coloproctology [22].

DAMASCUS is primarily a research project investigating variation in management of acute disease, but in developing it we have developed relationships with collaborators and used DAMASCUS as a platform to aid recruitment and implementation of secondary studies investigating diverticular disease. To date, the authors have been involved in qualitative research with patient and public involvement days and the EXPLODES study investigating the epigenetics of diverticulitis.

The limitations of this study lie with the inherent biases that cohort studies carry. DAMASCUS is reliant on the collaborative efforts of healthcare professionals around the world. Therefore, to reduce selection bias, all patients who present to the enrolled site must be inputted. Similarly, all follow-up data must be collected to reduce information bias, which may impact on the accuracy of the data. The follow-up period of 6 months has been deemed appropriate to capture most readmissions. However, patients may require readmission after 6 months which may introduce information bias as these data will not be entered. However, unlike many cohort studies, the large scale of this study will give the results external validity and capture outcomes of less common presentations such as diverticular abscesses. In addition to this, the inclusion criteria of CT-confirmed diverticulitis are well defined and are not open to interpretation, thereby reducing selection bias. Steps to further reduce the risk of bias introduced by data collection include the use of an online database for data collection (REDCap). This standardizes data collected and once inputted they can be analysed by the primary investigative team.

DAMASCUS and the number of patients it hopes to recruit over the 6-month period are only possible through collaboration. Collaborative studies are becoming increasingly popular and shared authorships are now commonplace. These studies often deliver high-volume data and can observe practices and outcomes from a wide range of centres, or in the case of DAMASCUS from around the world. This of course is only possible by having healthcare workers around the globe who are passionate about research and improving outcomes for their patients, to whom we are grateful.

ACKNOWLEDGEMENTS

We would like to acknowledge the support of the Dukes Club, the European Society of Coloproctology cohort committee, the Colorectal Surgical Society of Australia and New Zealand and the American Society of Colon and Rectal Surgeons. Steering committee and writing group: Mohamed Rabie, Hayley Fowler, Nagendra N Dudi-Venkata, John Ayorinde, Mohamedraed Elshami, Debby S Keller, Patricia Sylla, Gaetano Gallo, Francesco Pata, Kelvin Okoth, Tarik Sammour, Roberto Bergamaschi, Guy Orangio, Thomas Pinkney, Charles H Knowles and Dale Vimalachandran. Study delivery and data management group: Laura Magill, Kelvin Okoth, Rita Perry, Michala Pettitt and Michael Walters. Dissemination committee and national leads: Algeria, Salah Eddine Kacimi; Australia, Nagendra Dudi-Venkata; Egypt, Mohamed Fahmy Doheim; Germany, Ulrich Ronellenfitsch; Greece, Theodoros Sidiropoulos; Ireland, Gerard Sexton; Italy, Gaetano Gallo, Francesco Pata; Jordan, Amro Abuleil; Lebanon, Lina Karout; Libya, Muhammed Elhadi; Middle East/North Africa, Mohamedraed Elshami; Palestine, Mohammed Alser; Spain, Alajandro Sánchez Arteaga; Sudan, Mahmoud Saleh; Switzerland, Eleftherios Gialamas; Syria, Alaa Hamdan; Turkey, Mert Tanal; UK, Mohamed Rabie; Yemen, Hamza Al-Nagga

14631318, 2021, 8, Downloaded from https://onlinelibrary.wiley.com/doi/10.1111/codi.15699 by <Shibboleth>-member@liv.ac.uk, Wiley Online Library on [07/09/2025]. See the Terms and Conditions (https://onlinelibrary.wiley.com/terms-and-conditions) on Wiley Online Library for rules of use; OA articles are governed by the applicable Creative Commons License

CONFLICT OF INTERESTS

The authors have no competing interests to declare.

ETHICAL APPROVAL

Ethical approval for this study has been obtained in those countries where it is required. In the United Kingdom the study has been registered as an audit no identifiable data will be collected.

AUTHOR CONTRIBUTIONS

All authors listed in the writing group and study delivery team have contributed equally to the design of the study and have been involved in drafting the manuscript to ensure the intellectual content is satisfactory. All authors were involved in the final approval of the published version.

DATA AVAILABILITY STATEMENT

Data sharing is not applicable to this article as no datasets were generated or analysed during the current study.

REFERENCES

1. Munie ST, Nalamati SPM. Epidemiology and pathophysiology of diverticular disease. Clin Colon Rectal Surg. 2018;31(4):209–13.
2. Ferzoco LB, Raptopoulos V, Silen W. Acute diverticulitis. N Engl J Med. 1998;338:1521–6.
3. Jeyarajah S, Faiz O, Bottle A, Aylin P, Bjarnason I, Tekkis PP, et al. Diverticular disease hospital admissions are increasing, with poor outcomes in the elderly and emergency admissions. Aliment Pharmacol Ther. 2009;30(11–12):1171–82.
4. Hong MKY, Skandarajah AR, Higgins RD, Faiz OD, Hayes IP. International variation in emergency operation rates for acute diverticulitis: insights into healthcare value. World J Surg. 2017;41(8):2121–7.
5. Gregersen R, Andresen K, Burcharth J, Pommergaard HC, Rosenberg J. Long-term mortality and recurrence in patients treated for colonic diverticulitis with abscess formation: a nationwide register-based cohort study. Int J Colorectal Dis. 2018;33(4):431–40.
6. Mennini FS, Sciattella P, Marcellusic A, Toraldo B, Koch M. Economic burden of diverticular disease: an observational analysis based on real world data from an Italian region. Dig Liver Dis. 2017;49:1003–8.
7. Reddy VB, Longo WE. The burden of diverticular disease on patients and healthcare systems. Gastroenterol Hepatol. 2013;9:21–7.
8. Harris PA, Taylor R, Thielke R, Payne J, Gonzalez N, Conde JG. Research electronic data capture (REDCap)—a metadata-driven methodology and workflow process for providing translational research informatics support. J Biomed Inform. 2009;42(2):377–81.
9. Harris PA, Taylor R, Minor BL, Elliott V, Fernandez M, O'Neal L, et al. The REDCap consortium: building an international community of software partners. J Biomed Inform. 2019;95:103208. https://doi.org/10.1016/j.jbi.2019.103208
10. vom Elm E, Altman DG, Egger M, Pocock SJ, Gotzsche PC, Vedenbroucke JP, et al. The Strengthening the Reporting of Observational Studies in Epidemiology (STROBE) statement: guidelines for reporting observational studies. J Clin Epidemiol. 2008;61(4):344–9.PMID: 18313558
11. You K, Bendl R, Taut C, Sullivan R, Gachabayov M, Bergamaschi R, et al. Randomized clinical trial of elective resection versus observation in diverticulitis with extraluminal air or abscess initially managed conservatively. Br J Surg. 2018;105(8):971–9.
12. Strate LL, Liu YL, Aldoori WH, Syngal S, Giovannucci EL. Obesity increases the risks of diverticulitis and diverticular bleeding. Gastroenterology. 2009;136(1):115–122.e1. https://doi.org/10.1053/j.gastro.2008.09.025
13. Peacock O, Bassett MG, Kuryba A, Walker K, Davies E, Anderson I, et al. Thirty-day mortality in patients undergoing laparotomy for small bowel obstruction. Br J Surg. 2018;105(8):1006–13. https://doi.org/10.1002/bjs.10812
14. Dindo D, Demartines N, Clavien P-A. Classification of surgical complications: a new proposal with evaluation in a cohort of 6336 patients and results of a survey. Ann Surg. 2004;240(2):205–13. Accessed February 9, 2018.
15. Jakobsen JC, Gluud C, Wetterslev J, Winkel P. When and how should multiple imputation be used for handling missing data in randomised clinical trials—a practical guide with flowcharts. BMC Med Res Methodol. 2017;17(1):162.
16. Etzioni D, Mack T, Beart R, Kaiser A. Diverticulitis in the United States: 1998–2005: changing patterns of disease and treatment. Ann Surg. 2009; 249(2):210–7.
17. Li D, Baxter N, McLeod R, Moineddin R, Wilton A, Nathens A. Evolving practice patterns in the management of acute colonic diverticulitis: a population-based analysis. Dis Colon Rectum. 2014;57(12):1397–1405.
18. Ambrosetti P, Chautems R, Soravia C, Peiris-Waser N, Terrier F. Long-term outcome of mesocolic and pelvic diverticular abscesses of the left colon: a prospective study of 73 cases. Dis Colon Rectum. 2005;48(4):787–91.
19. Hall J, Hardiman K, Lee S, Lightner A, Stocchi L, Paquette IM, et al. Prepared on behalf of the Clinical Practice Guidelines Committee of the American Society of Colon and Rectal Surgeons. The American Society of Colon and Rectal Surgeons Clinical Practice Guidelines for the Treatment of Left-Sided Colonic Diverticulitis. Dis Colon Rectum. 2020;63:728–47.
20. National Institute for Health and Care Excellence. NICE Guideline. Diverticular disease: diagnosis and management; 2019.
21. Francis N, Sylla P, Abou-Khalil M, Arolfo S, Berler D, Curtis N, et al. EAES and SAGES 2018 consensus conference on acute diverticulitis management: evidence-based recommendations for clinical practice. Surg Endosc. 2018;2019(33):2726–41.
22. Schultz JK, Azhar N, Binda GA, Barbara G, Biondo S, Boermeester MA, et al. European Society of Coloproctology: guidelines for the management of diverticular disease of the colon. Colorectal Dis. 2020; 22: 5–28. https://doi.org/10.1111/codi.15140

SUPPORTING INFORMATION

Additional supporting information may be found online in the Supporting Information section.

How to cite this article: DAMASCUS Study Management Group. Diverticulitis Management, a Snapshot Collaborative Audit Study (DAMASCUS): Protocol for an international, multicentre, prospective observational study. *Colorectal Dis.* 2021;23:2182–2188. https://doi.org/10.1111/codi.15699

14631318, 2021, 8, Downloaded from https://onlinelibrary.wiley.com/doi/10.1111/codi.15699 by <Shibboleth>-member@liv.ac.uk, Wiley Online Library on [07/09/2025]. See the Terms and Conditions (https://onlinelibrary.wiley.com/terms-and-conditions) on Wiley Online Library for rules of use; OA articles are governed by the applicable Creative Commons License
