# Supplementary material for: Global hospital-based disease management of acute diverticulitis: a prospective, international cohort study
Source: eClinicalMedicine. 2025 Sep 30;89:103548. doi: 10.1016/j.eclinm.2025.103548 (PMC12512976; doi:10.1016/j.eclinm.2025.103548)
Supplement: Pubmed Citable Author List [file mmc3.docx]

Pubmed Citable Authors

| A Lawgaly | Sami |
| --- | --- |
| A.      Monib | Fatma |
| Aakif | Muhammad |
| Ababneh | Hazim |
| Ababneh | Malak |
| Abakar | Mourtada |
| Abd elhady | Hamdy |
| Abdel-Aziz | Mohamed |
| Abdelbaset | Mai |
| Abdel-Maboud | Mohamed |
| Abdel-Maboud | Mohamed |
| Abdelmalak | Mina Ragaa Fekry |
| Abd-elsalam | Sherief |
| Abdelzaher | Abdurrahman |
| Abdul Aal | Yasser |
| Abdulwahed | Eman |
| Abete | Roberta |
| Abo Ali | Amira |
| Abotaleb | Khadega |
| Abu Elnaga | Nagm Eldin |
| Abu Hmaid | Amer |
| Abu Ismail | Dima Y |
| Abu mahfouz | Dema |
| Abu nawas | Duaa |
| Abu Selmiyh | Hala |
| Abu-Ismail | Luai |
| ABULEIL | AMRO |
| Abwini | Mahmoud S |
| Acharya | Shivanie |
| Acosta | Lina |
| Adam | Alexis |
| Adams | Katie |
| Adams | Clare |
| Adegbola | Samuel |
| Adel Jabr Bin Jabr | Ala'a |
| Adel Mohammed | Yasmine |
| Adeyeye | Ademola |
| Adeyeye | Rebecca |
| Adiamah | Alfred |
| Adwi | Mohamed |
| Afify | Emma |
| Afzal | Mohamed |
| Ahmad | Shahrukh |
| Ahmad | Bisan |
| Ahmed | Rawan |
| Ahmed | Rizwan |
| Ahmed | Nauman |
| Ahmed | Jamil |
| Ahonkhai | Irele-Ifijeh |
| Aigner | Felix |
| Ainsworth | Paul |
| Akgun | Erhan |
| Akin | Emrah |
| Akingboye | Akinfemi |
| Akinmade | Akinola |
| Akmercan | Ahmet |
| Aktimur | Yunus |
| Aktokmakyan | Talar |
| Al daif alla | Abdallah |
| Al Hashemi | Mohamad |
| Aladini | Mohammed |
| Alajandro | Artega Sanchez |
| Alaklook | Safa |
| Alaklouk | Marwa |
| Alam | Mushfique |
| Al-Amiedy | Zaid |
| Alan Koylu | Zehra |
| Alarood | Salameh |
| Alawashreh | Mohammad |
| Alazzaq | Youssef |
| Albakry | Rudaina |
| Albarki | Akram |
| Albarracín Marín-Blázquez | Antonio |
| Albendary | Mohamed |
| Albirnawi | Hatim |
| Al-dhaheri | Manal Jamal |
| Aldressi | Wafa |
| Aldressi | Sarah |
| Alemad | Shada |
| Al-Eryani | Fatima |
| Alfatih Hamza | Mohamed |
| Alghazawi | Laith |
| Algul | Begum |
| Alhabil | Belal |
| Alhasy | Naser |
| Al-Hayek | Tahani |
| Ali | Roshneen |
| Ali Alshareea | Entisar Ahmed |
| Ali karar | Ali Adil |
| Aljaiuossi | Anas |
| Alkaseek | Akram |
| Alkhaldi | Muzan |
| Al-Kholey | Ahmed Emad |
| Alkikle | Reem |
| Al-Kubati | Waheeb |
| Allam | Mohamed |
| Al-Lamee | Noor |
| Allmer | Caterina |
| Allocco | Roberto |
| Allon | Oliver |
| Alma'aitah | Fares |
| Almaghrebi | Asem |
| Almallah | Ahmed |
| ALMI'ANI | SARI |
| Al-Nagga | Hamza |
| Al-nahwi | Ghofran |
| AlNajem | Hafssah |
| Alnuweiri | Manal |
| Alqadi | Zaid |
| Alqady | Eithar |
| Al-Qasrawi | Shahd |
| Alqudah | Majdi |
| Al-Sadawi | Mohammed |
| Alsadek | Mohamed |
| Alser | Mohammed |
| Alshaikh | Bushra |
| Al-Shehari | Mohammed Mohammed |
| Alsulaim | Hatim |
| Altinel | Yuksel |
| Altintoprak | Fatih |
| Altiti | Raed |
| Altomare | Donato F |
| Altubi | Ikhtiyar |
| Alvarez-Bautista | Francisco |
| Al-Wahedi | Abdulwahid |
| Al-wajeeh | Ghadeer |
| Al-Wandi | Amna |
| Alwhouhayb | Maitham |
| Alzahran | Ayham |
| Amer | Mostafa |
| Amir | Farhat |
| Ammar | Ahmed |
| Amprayil | Mathew |
| Amro | Sarah |
| Anan | Asmaa |
| Anestiadou | Elissavet |
| Annese | Sergio |
| Annicchiarico | Alfredo |
| Apestegui | Carlos Alejandro |
| Aremu | Muyiwa |
| Arkadopoulos | Nikolaos |
| Arslan | Kemal |
| Ashcroft | James |
| Assaf | Nazrin |
| Assaker | Jordan |
| Avellaneda | Nicolas |
| Awad | Ahmed K. |
| Awadallah | lsam |
| Awbakh | Mirna |
| Aybar | Engin |
| Ayeni | Adewale |
| Ayorinde | Tobi |
| Ayoub | Kusay |
| Babikir | Mohammed A. |
| Badenoch | Thomas |
| Bader | Franz |
| Badiani | Sarit |
| Badr | Helmy |
| Badran | Yousef Sameh |
| Badrinath | Krishnamurthy |
| Baeza Murcia | Melody |
| Baggaley | Alice |
| Baig | Mirza |
| Bailey | James |
| Baili | Efstratia |
| Bakewell | Zoe |
| Bakheit | Imad M. |
| Balasubramanya | Supriya |
| Balık | Emre |
| Baloyiannis | Ioannis |
| Banks | Jessica |
| Baran | Elif |
| Barbaro | Antonio |
| Barker | Jonathan |
| Barlow | Emma |
| Barnes | Thomas |
| Bartsch | Claudia |
| Bashir | Manahil |
| Bassiony | Mahmoud |
| Batra | Paras |
| Bauza-Collado | Mireia |
| Bayhan | Zulfu |
| Bayraktar | Yahya Alperen |
| Bayram | Onur |
| Beddy | David |
| Belhasan | Anas |
| Bell | Zara |
| Beltrán de Heredia | Juan |
| Belvedere | Angela |
| Benavides Buleje | Jorge Alejandro |
| Benitez-Riesco | Ana |
| Bennett | Henry |
| Ben-Sassi | Abozed |
| Bergmann | Nicole |
| Bermejo Marcos | Elena |
| Berney | Christophe |
| Betoret | Lidia |
| Bevier-Rawls | Elyse |
| Bhasin | Swati |
| Bhasin | Deepika |
| Bhatta | Gakul |
| Bhattacharya | Pratik |
| Biala | MarwaIsa |
| Bianco | Francesco |
| Bierton | Christopher |
| Bilton | Henry |
| Binder | Alf-Dorian |
| Birrer | Dominique |
| Blake | Iain |
| Blázquez-Martín | Alma |
| Bleakley | Anna |
| Bogdan | Monica |
| Bonasso | Carlotta |
| Bonati | Elena |
| Bond | Richard |
| Bond-smith | Giles |
| Bonner | Clare |
| Bonomi | Alessandro Michele |
| Borakati | Aditya |
| Borghi | Felice |
| Borowski | David W |
| Boura | Maria |
| Boushnaq | Mohammed |
| Boutros | Marylise |
| Boyes | Joshua |
| Bozbiyik | Osman |
| Bozkurt | Mehmet Abdussamet |
| Bozkurt | Emre |
| Bracale | Umberto |
| Brachini | Gioia |
| Bravo-Avila | Hector |
| Brett | Aishling |
| Broadbent | Jack |
| Broadhurst | Damian |
| Brown | Ben |
| Bruzzese | Giuseppe |
| Bughio | Mumtaz |
| Buijs | Louis F |
| Buwaitel | Mohammad |
| Bylapudi | Seshu |
| Byrne | Jim |
| Cabezudo | Guillermo |
| Cabrera | Paulo |
| Calabrò | Marcello |
| Calikoglu | Fikret |
| Calikoglu | Tugba |
| Caliskan | Cemil |
| Campbell | Abigail |
| Canas-Martinez | Angela |
| Candan | Mert |
| Cannavera | Alessandro |
| Cannavo | Maurizio |
| Cantero | Ramon |
| Capoglu | Recayi |
| Capolupo | Gabriella Teresa |
| Carannante | Filippo |
| Cardia | Roberto |
| Carlini | Massimo |
| Carrasco Prats | María Milagros |
| Carrie | Augusto |
| Carvello | Michele |
| Casali | Lorenzo |
| Casas | Felipe |
| Caserini | Ottavia |
| Castelhano | Rute |
| Castro-Suárez | Marta |
| Catena | Fausto |
| Cayetano Paniagua | Ladislao |
| Cerdán Santacruz | Carlos |
| Cervellera | Maurizio |
| Chadwick | Michael |
| Chang | Jessica |
| Chan-Thu | Aye |
| Chapman | Polly |
| Chappaley | Dimitri |
| Charalabopoulos | Alexandros |
| Chase | Thomas |
| Chatzmichail | Theodora |
| Chautems | Roland |
| Chavarria | Nuria |
| Cheong | Julia |
| Cholewa | Hanna |
| Christodoulou | Spyridon |
| Chui Hom Lap | Jeffrey |
| Chung | Alex |
| Ciabatti | Giulia |
| Cicerchia | Pierfranco Maria |
| Ciftci | Ahmet Burak |
| Cifuentes-Rodenas | José Andrés |
| Cigagna | Luca |
| Cillara | Nicola |
| Ciolli | Maria Giovanna |
| Cipressi | Chiara |
| Cirillo | Bruno |
| Citgez | Bulent |
| Claramonte-Bellmunt | Olga |
| Clark | Mhairi |
| Clifford | Rachael |
| Cohen | Hugo |
| Coladonato | Massimiliano |
| Colak | Elif |
| Colás-Ruiz | Enrique |
| Collera Ormazábal | Pablo |
| Collins | Patrick |
| Colombari | Renan Carlo |
| Connelly | Tara |
| Cooke | Fiachra |
| Corcione | Francesco |
| corcione | gregorio |
| Córdova-García | Diego |
| Correa Bonito | Alba |
| Corso | Julian |
| Coşkun | Mümin |
| Costi | Renato |
| Cotronea | Carmelo |
| Crespi | Michele |
| Cribb | Benjamin |
| Crisafi | Daniel |
| Crisafi | Daniel |
| Cross | Katie |
| Crozier | Joseph |
| Cruikshank | Naomi |
| Curl-Roper | Thomas |
| Currò | Giuseppe |
| Curto López | Javier |
| Cuypers | Emma |
| Dale | James |
| D'Aloisio | Giordana |
| Danias | Nikolaos |
| Danwaththa Liyanage | Aloka Suwanna |
| Daoud | Mohammed |
| Darwich | Ayman |
| Dasilva | Louise |
| Däster | Silvio |
| Davakis | Spyridon |
| Davey | Matthew G |
| Davey | Martin S |
| David | Bryony |
| Davies | Ioan |
| D'avino | Raffaele |
| Davis | Kurt |
| Davis | George Neelankavil |
| Dawoud | Mostafa |
| De Andrés-Asenjo | Beatriz |
| de Gheldere | Charles |
| De Padua | Cristina |
| De Palma | Giovanni Domenico |
| De Paola | Gilda |
| De Toma | Giorgio |
| Deeknah | Abdulqudus |
| Del Rio | Paolo |
| Delgado Búrdalo | Livia |
| Delimpalta | Christina |
| Demirli Atici | Semra |
| Dhavala | Pooja |
| Di Nuzzo | Maria Michela |
| Di Saverio | Salomone |
| Diab | Jason |
| Diaz | Nicolas Romario |
| Díaz Gómez | Daniel |
| Díaz Pérez | Beatriz |
| Díaz San Andres | Beatriz |
| Dibra | Rigers |
| Dickerson | Luke |
| Díez-Alonso | Manuel |
| Dikicier | Enis |
| Dimitroulis | Dimitrios |
| Din | Farhat VN |
| Doganay | Emre |
| Doheim | Mohamed Fahmy |
| Dölzer | Lisa |
| Donigiewicz | Urszula |
| Douba | Zain |
| Doudin | Emad |
| Douglass | Ben |
| Drozdov | Evgeniy |
| Dubois | Marc |
| Dudek | Joanna |
| Dudi-Venkata | Nagendra |
| Duff | Sarah |
| Durán Muñoz-Cruzado | Virginia María |
| Duval | Jean-Luc |
| Dwidar | Oliver |
| Earley | Helen |
| East | Simon |
| Ebrahim | Saarah |
| Ebrahim | Mohamed |
| Edwards Murphy | Amy |
| Ejtehadi | Farshid |
| Ektiren | Mehmet |
| Elhadi | Muhammed |
| El Salawi | Omar |
| El Tohamy | Ayman |
| El Zaafarany | Ahmed |
| El-ashqar | Dina |
| Elbadawy | Merihan A. |
| Elbahnasawy | Mohamed |
| Elbahnasawy | Mohamed |
| El-Dhuwaib | Yesar |
| Elhadi | Muhammed |
| Elfeki | Hossam |
| Elhajdawe | Fras |
| Elkomy | Osama |
| Elniel | Mohammed |
| Elsabagh | Abdallah |
| Elsaid | Mirna |
| Elsayed | Ahmed |
| Elshami | Mohaemedraed |
| Elshennawy | Eslam |
| Elwan | Ayman |
| Emile | Sameh |
| Emmanuel | Klaus |
| En Oh | Ke |
| English | Caroline Louise |
| Enoch | Elizabeth |
| Entwistle-Thompson | Alexandra |
| Epifani | Angelo Gabriele |
| Eraslan | Huseyin |
| Erşen | Ogün |
| Espada Fuentes | Francisco Javier |
| Espi-Macias | Alejandro |
| Essa Tohamy | Tarek |
| Essam | Esmail |
| Estaire-Gómez | Mercedes |
| Fabbri | Nicolò |
| Fakhrul-Aldeen | Mohamed |
| Fannon | Noor |
| Fannon | Aseel |
| Fardanesh | Armin |
| Farquharson | Barnaby |
| Faulkner | Gemma |
| Faux | Will |
| Fellows | David |
| Feo | Carlo V. |
| Feria-González | Ana María |
| Fernández López | Lazaro Javier |
| Fernandez Martínez | María |
| Fetiha | Mohammed |
| Figueroa | Rafael |
| Firat | Necatin |
| Flatman | Michael |
| Flores Clotet | Roser |
| Foley | Katarina |
| Foppa | Caterina |
| Forero-Torres | Alexander |
| Fournier | Ian |
| Fowler | Hayley |
| Francone | Elisa |
| Franklyn | Joshua |
| Franzini | Christan |
| Frasson | Matteo |
| Freed | Ebru |
| Frontali | Alice |
| Frountzas | Maximos |
| G. Sayed | Esraa |
| Gadea-Mateo | Ricardo |
| Galiffa | Giampaolo |
| Gallo | Gaetano |
| Gamal | Mohamed |
| Ganesan | Nityanandan |
| Ganguly | Timothy |
| Garbarino | Sabrina |
| Garcés Palacios | Diana Sofía |
| Garcia Marin | Jose Andrés |
| García Muñoz | Patricia |
| García Septiem | Javier |
| Garcia-Chavez | Hector |
| García-Niebla | Jennifer |
| Gardiner | Padraig |
| Garg | Artu |
| Garofalidou | Tatiana |
| Garoufalia | Zoe |
| Gasser | Elisabeth |
| Gates | Zoe |
| Gattolin | Andrea |
| Gennari | Silvia |
| Gentilli | Sergio |
| Georgiou | Konstantinos |
| Gerdes | Stephan |
| Ghanbari | Amir |
| Ghanem | Ahmed |
| Ghignone | Federico |
| Gialamas | Eleftherios |
| Gijón Moya | Fernando |
| Gill | Sonia |
| Gill | Gurjot |
| Giménez Francés | Clara |
| Gimeno Calvo | Francisco Alberto |
| Giovenzana | Marco |
| Giuffrida | Mario |
| Giuffrida | Maria Carmela |
| Giuliani | Domenico |
| Giuliani | Beatrice |
| Giuliani | Antonio |
| Gómez Díaz | Carlos Javier |
| Gómez-Sanz | Tania |
| Gonullu | Emre |
| González Hernández | Sergio |
| Grandjean | Steven |
| Grassia | Sebastiano |
| Grechenig | Michael |
| Green | Suzie |
| Green | Dylan |
| Grimaldi | Sergio |
| Grosek | Jan |
| Grossi | Ugo |
| Groundwater | Ellen |
| Gruber | Ricarda |
| Grünbart | Martin |
| Guariglia | Claudio Antonio |
| Guboug | Ali |
| Guendil | Boumediene |
| Guerra | Bayron |
| Gulcek | Emre |
| Guler | Sertaç Ata |
| Guneyli | Cem |
| Gupta | Sapna |
| Gupta | Vivek |
| Gürtler | Thomas |
| Gut | Anna Eleonora |
| Guven | Onur |
| Guy | Richard |
| Habash | Elham |
| Hackett | James |
| Häivälä | Reetta |
| Hajirawala | Luv |
| Halle-Smith | James |
| Hamadi | Haider |
| Hamdan | Alaa |
| Hamed | Mazin |
| Hamid | Hytham K. S. |
| Hammad | Farah |
| Hamza | Hamza |
| Hamza | Amr |
| Handa | Siddhartha |
| Harivallavan | Nagendiram |
| Harmantepe | Tarik |
| Harris | Dean |
| Hart | Alex |
| Hasan | Dina |
| Hasırcı | İsmail |
| Hassam | Mohamed |
| Hassan | Mohamed Mare'y |
| Hassan | Mohammed |
| Hayward | Abigail |
| Hearle | Joseph |
| Helley | Michael |
| Hemadasa | Niroshini |
| Henniger | Georg |
| Herbert | Geraint |
| Hernández-Juara | Pilar |
| Herrero Muñoz | Irene |
| Hess | Gabriel F. |
| Hewett | Peter |
| Heywood | Nick |
| Hickey | Lorraine |
| Hijazin | Nadeen |
| Hijazin | Marleen |
| Hill | James |
| Hill | Arnold |
| Hine | Rachael |
| Hmeidan | Majedah |
| Hogan | Aisling M |
| Hollington | Paul |
| Horisberger | Karoline |
| Horwood | James |
| Hosfield | Thomas |
| Hosking | Rachel |
| Howe | Louise |
| Howie | Emma |
| Hoyos-Torres | Alejandro |
| Hsabo | Elmuiz |
| Hudson | Victoria |
| Hughes | James |
| Humayun | Quasim |
| Humes | David |
| Husain | Najam |
| Husain | Zain |
| Hussain | Aimatnuddin Husairi |
| Huth | Marcus |
| Iacomino | Alessandro |
| Iannone | Immacolata |
| Ibrahimli | Arturan |
| Incollingo | Paola |
| Ioannidis | Orestis |
| Iosifidis | Pavlos |
| Iqbal | Atif |
| Isa | Alaa |
| Isleem | Wejdan |
| Ismail | Iyad |
| Issa | Mohamed |
| Izquierdo-Moreno | Ana |
| Jacqmin | Geoffrey |
| Jamal Ghmagh | Reem |
| Javid | Zahra |
| Jayarajah | Umesh |
| Jezieniecki | Carlos |
| Jia | Kevin |
| Jichi | Tarik |
| Jimenez | Cristina |
| Jiménez Carneros | Virginia |
| Jiménez Miramón | Francisco Javier |
| Jimenez-Gomez | Luis Miguel |
| Jiménez-Higuera | Elisa |
| Jobran | Rania |
| Johnson | Meredith P. |
| Johnston | Sean |
| Jones | Robert P. |
| Jones | Sian |
| Jones | Andrew |
| Jorgensen | Lars Nannestad |
| Joshi | Heman |
| Jover Navalón | Jose Maria |
| Jovine | Elio |
| Kacimi | Salah Eddine |
| Kadamani | Akram |
| Kadir | Bryar |
| Kafka-Ritsch | Reinhold |
| Kalogiannis | Evangelos |
| Kampourakis | Christos Antonios |
| Kang | Gurpawan |
| Kang | Mandeep |
| Kanna | Sanad |
| Kanou | Loay |
| Kara | Yasin |
| Karabulut | Kerim |
| Karaca | Berkay Enes |
| Karamitsau | Evangeline |
| Karamitsou | Aikaterini |
| Karategos | Athanasios |
| Kardassis | Dimitrios |
| Karderirinis | Irene |
| Karim | Seiver |
| Karout | Lina |
| Kattakayam | Arjun |
| Kauppila | Joonas |
| Kaur | Mandeep |
| Kaya | Tayfun |
| Kaya | Cemal |
| Kayode-Nissi | Victor |
| Kayyal | Mohammed Yasser |
| Keeler | Barrie |
| Keller | Deborah S |
| Kennett | Jessica |
| Kerin | Michael J |
| Khafagy | Wael |
| Khalifa | Alaa |
| Khalifa | Haneen |
| Khalil | Mohammed |
| Khalil | Aoff |
| Khalil | Omar |
| Khalil | Ahmed Aly |
| Khamees | Almu'atasim |
| Khan | Jan |
| Khan | Fatma |
| Khan | Azam |
| Khan | Jim |
| Khoza | Charlene |
| Kilinc Tuncer | Gizem |
| Kılınç | Ahmet |
| Kleeff | Jorg |
| Klose | Johannes |
| Klyachman | Leslie |
| Klyachman | Leslie |
| Knowles | Charles |
| Knowles | Hannah |
| Ko | Emily |
| Koh | Hoey |
| Kokoropoulos | Panagiotis |
| Kollias | Victoria |
| Komolafe | Segun |
| Kontaxi | Ourania |
| Korkut | Mustafa |
| Koshel | Andrey |
| Košir | Jurij Aleš |
| Košir Božič | Tajda |
| Kotsifa | Eugenia |
| Kouskos | Efstratios |
| Kozman | Mathew |
| Kronberger | Irmgard E. |
| Kroon | Hidde |
| Kudchadkar | Shantata |
| Kumar | Dileep |
| Kumar | Sidharth |
| Kyriacou | Harry |
| Kyriakidis | Dimitrios |
| Kyriakopoulos | Georgios |
| Labró Ciurans | Meritxell |
| Lahlooh | Raghad Abed-Allateef |
| Lait | Emily |
| Lam | Kit |
| Landsweerdt | Simon |
| Lapolla | Pierfrancesco |
| Larentzakis | Andreas |
| Larri | Samuel |
| Lasheen | Omar |
| lauricella | sara |
| Lawday | Samuel |
| Ledda | Virginia |
| Lee | Katherine |
| Lee | Crystal |
| Lee | Matthew |
| Lefroy | Rebecca |
| Lena | Adriana |
| Lenzi | Elisa |
| Leonard | Eliot |
| Leventoglu | Sezai |
| Lewis-Lloyd | Christopher |
| Li | Nicolas |
| Liao | Christopher |
| Lieberman | Anna |
| Lim | Sean Tee |
| Lim | Jeffrey |
| Lim | Michael |
| Lima Pinto | Francisca |
| Lippolis | Giuseppe |
| Lisi | Giorgio |
| Liu | Jianliang |
| Livingston | Charles |
| Lloyd | Angus |
| Loganathan | Santhosh |
| Lombardi | Raffaele |
| Longo | Alberto |
| Lopesino González | Jose María |
| López Morales | Pedro |
| Lourido | Ana María |
| Loutzidou | Lydia |
| Luglio | Gaetano |
| Lunevicius | Raimundas |
| Lyons | Thomas |
| Maccabe | Thomas |
| Macleod | Anne |
| MacRury | Marion |
| Maffione | Francesco |
| Magee | Sean |
| Magill | Laura |
| Mahapatra | Sunanda |
| Maher | Ciara |
| Mahmoud | Omar |
| Maiuri | Vincenzo |
| Mäkäräinen-Uhlbäck | Elisa |
| Mäkelä-Kaikkonen | Johanna |
| Malcolm | Francesca |
| Malvaux | Philippe |
| Mansour | Bassam |
| Mantoglu | Baris |
| Manu | Nichola |
| Marano | Alessandra |
| Marco | Caricato |
| Marcu | Vasilica |
| Mariani | Nicolò Maria |
| Marino | Fabio |
| Marinos | Spyros |
| Marks | Bertie |
| Maroli | Annalisa |
| Marra | Ester |
| Marsanic | Patrizia |
| Martin-Arevalo | Jose |
| Martinez Alegre | Javier |
| Martinez-Iglesias | Marta |
| Martinez-Moreno | Jose Luis |
| Mascianà | Gianluca |
| Masetti | Michele |
| Massey | Lisa |
| Massie | Eleanor |
| Math | Suraj |
| Mathur | Pawan |
| Matías-García | Belén |
| Mattar | Ahmed |
| Maung | Min |
| Maurus | Christine |
| Mawhinney | Jamie |
| Mazzotta | Erica |
| Mazzotti | Federico |
| McAvoy | Andrew |
| McAvoy | Dean |
| McClintick | Jessica |
| McClune | Anna |
| McColl | Gillian |
| McCullough | Peter |
| McDermott | Frank D |
| McGuigan | Mari-Claire |
| McMahon | Ross |
| McNair | Angus |
| Md Hashim | Mohd Nizam |
| Mehmood | Iftikhar |
| Mendoza-Moreno | Fernando |
| Menegat | Nevio |
| Meneghini | Simona |
| mengual-ballester | Monica |
| Meric | Serhat |
| Merlini | Ilenia |
| Meza Cabrera | Maria Del Mar |
| Mezead | Mohmmad |
| Michail | Marina R |
| Michalopoulos | Nikolaos |
| Migliore | Marco |
| Milano | Egidio |
| Mills | Emily |
| Mingoli | Andrea |
| Mitteregger | Martin |
| Mittermair | Christof |
| Mizrahi | Joseph |
| mizrahi | joseph |
| Moctezuma-Velázquez | Paulina |
| Mohamed Dahy | Toqa |
| Mohamed Hussein | Aliae |
| Mohamed | Shaymaa Elsayed |
| Mohamedahmed | Ali Yasen Y |
| Mohammad Azmi | Mohd Azem Fathi |
| Mohammed | Nada Esmaeel |
| Mohd Yunus | Mohamad Fadli |
| Moitzi | Gabriele |
| Montali | Filippo |
| Monteiro de Barros | James |
| Montemurro | Leonardo |
| Monti | Marco |
| Montroni | Isacco |
| Monzur | Farah |
| Monzur | Farah |
| Moon | Jeongyoon |
| Moore | Tom |
| Morad | Afnan |
| Morales Diaz | Samuel |
| Morello | Alessia |
| Moreno Suero | Francisco |
| Morgan | Matthew |
| Morini | Andrea |
| Moro-Valdezate | David |
| Morrison-Jones | Victoria |
| Morton | Alastair |
| Mosos | Monica Briguitte |
| Mosquera | Manuel |
| Mostafa | Muhanad |
| Mostafa | Hasan |
| Mostafa | Ahmed |
| Mostafa | Hasan |
| Moug | Susan |
| Mpitsianis | Stefanos |
| Msherghi | Ahmed |
| Mulligan | Eabhard |
| Muñoz Camarena | Jose Manuel |
| Muñoz-Sornosa | Ernesto |
| Muratore | Andrea |
| Murgese | Alessandra |
| Murphy | Brenda |
| Musallam | Marah |
| Mustafa | Hamid |
| Myer | Adam |
| Myer | Adam |
| Nafea | Ahmed |
| Naguib | Mostafa Mahmoud |
| Naidoo | Kamil |
| Naik | Lesley-Ann |
| Nair | Gavin |
| Nair | Dheepa |
| Ñañez Pantoja | María Alejandra |
| Napolitano | Milena |
| Nasser | Hussam |
| Naumann | David |
| Navio | Ana |
| Neary | Peter |
| Nervini | Andrea |
| Ng | Vivian |
| Ng | Sherwin |
| Ng | Samantha |
| Nicastro | Vincenzo |
| Nicholson | Gary |
| Nikaj | Herald |
| Nikiteas | Nikolaos |
| Ninkovic | Marijana |
| Nofal | Heba |
| Nolan | Ryan |
| Novak | Ivana |
| novi | alessandra |
| Nowak | Kai |
| Ntampakis | George |
| Nuñez O´Sullivan | Sara |
| Nyssen | Marie |
| O'Brien | Stephen |
| Obrowski | Stephanie |
| Okoth | Kelvin |
| Oldani | Massimo |
| Oldfield | Fran |
| Ollier | Misti |
| Omeroglu | Sinan |
| Ong | Daniel |
| Opocher | Enrico |
| Orangio | Guy |
| Ordoñez | Victor Manuel |
| O'Reilly | Colum |
| Osman | Mohmed |
| Osorio Ramos | Alexander |
| Othman | Ahmad Riyad |
| Othman | Muhammad Faeid |
| Ottl | Stephanie |
| Ouzounidis | Nikolaos |
| Ovejero-Merino | Enrique |
| Owda | Saed |
| Oyanik | Ahmet Faruk |
| Özata | Ibrahim Halil |
| Ozaydın | Safa |
| Özcan | Adem |
| Ozdemir | Kayhan |
| Özden | Sabri |
| Özocak | Aysegul Bahar |
| Özoran | Emre |
| Padilla-Valverde | David |
| Pagano | Gianluca |
| Pagliai | Lorenzo |
| Pagliai | Lorenzo |
| Palios | Ifaistion |
| Palomares-Casasus | Sara |
| Papadoliopoulou | Maria |
| Papaeleftheriou | Stavroula |
| Papagni | Vincenzo |
| Papandrea | Matteo |
| Papazarkadas | Xenofon |
| Paraoan | Marius |
| Pardo Lopez | Sara |
| Parfitt | Charlotte |
| Park | Soo Rin |
| Parlanti | Daniele |
| Parmar | Chetan |
| Parra Baños | Pedro Antonio |
| Passuello | Nicola |
| Pata | Francesco |
| Patel | Panna |
| Patel | Mahul |
| Patel | Ben |
| Patel | Meera |
| Pathmanathan | Keren |
| Paul | Emila |
| Pavão | Tiago |
| Pavesi | Franco |
| Payne | Christopher |
| Pearce | Lyndsay |
| Pegna | Victoria |
| Pellicer Franco | Enrique |
| Peltrini | Roberto |
| Peña Ros | Emilio |
| Peravali | Rajeev |
| Perez | Carlos J- |
| Pérez-Sánchez | Luis Eduardo |
| Perez-Santiago | Leticia |
| Perivoliotis | Konstantinos |
| Perrone | Gennaro |
| Perrone | Fabrizio |
| Perry | Rita |
| Pesce | Antonio |
| Pestrin | Olivia |
| Petracca | Gabriele |
| Pettitt | Michala |
| Pezzolla | Francesco |
| Pezzuto | Anna |
| Phumiphakmethakul | Pawanda |
| Picarella | Pietro |
| Picciariello | Arcangelo |
| Pindozzi | Fioralba |
| Pinkney | Thomas |
| Pinto | José |
| Pipitone Federico | Nicoletta sveva |
| Pirozzi | Nello |
| Pisani Ceretti | Andrea |
| Polat | Suleyman |
| Polyak | Tatyana |
| Ponzo | Paola |
| Pop | Ionut |
| Porfidia | Raffaele |
| Porta | Andrea |
| Pou Macayo | Sara |
| Poulios | Efthimios |
| Pramanik | Sanjeev |
| Pranesh | Nagarajan |
| Preece | Ryan |
| Presl | Jaroslav |
| Prieto | Fernando |
| Primo | Vicent |
| Proud | David |
| Puerari | Gian Attilio |
| Putzu | Giaime |
| Qasim | Ahmad |
| Quddus | Abdul |
| Quiroga-Valcárcel | Ana |
| Quneis | Ossaid |
| Rabie | Mohammed |
| Racine | Michael |
| Raheel | Muhammad |
| Rahman | Rafid |
| Rai | Subash |
| Rajaretnam | Niroshini |
| Rajput | Kunal |
| Rajput | Kunal |
| Ramadan | Salma |
| Ramadan | Widad |
| Ramasamy | Sadhasivam |
| Ramirez | Juan Sebastian |
| Ramirez | Natalia |
| Ramirez | Daniel Mauricio |
| Ramirez Caballero | Ester |
| Ramírez Faraco | María |
| Ramos Rodriguez | Jose Luis |
| Ramos Soler | Francisco |
| Ramsanahie | Anthony |
| Rangaiah | Chandrashekar |
| Rankin | Adeline |
| Rashid | Adil |
| Raskin | Elizabeth |
| Rateb | Fares |
| Ravi | prabhu |
| Raza | Imran |
| Rees | Adam |
| Reeves | Nicola |
| Reggiori | Alberto |
| Rehman | Saad |
| Rehman | Mutee |
| Reitano | Elisa |
| Rengifo | Carla |
| Rhayim | Roaa |
| Riaz | Samreena |
| Ricciardi | Pietro |
| Riedl | Peter |
| Riente | Francesco |
| Rigby | Sarah |
| Rimmer | Lara Jane |
| Rimonda | Roberto |
| Rivera Castellano | Javier |
| Rocchi | Paolo |
| Rodrigo | Vitharanage Srimantha Dewsiri |
| Rodriguez | Pedro |
| Rodríguez Sánchez | Ana |
| Rojas-Khalil | Yesenia |
| Rollo | Alessio |
| Román | Carlos |
| Romano | Angela |
| Romano | Lucia |
| Romanou | Evdokia |
| Roncone | Arturo |
| Ronellenfitsch | Ulrich |
| Rooney | Siobhan |
| Rotas | Ioannis |
| Rottoli | Matteo |
| Rozwadowski | Sophie |
| Ruiz-Soriano | María |
| Russo | Giulia |
| Ryan | Éanna J |
| Ryan | Jessica |
| Saavedra | Juan David |
| Sabbar | Mohammed |
| Sabboobeh | Sarah |
| Sabry | Fady |
| Sagar | Jayesh |
| Sagoo | Harkiran |
| Sahin | Can |
| Şahin | Alpaslan |
| Sainz-Hernández | Juan |
| Saleh | Ahmed |
| Saleh | Mahmoud |
| Salgado-Nesme | Noel |
| Salhan | Jyoti |
| Salomone | Sara |
| Salvemini | Carlo |
| Samadov | Elgun |
| Sambucci | Daniele |
| Sami | Sharukh |
| Sammarco | Giuseppe |
| Sammour | Tarik |
| Samy | Hossam |
| Sanchez | Estefania |
| Sánchez Arteaga | Alejandro |
| Sánchez-Gollarte | Ana |
| Sánchez-Peláez | Daniel |
| Sancho-Muriel | Jorge |
| Sanchon Fructuoso | Lorena |
| Santandrea | Letizia |
| Santillan | Mateo |
| Santoro | Giulio Aniello |
| Sapienza | Paolo |
| Sapre | Dimple |
| Sari | Ahmet Can |
| Sarodaya | Varun |
| Sartarelli | Lodovico |
| Sarveswaran | Janahan |
| Sasia | Diego |
| Sauvain | Marc-Olivier |
| Savoie-Hontoria | María |
| Sayad | Reem |
| Scaltrini | Francesca |
| Schiller | Philipp |
| Schirnhofer | Jan |
| Schmid | Alexandra |
| Segalini | Edoardo |
| Seitinger | Gerald |
| Sevdi | Salih |
| Sexton | Gerard |
| Sgrò | Alessandro |
| Shabana | Amanda |
| Shabbir | Jamshed |
| Shahzad | Khalid |
| Shalaby | Mostafa |
| Shams | Ola |
| Shamsher | Shilpa |
| Sharma | Natalie |
| Sharma Khatiwada | Aagat |
| Sharpe | Alexandra |
| Shebli | Baraa |
| Shehada | Anas |
| Shehata | Mostafa A. |
| Shehata | Zak |
| Shihab | Oliver |
| Shinkwin | Michael |
| Sidiropoulos | Theodoros |
| Simeonidis | Savvas |
| Simianu | Vlad V. |
| Simondi | Daniele |
| Şimşek | Gürcan |
| Singhal | Tarun |
| Sinha | Ankit |
| Skotsimara | Antonia |
| Smart | Christopher J |
| Smart | Neil J |
| Smerat | Mohammad |
| Smith | Dave |
| Smolarek | Sebastian |
| Sodde | Peter |
| Soh | Jien Yen |
| Soldini | Gabriele |
| Soler Frias | Joan Ricard |
| Somuncu | Erkan |
| Soria Aledo | Víctor |
| Soriano | Celine R. |
| Sorocovici | Rodica |
| Soto Montesinos | Cristina |
| Soysal | Savas D. |
| Sperber | Jonas |
| Spinelli | Antonino |
| Stavrou | Gregor Alexander |
| Stefan | Samuel |
| Stefanescu | Iulia Alexandra |
| Stefanova | Irena |
| Steiner | Florian |
| Stokes | Emily |
| Storey | Sharon |
| Stratakis | Konstantinos |
| Stubbs | Benjamin |
| Stucchi | Claudia |
| Suhardja | Thomas |
| Sundhu | Matthew |
| Swamanatham | Christie |
| Syed | Ali Waris |
| Sylla | Patricia |
| Taffurelli | Giovanni |
| Taggarsi | Meghana |
| Talab | Tamie |
| Tallón Aguilar | Luis |
| Tamara | Jorge Leonardo |
| Tamini | Nicolò |
| Tanal | Mert |
| Tanzanu | Marta |
| Tapuria | Niteen |
| Tartas Ruiz | Aurea |
| Tasende-Presedo | Marta |
| Tashan | Nashwan |
| Tatar | Ozan Can |
| Tawfik | Ahmed |
| Tayyem | Raed |
| Testa | Valentina |
| Thaha | Mohamed |
| Theodoropoulou | Katerina |
| Thomas | Rhys |
| Thomas | Pradeep |
| Thomas-Williams | Emily |
| Thompson | Jessica |
| Thoukididou | Sarah |
| Tinoco González | Jose |
| Toale | James |
| Tokocin | Merve |
| Tokocin | Onur |
| Tomažič | Aleš |
| Tonini | Valeria |
| Torkington | Jared |
| Tornese | Deborah |
| Torrado | Maria Alejandra |
| Totis | Mauro |
| Traeger | Luke |
| Travaglio | Elisabetta |
| Triantafyllou | Alexandra |
| TRIANTAFYLLOU | Tania |
| Trigiante | Giuseppe |
| Tropeano | Francesca Paola |
| Trujillo | Jeancarlos |
| Tuckey | Laurel |
| Tufan | Aydin Eray |
| Tüfekçi | Tutku |
| Tuñon Fequánt | Carlota Isabel |
| Turan | Ersin |
| Turina | Matthias |
| Turk | Ismael |
| Tzovaras | George |
| Uc | Can |
| Ugolini | Giampaolo |
| Uludag | Mehmet |
| Ulutaş | Mehmet Eşref |
| uprak | Tevfik Kıvılcım |
| Uraiqat | Ahmad |
| Uranitsch | Stefan |
| Uslu | Gülberk |
| Utkan | Nihat Zafer |
| Uyanik | Mustafa Safa |
| Valério | Fernando |
| Valero | Camilo |
| valero Navarro | Graciela |
| Valle Rubio | Ainhoa |
| Valverde-Mantecón | José Miguel |
| van de Hoef | Dayna |
| Van Vaerenbergh | Wim |
| Vassiliu | Pantelis |
| Vather | Ryash |
| Vaughan-Shaw | Peter G |
| Ventham | Nicholas T |
| Vera-Mansilla | Cristina |
| Vescio | Giuseppina |
| Viamontes Ugalde | Francisco Eduardo |
| Vijay | Vardhini |
| Vila-Zárate | Cristina |
| Vimalachandran | Dale |
| Virgilio | Edoardo |
| Vitone | Louis |
| Vitón-Herrero | Rebeca |
| Vitovska | Eva |
| Vrakopoulou | Gavriella-Zoi |
| Vuagniaux | Aurelie |
| Wadham | Bianca |
| Wallner | Elisabeth |
| Wally | Rim |
| Walters | Michael |
| Wan Mokhter | Wan Mokhzani |
| Warrag | Ibrahim |
| Watfah | Josef |
| Watson | Eleanor |
| Watson | Henry |
| Weiss | Helmut G. |
| West | Alex |
| Wheatstone | Sarah |
| Whitehouse | Arlo |
| Widyaningsih | Rizky |
| Wiesler | Benjamin |
| Williams | Olatoyosi |
| Williams | Gethin |
| Williams | Katherine |
| Wilson | Megan |
| Wimmer | Angela |
| Wong | Michael Pak-Kai |
| Wookey | Rebecca |
| Woyton | Michal |
| Wright | Deborah |
| Wyatt | James |
| Yalcinkaya | Ali |
| Yao | Lucy |
| Yassin | Nuha |
| Yeboah | Kwasi |
| Yeoh | Adrian |
| Yeşilyurt | Değercan |
| Yetkin | Sitki Gurkan |
| Yip | Cheerong |
| Yiu | Andrew |
| Yoldas | Tayfun |
| Younis | Soha |
| Yousef | Yousef A |
| Zafar | Muneeb |
| Zaghloul | Karim |
| Zakaria | Andee Dzulkarnaen |
| Zakaria | Zaidi |
| Zaman | Shafquat |
| Zambon | Martina |
| Zanus | Giacomo |
| Zapsalis | Konstantinos |
| Zattoni | Davide |
| Zazo | Aya |
| Zazo | Rama |
| Zhang | Jennifer |
| Ziyad | Ashwaq |
| Zor | Omer Batuhan |
